# Electrochemical and Photoredox Catalysis for Constructing 5,5‐Spirocycles via Reductive Activation of *N*‐alkoxyphthalimides for the Total Synthesis of (−)‐Cephalosporolide F

**DOI:** 10.1002/cssc.202501605

**Published:** 2025-09-15

**Authors:** Julio Romero‐Ibañez, Karen A. Guarneros‐Cruz, Fernando Sartillo‐Piscil, Bernardo Antonio Frontana‐Uribe

**Affiliations:** ^1^ Departamento de Química Orgánica Centro Conjunto de Investigación en Química Sustentable UAEMex‐UNAM Km. 14.5 Carretera Toluca‐Atlacomulco Toluca C.P. 50200 Estado de México México; ^2^ Departamento de Química Orgánica Instituto de Química, Universidad Nacional Autónoma de México Circuito exterior Coyoacán 04510 Ciudad de México; ^3^ Facultad de Ciencias Químicas Benemérita Universidad Autónoma de Puebla. 72570 Puebla México

**Keywords:** green chemistry, *N* ‐alkoxyphthalimides, organic electrosynthesis, photocatalysis, spirocyclization

## Abstract

The electrosynthetic (ES) approach employing rapid alternating polarity electrolysis and blue LED photoredox catalysis (PRC) is revised and compared in the synthesis of [5,5]‐spiroketals through a tandem reductive activation of *N*‐alkoxyphthalimides to alkoxy radical, followed by hydrogen atom transfer, and spirocyclization sequence. The role of leaving group and redox conditions is explored in the electrochemical transformation, revealing that diphenylphosphate‐derived substrates exhibit superior performance. Both methodologies enable the stereoselective synthesis of (−)‐Cephalosporolide F from the chiral pool, with a slightly higher yield for the PRC involving an iridium catalyst. This study showcases the potential use of electrochemical and photochemical green redox methodologies for radical‐mediated transformations and late‐stage synthesis of natural products, avoiding toxic stannyl reagents and bypassing expensive metal‐based catalysts. These findings support the development of more sustainable synthetic strategies for complex natural products while acknowledging the use of CH_2_Cl_2_ and tetrabutylammonium salts in the ES procedure.

## Graphical abstract

The spirocyclization of *N*‐alkoxyphthalimides carbohydrate derivatives through electrochemical and photocatalytic redox approaches is evaluated. The electrochemical conditions prove effective in promoting the formation of the [5,5]‐spiroketal core found in Cephalosporolide F.

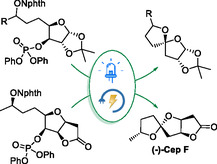

## 1 Introduction

**Figure 1:**
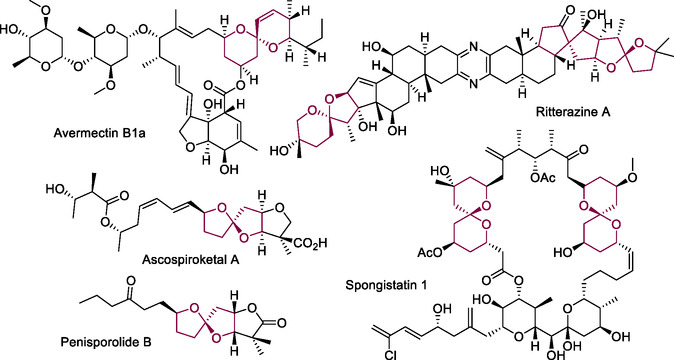
Natural products containing spiroketals frameworks.

Bicyclic organic systems fused by a single atom are called spirane or spiro compounds. This organic framework is found in a variety of natural and synthetic products, and due to its characteristic conformational rigidity, its incorporation is desirable in drugs to enhance physicochemical properties such as solubility, lipophilicity, or absorption, distribution, metabolism, and excretion properties.^[^ ^1^, ^2^, ^3^ ^]^ From the plethora of spiro compounds, spiroketal scaffolds are common in natural products with important biological activities.^[^ ^4^, ^5^, ^6^ ^]^ For instance, avermectins, a [6,6]‐spirocycle, possesses insecticidal and antiparasitic properties;^[^ ^7^, ^8^ ^]^ spongistatins are potent cancer cell growth inhibitors containing macrocyclic structures with two spirane units^[^ ^9^ ^]^; and the antitumoral inhibitors, cephalostatins and ritterazines, display a spirocyclic variability of [6,5]‐ and [5,5]‐spiroketalic functionalities on their steroidal framework (**Figure** **1**).^[^ ^10^ ^]^ Likewise, spiroketal frameworks are present in small molecules such as certain cephalosporolides, penilosporolides, and ascospiroketals, which exhibit antimicrobial and anti‐inflammatory activities (Figure 1).^[^ ^11^, ^12^, ^13^ ^]^

Accordingly, developing efficient synthetic methods for constructing spiroketal skeletons has attracted the interest of the synthetic community.^[^ ^14^, ^15^, ^16^, ^17^, ^18^, ^19^ ^]^ Although the existing strategies are diverse, innovative approaches are expected to emerge focused on stereoselectivity and sustainability.^[^ ^20^, ^21^, ^22^, ^23^ ^]^

**Scheme 1:**
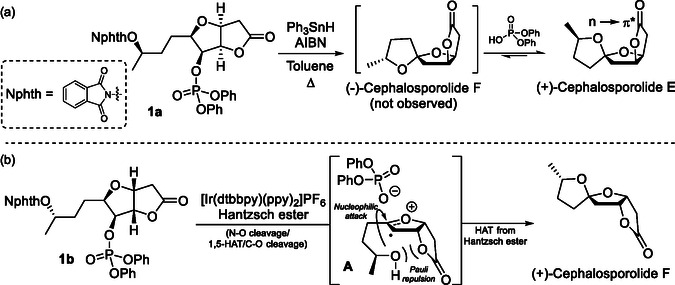
Stereoselective synthesis of a) Cephalosporolide E, and b) Cephalosporolide F.

In 2015, the Sartillo‐Piscil's group applied a tandem hydrogen abstraction–cyclization sequence, reported by Crich and Newcomb,^[^ ^24^ ^]^ in the stereoselective construction of a [5,5]‐spiroketal‐fused γ‐lactone framework to achieve the total synthesis of Cephalosporolide E (**Cep E**) under tin conditions, where a nonanomeric stabilization was identified as the key factor for the stereoselectivity (**Scheme** **1**a).^[^ ^25^, ^26^ ^]^ Meanwhile, a photoredox catalytic (PRC) approach enabled them to accomplish the stereoselective construction of Cephalosporolide F (**Cep F**), where the evaluated stereoelectronic model indicates a Pauli repulsion destabilization of the contact ion pair intermediate leading to the kinetic product (Scheme 1b).^[^ ^27^ ^]^

Furthermore, considering these previous reports and our efforts to develop sustainable methodologies with limited use of organostannyl compounds or expensive metal‐based catalysts, the application of electrochemically driven generation of alkoxyl radicals from convenient *N*‐alkoxyphthalimides was investigated to create carbohydrate‐derivative [5,5]‐spiroketalic skeletons. The use of electrosynthesis in annulation reactions has been revised and recognized as a promising green methodology;^[^ ^28^ ^]^ however, to date, there are no prior reports of electrochemical spiroketalization from carbohydrate‐derived *N*‐alkoxyphthalimides. The developed methodology was compared with the PRC, and after optimizing conditions, both were evaluated in the total synthesis of the natural compound Cephalosporolide F.

## 2 Results and Discussion

**Scheme 2:**
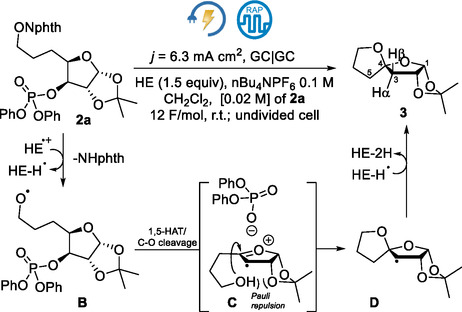
Electrochemically driven spirocyclization of NAPI **2a** to the spirocycle **3**.

The *N*‐alkoxyphthalimide (NAPI) **2a** was selected for the initial exploratory study of the electrochemically driven construction of a [5,5]‐spirocycle moiety^[^ ^29^ ^]^ (**Scheme** **2**). Like several NAPIs reported,^[^ ^30^, ^31^ ^]^ the cyclic voltammetry analysis of compound **2a** showed a characteristic irreversible peak at −1.72 V (vs Ag/Ag^+^, see SI), and the absence of reduction peaks at lower values that could lead to byproducts, indicating that the generation of the NAPI^•−^ anion radical is suitable to evolve to the corresponding oxygen‐centered radical.^[^ ^30^, ^31^ ^]^ For the electrochemical generation of alkoxyl radicals from NAPIs, rapid alternating polarity (RAP) electrolysis was used.^[^ ^32^, ^33^ ^]^ In these conditions, NAPI^•−^ and Hantzsch ester (HE) radical cation (HE^**•+**^) intermediates are produced rapidly on the same electrode interface, allowing their rapid interaction as was proposed initially for the PRC reaction mechanism.^[^ ^34^, ^35^ ^]^

After electrolysis in CH_2_Cl_2_ and purification by column chromatography, compound **3** was obtained in 10% yield (**Table** **1**, entry 1). The stereochemical assignment of the spiro atom was made considering: 1) that the ^1^H NMR spectrum of the spiro compound **3** did not match with the previously reported spectrum;^[^ ^29^ ^]^ 2) a NOESY experiment, in which an interaction between H3_β_ and H5 was not observed;^[^ ^36^ ^]^ 3) a diamagnetic anisotropic chemical shift effect, where the *trans*‐2,4‐dioxy substitution gives a small Δ*δ*_H3_ (*δ*_H3β_ – *δ*_H3α_ = ≈0.09 ppm),^[^ ^37^ ^]^ in comparison with the putative large Δ*δ*_H3_ (≈0.21 ppm) observed for the reported C4‐epimer with a *cis*‐2,4‐dioxy substitution, suggesting that the cyclization stereoselectivity is endorsed by a Pauli repulsion interaction (Scheme 2).

**Table 1:** Screening conditions for electrochemical spirocyclization of 2a.

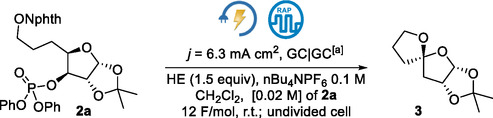		
Entrya)	Variations	Yieldb)
1	RAP 50 Hz	10%
2	RAP 50 Hz, CH_3_CN	N.R.c)
3	RAP 50 Hz, 2‐Me‐THF	N.R.c)
4	RAP 50 Hz, 2‐Me‐THF/CH_3_CN (8:2)	8%
5	RAP‐5 Hz	Traces
6	RAP‐20 Hz	22%
7	RAP‐30 Hz	5%
8	Pulsed alternating polarity (Upper pol time: 20 s; lower pol time: 10 s)	Traces
9	RAP‐20 Hz, 2‐Me‐THF/CH_3_CN (8:2)	16%
10	RAP‐20 Hz, 8 F	12%
11	RAP‐20 Hz, *j* = 3 mA cm^2^	N.R.c)
12	RAP‐20 Hz, *j* = 4.5 mA cm^2^	4%d)
13	RAP‐20 Hz, 2 eq HE	28%
14	RAP‐20 Hz, 4 eq HE	23%

a) GC = glassy carbon.

b) Isolated yield.

c) N.R. = no reaction; 84–90% of the starting material was recovered.

d) 70% of the starting material was recovered.

Afterward, reaction condition optimization revealed that other solvents did not provide better results (Table 1, entries 2–4). A yield improvement was observed when the polarity‐switching frequency was lowered to 20 Hz (Table 1, entries 5–7). Pulsed alternating polarity was ineffective (Table 1, entry 8). Running the reaction under a lower density current or with a lower charge did not lead to the consumption of **2a**, generating **3** in low yield (Table 1, entries 10–12). The increase of the amount of HE to 2 eq. resulted in a 28% yield of the spirocycle while higher amounts of HE were not beneficial (Table 1, entries 13 and 14).

**Scheme 3:**
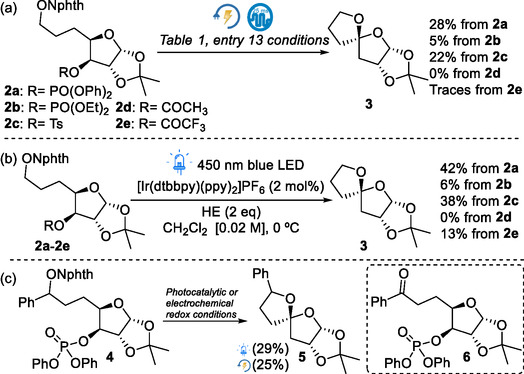
Spirocyclization of *N*‐alkoxyphthalimides a,b) **2** and c) **4** under photocatalytic and electrochemical redox conditions.

Since the reaction occurs through the heterolysis of the C—O bond, the nature of the leaving group plays a crucial role in the formation of the cyclic adduct. Therefore, we explored different leaving groups under the conditions described in Table 1, entry 13. Using a diethyl phosphate group resulted in the spirocycle **3** in lower yield, whereas the tosylate moiety gave moderate yield compared to that obtained with the diphenyl phosphate group (**Scheme** **3**a). As previously reported, weaker nucleofuges such as acetoxy failed to produce the heterocycle **2a**, and unexpectedly, the trifluoroacetoxy group resulted in a similar outcome. To further assess the formation of spirocycle **3** under photocatalytic redox conditions, the corresponding NAPIs **2a**‐**2e** were subjected to the parameters described in Scheme 3b, exhibiting similar reactivity with slightly improved yields. Additionally, when *N*‐benzyloxyphthalimide **4** was subjected to photocatalytic and electrochemical redox conditions, spirocycle **5** was obtained in a modest yield.^[^ ^38^ ^]^ Notably, neither the carbonyl‐containing substrate **6** nor any β‐fragmentation products were observed in the isolated mixtures (Scheme 3c). This is convenient for multifunctional or late‐stage functionalization of complex molecules.^[^ ^39^, ^40^ ^]^

**Scheme 4:**
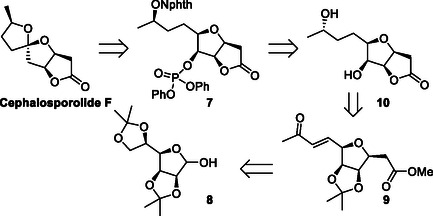
Retrosynthetic analysis of (−)‐Cephalosporolide F.

With the results obtained and anticipating a similar course of stereo‐induction, it was decided to evaluate the electrochemical methodology in the late‐stage spirocyclization of *N*‐alkoxyphthalimide **7** to access (−)‐Cephalosporolide F, as depicted in the retrosynthetic analysis of **Scheme** **4**. To achieve this, the *C*‐glycosylation of protected *D*‐mannose **8** with a stabilized ylide, followed by selective hydrolysis. The obtained compound would be transformed into ketone **9** after Malaprade oxidation and Wittig olefination. A subsequent stereoselective reduction, combined with acid‐promoted deprotection and lactonization, is expected to yield the corresponding lactone **10**. Finally, installing the *N*‐hydroxyphthalimide group under Mitsunobu conditions, along with hydroxyl phosphorylation under standard conditions, would produce the spirocyclization precursor **7**.

**Scheme 5:**
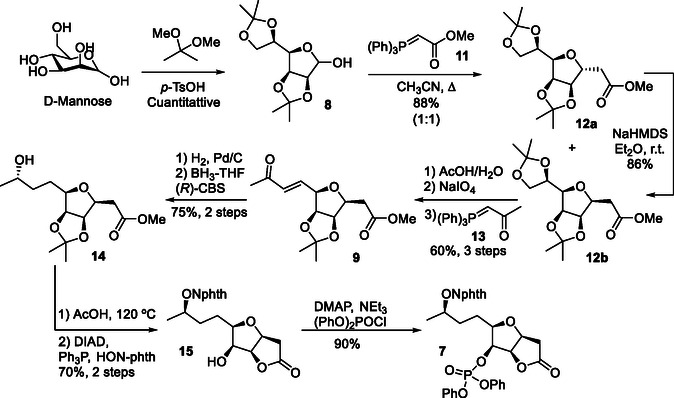
Synthesis of the *N*‐hydroxyphthalimide spirocyclization precursor **7**.

The synthetic route (**Scheme** **5**) began with the protection of *D*‐mannose using 2,2‐dimethoxypropane under acid‐catalyzed conditions, followed by a C‐glycosylation with phosphorus ylide **11**, which produced the diastereomeric furanosyl C‐glycosides **12a** and **12b** in 88% yield. Isomerization of **12a** under basic conditions furnished the thermodynamically more stable ester **12b** in 86% yield.^[^ ^41^ ^]^ Subsequently, the vicinal diol at C5 and C6 was liberated with AcOH, oxidizing cleavage using NaIO_4_, and the resulting aldehyde was olefinated with phosphorus ylide **13**, resulting in the formation of the corresponding α,β‐unsaturated ketone **9** in 60% overall yield. Hydrogenation of the double bond produced the corresponding saturated ketone **9**, which was stereoselectively reduced to the alcohol **14** in a 3:1 diastereomeric mixture, with a preference for the (*S*)‐isomer when using the (*R*)‐Corey–Bakshi–Shibata (CBS) catalyst.^[^ ^42^ ^]^ Compound **15** was then transformed into *N*‐alkoxyphthalimide **16** via a microwave‐assisted two‐step sequence involving acid‐catalyzed deprotection and lactonization, followed by the installation of the *N*‐hydroxyphthalimide moiety under Mitsunobu conditions. Finally, phosphorylation of **16** with diphenyl phosphoryl chloride yielded the spirocyclization precursor **7** (Scheme 5)

**Scheme 6:**
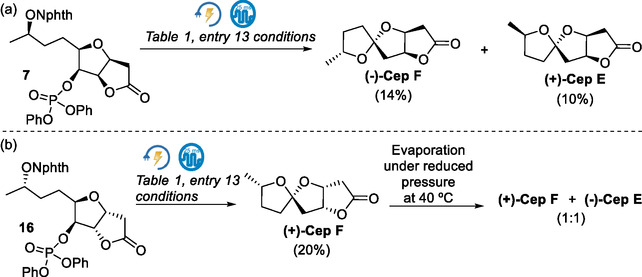
Electrochemically driven spirocyclization of *N*‐alkoxyphthalimides a) **7** and b) **16**.

We explored the reactivity of **7** under electrochemical conditions previously found, producing (−**)‐Cep F** and **(+)‐Cep E**, in 14% and 10% yield, respectively (**Scheme** **6**a). To further investigate this stereochemical outcome, the *N*‐alkoxyphthalimide **16** was prepared^[^ ^27^ ^]^ and submitted to electrochemical conditions, affording **(+)‐Cep F**, with traces of (−**)‐Cep E** detected by TLC (Scheme 6b). Moreover, when the pure NMR sample of **(+)‐Cep F** was evaporated under reduced pressure at 40 °C to record a solvent‐free spectrum, epimerization was observed, resulting in a roughly 1:1 mixture of **(+)‐Cep F** and (−**)‐Cep E** (Scheme 6b). This outcome is consistent with related cases reported in the literature,^[^ ^43^ ^]^ and is likely promoted by trace acidic impurities and thermodynamic equilibration, given the enhanced stability of Cephalosporolide E attributed to a nonanomeric stabilization effect ($n_{O}$ → $\pi_{C=O}^{*}$; vide supra).^[^ ^26^ ^]^ Thus, under electrochemical conditions, (+)‐Cephalosporolide F was formed stereoselectively, and the co‐isolation of (−)‐Cephalosporolide E suggests an epimerization event during the isolation process.^[^ ^44^ ^]^

From a practical standpoint, in terms of time and associated energy consumption, although the spirocyclization of **2a** and **4** under photoredox conditions showed a slightly better conversion yield with comparable reaction times (**Table** **2**, entries 1–4), the construction of the spiroketal in Cephalosporolide F/E through the electrosynthetic (ES) route provides lower yields than the photoredox approach, it requires shorter reaction time (≈18.8–20.6 h/mmol; entries 5 and 6); in some way, this compensates for the yield product equivalence photocatalytic alternative (≈80 h mmol^−1^; entry 7). These results show that the initial advantage in reaction time offered by electrosynthesis is not discouraging even if it shows low conversion, emphasizing the importance of assessing energy input relative to actual product output when comparing sustainable redox methodologies.

**Table 2:** Charge and/or time employed in the spirocyclization of **2a**, **4**, **7**, and **16**.

Entrya)	Substrate	Modality	Time [h mmol^−1^]	Charge [C mmol^−1^]
**1**	**2a**	Electro (RAP)	18.8 h	1158
**2**	**2a**	Photo (Ir (III), blue LED)	15 h	–
**3**	**4**	Electro (RAP)	18.8 h	1158
**4**	**4**	Photo (Ir (III), blue LED)	15 h	–
**5**	**7**	Electro (RAP)	18.8 h	1158
**6**	**16**	Electro (RAP)	20.6 h	1158
**7**	**16**	Photo (Ir (III), blue LED)	80 h^[^ ^27^ ^]^	–

a) All values were normalized to time per mmol and charge per mmol. See the Supporting Information for experimental and calculation details.

## 3 Conclusion

In summary, the generation of alkoxyl radicals from *N*‐alkoxyphthalimides employing RAP electrolysis proved to be efficient with the tandem hydrogen abstraction‐spirocyclization process for the synthesis of [5,5]‐spiroketals. In this study, both electrochemical and PRC strategies resulted in similar stereoselective outcomes, with the diphenylphosphatoxy group providing better results under both redox conditions. The chiral pool strategy employed to construct the *N*‐alkoxyphthalimide spirocyclization precursor **7** from *D*‐mannose complements the previously described synthesis of intermediate **16**, enabling access to the spiroketal core framework found in various natural products such as Cephalosporolide F. Despite difficulties in chromatographic separation of diastereoisomeric mixtures, the route taken enabled the preparation of *N*‐alkoxyphthalimide **7** in 23% overall yield from *D*‐mannose. The stereoselective synthesis of Cephalosporolide F was achieved under electrochemical conditions, while the concomitant formation of Cephalosporolide E suggests that epimerization likely occurs during the purification stage. Although photocatalytic redox conditions involving iridium afforded slightly higher yields, the electrochemical alternative provided comparable reactivity in shorter reaction times, while avoiding the use of scarce and costly metal catalysts. Both methodologies circumvent toxic stannyl reagents, reinforcing the value of electrochemistry as a sustainable strategy, even when considering the use of CH_2_Cl_2_ and tetrabutylammonium salts in the ES protocol.

## Supporting Information

The authors have cited additional references within the Supporting Information.^[^ ^45^, ^46^, ^47^, ^48^ ^]^

## Conflict of Interest

The authors declare no conflict of interest.

## Supporting information

Supplementary Material

## Data Availability

The data that support the findings of this study are available in the supplementary material of this article.
