# Supplementary material for: Electrochemical and Photoredox Catalysis for Constructing 5,5‐Spirocycles via Reductive Activation of N‐alkoxyphthalimides for the Total Synthesis of (−)‐Cephalosporolide F
Source: ChemSusChem. 2025 Sep 15;18(20):e202501605. doi: 10.1002/cssc.202501605 (PMC12548939; doi:10.1002/cssc.202501605)
Supplement: Supplementary file 1 — Supplementary Material [file CSSC-18-e202501605-s001.pdf]

Electrochemical and Photoredox Catalysis Approaches in Assembling 5,5-Spirocycles via Reductive Activation of N-Alkoxyphthalimides. Implementation in the Total Synthesis of (-)-Cephalosporolide F

Julio Romero-Ibañez,[1,2] Karen A. Guarneros-Cruz,[3] Fernando Sartillo-Piscil,[3,*] Bernardo A. Frontana-Uribe[1,2,*]

[1]Centro Conjunto de Investigaciones en Química Sustentable UAEMéx-UNAM, Estado de México, México.

[2]Instituto de Química, Universidad Nacional Autónoma de México, Ciudad de México, México.

[3]Facultad de Ciencias Químicas, Benemérita Universidad Autónoma de Puebla, Puebla, México.

*Corresponding author

*To whom correspondence should be addressed

(fernando.sartillo@correo.buap.mx; bafrontu@unam.mx).

Supporting Information

Table of contents

General considerations S3
Experimental section S4
Synthesis of N-alkoxyphthalimides 2a-2e S4
Synthesis of N-alkoxyphthalimide 4 .... S9
Synthesis of Spirocycles 3 and 5 .... S13
General electrochemical procedure S13
General photocatalytic procedure S14
Synthesis of Cephalosporolide F S16
Calculation of time and charge per mmol S25
Cyclic voltammograms S27
Cyclic voltammogram of N-alkoxyphthalimide 2a S27
Cyclic voltammograms of N-alkoxyphthalimides 2a-2e S27
Cyclic voltammogram of N-alkoxyphthalimide 4 .... S28
NMR Spectra S29
HRMS Spectra S97

General considerations

Commercially available reagents were used without further purification. Unless otherwise noted, reactions were carried out under an inert argon atmosphere with dry solvents under anhydrous conditions. Solvents were used as technical grade, and freshly distilled prior to use. Column chromatography (CC) was performed using silica gel (70-230 mesh) with solvents indicated in the text. Melting points were not corrected and carried out on a Fisher-Scientific 12-144 melting point apparatus. NMR spectra were recorded on Bruker (300 and 400 MHz, Avance III) and Bruker (500 MHz, Ascend) using TMS as an internal reference for ^{1}H (0.0 ppm) and $CDCl_3$ for ^{1}H (7.26 ppm), and ^{13}C (77.16 ppm); chemical shifts (δ) are stated in parts per million (ppm) and Hz for the coupling constants (J). The following abbreviations (or combinations thereof) were used to explain the multiplicities: s = singlet, d = doublet, t = triplet, q = quartet, m = multiplet, br = broadened.

Voltammetric studies were carried out using a PGSTAT204 Potentiostat and a conventional glass cell of 10 mL. Reference electrode: Ag/Ag^{+} (filled with $AgNO_3$ 0.01 M in CH_3CN). Working electrode: glassy carbon disk (diameter: 3mm). Counter electrode: platinum wire (99.95% purity).

Preparative electrolysis was carried out using an ElectraSyn 2.0 in a 5 or 10-mL glass cell. Electrodes: Glassy carbon (GC). Dimensions of commercial electrodes: 8 x 52.5 x 2 mm. Electrode spacing: 5 mm. Voltage limit: 30V.

Note: The exact waveform could not be verified from the instrument technical data or programming. Based on reports from Baran's group, rAP is classified as a bipolar square wave, with either current or potential being controlled.[1,2] For rAP, the duty cycle is 50%, For "pulsed alternating polarity" experiment, the duty cycle varies according to the "pol time" setting.

Photoredox reactions were carried out using an EvoluChem PhotoRedOx Box reactor and EvoluChem LED 450PF non-dimmable 110V-220V (PF series light 18W; 328 mW/cm^2).

---

[1] Y. Kawamata, K. Hayashi, E. Carlson, S. Shaji, D. Waldmann, B. J. Simmons, J. T. Edwards, C. W Zapf, M. Saito, P. S. Baran, "Chemoselective Electrosynthesis Using Rapid Alternating Polarity" *J. Am. Chem. Soc.* **2021**, *143*, 16580-16588.

[2] Y. Kawamata, P. S. Baran, "Rapid Alternating Polarity as a Unique Tool for Synthetic Electrochemistry" *J. Synth. Org. Chem., Jpn*. **2023**, *81*, 1020-1027.

Experimental section

*Synthesis of N-alkoxyphthalimides **2a-2e***

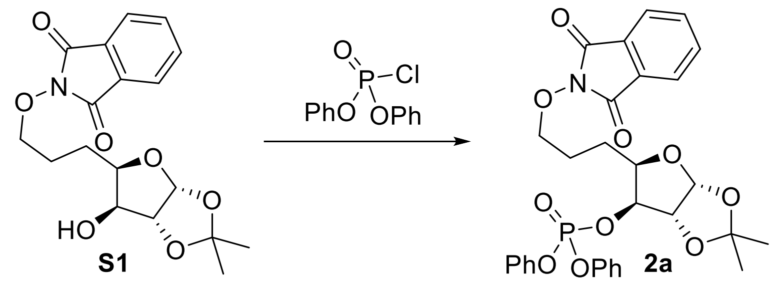

(3aR,5R,6S,6aR)-5-(3-((1,3-dioxoisoindolin-2-yl)oxy)propyl)-2,2-dimethyltetrahydro-furo[2,3-d]-[1,3]dioxol-6-yl diphenyl phosphate (2a): To a mixture of alcohol **S1**[3] (1 g, 2.8 mmol) and DMAP (1.02 g, 8.42 mmol) in dry THF (56 mL) under an inert atmosphere at 0 °C NEt_3 (1.17 mL, 8.42 mmol), and $(PhO)_2POCl$ (1.75 mL, 8.42 mmol) were added dropwise. The reaction was allowed to warm at room temperature and was stirred for 3 h before adding a saturated aqueous NH_4Cl solution (10 mL). Then, the solvent was removed under reduced pressure, followed by extraction with CH_2Cl_2 (3 × 15 mL). The combined organic phases were dried with Na_2SO_4, and the solvent was removed under reduced pressure. The crude reaction was purified by column chromatography [SiO_2, hexanes/EtOAc 5:5] to give 1.5 g (90%) of **2a**.

2a: White solid, m.p.: 61-63 °C. $[\alpha]_D$ = - 12.1 (c = 2.0, $CHCl_3$). ^{1}H NMR (300 MHz, $CDCl_3$) δ: 7.83-7.79 (m, 2H), 7.76-7.72 (m, 2H), 7.39-7.34 (m, 4H), 7.28-7.17 (m, 6H), 5.84 (d, J = 3.9 Hz, 1H), 4.90 (dd, J = 7.5, 2.4 Hz, 1H), 4.65 (d, J = 3.9 Hz, 1H), 4.35-4.28 (m, 1H), 4.16-4.09 (m, 2H), 1.74-1.95 (m, 4H), 1.50 (s, 3H), 1.27 (s, 3H); ^{13}C NMR (75 MHz, $CDCl_3$) δ:

[3] F. Sartillo-Piscil, M. Vargas, C. A. de Parrodi, L. Quintero, "Diastereoselective synthesis of 1,2-O-isopropylidene-1,6-dioxaspiro[4,4]nonane applying the methodology of generation of radical cations under non-oxidizing conditions" *Tet. Lett.* **2003**, *44*, 3919-3921.

163.5(2C), 150.3 (dd, J = 7.5, 1.5 Hz, 2C), 134.5(2C), 129.9(4C), 128.9(2C), 125.7(2C), 123.5(2C), 120.2(dd, J = 6.0, 5.3 Hz, 4C), 112.0, 104.3, 83.5(d, J = 2.3 Hz), 81.9(d, J = 6.0 Hz), 79.0(d, J = 8.3 Hz), 77.8, 26.5, 26.2, 24.8, 24.2. $^{31}P\{^1H\}$ NMR (202 MHz, $CDCl_3$) δ: -12.32. HRMS (DART-TOF) *m/z:* $[M+H]^+$ calcd for $C_{30}H_{31}NO_{10}P$, 596.16801; found, 596.16850.

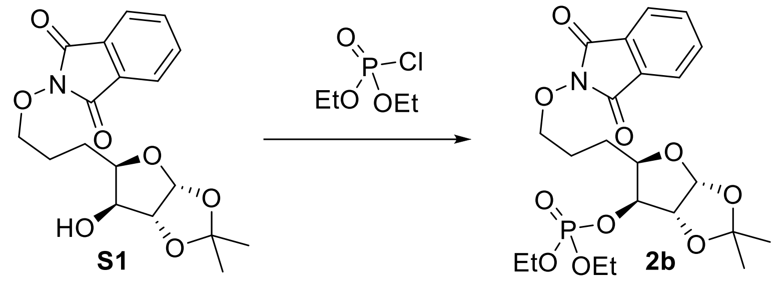

(3aR,5R,6S,6aR)-5-(3-((1,3-dioxoisoindolin-2-yl)oxy)propyl)-2,2-dimethyltetrahydro-furo[2,3-d][1,3]dioxol-6-yl diethyl phosphate (2b): To a mixture of alcohol **S1**[3] (0.22 g, 0.6 mmol) and DMAP (0.18 g, 0.18 mmol) in dry THF (12 mL) under an inert atmosphere at 0 °C NEt_3 (0.25 mL, 1.81 mmol), and $(EtO)_2POCl$ (0.25 mL, 1.81 mmol) were added dropwise. The reaction was allowed to warm at room temperature and was stirred for 3 h before adding a saturated aqueous NH_4Cl solution (5 mL). Then, the solvent was removed under reduced pressure, followed by extraction with CH_2Cl_2 (3 × 10 mL). The combined organic phases were dried with Na_2SO_4, and the solvent was removed under reduced pressure. The crude reaction was purified by column chromatography [SiO_2, hexanes/EtOAc 5:5] to give 0.226 g (75%) of **2b**.

2b:[3] Colorless oil. $[\alpha]_D$ = – 15.3 (c = 3.0, $CHCl_3$). ^1H NMR (300 MHz, $CDCl_3$) δ: 7.84-7.83 (m, 2H), 7.77-7.75 (m, 2H), 5.92 (d, J = 2.4 Hz, 1H), 4.76 (d, J = 2.4 Hz, 1H), 4.67 (dd, J = 4.8, 1.5 Hz, 1H), 4.32-4.23 (m, 3H), 4.22-4.13 (m, 4H), 2.00-1.90 (m, 4H), 1.51 (s, 3H), 1.39-1.35 (m, 6H), 1.32 (s, 3H); ^{13}C NMR (75 MHz, $CDCl_3$) δ: 163.6(2C), 134.5(2C), 129.0(2C), 123.5(2C), 111.9, 104.5, 83.8 (d, J = 1.5 Hz), 80.3 (d, J = 3.8 Hz), 79.2 (d, J = 4.5 Hz), 78.0, 64.4 (t,

J = 3.8 Hz, 2C), 26.7, 26.3, 24.9, 24.4, 16.2 (dd, J = 3.8, 2.3 Hz, 2C). HRMS (DART-TOF) *m/z:* $[M+H]^+$ calcd for $C_{22}H_{31}NO_{10}P$, 500.16801; found, 500.16793.

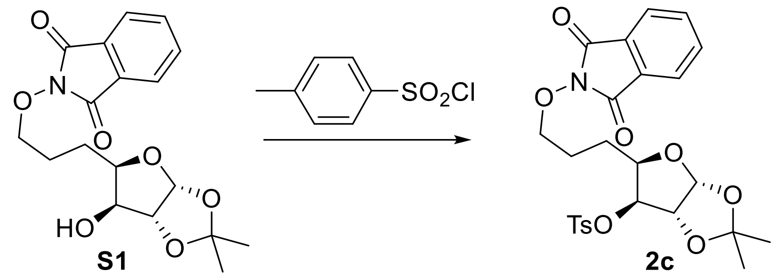

(3aR,5R,6S,6aR)-5-(3-((1,3-dioxoisoindolin-2-yl)oxy)propyl)-2,2-dimethyltetrahydro-furo[2,3-d][1,3]dioxol-6-yl 4-methylbenzenesulfonate (2c): To a mixture of alcohol **S1**[3] (0.2 g, 0.55 mmol), TsCl (0.31 g, 1.65 mmol), and DMAP (13 mg, 0.11 mmol) in dry CH_2Cl_2 (5.5 mL) under an inert atmosphere at 0 °C NEt_3 (0.23 mL, 1.65 mmol) was added dropwise. The reaction was allowed to warm at room temperature and was stirred for 12 h before adding a saturated aqueous NH_4Cl solution (5 mL). The phases were separated, and the aqueous phase was extracted with CH_2Cl_2 (3 × 10 mL). The combined organic phases were dried with Na_2SO_4, and the solvent was removed under reduced pressure. The crude reaction was purified by column chromatography [SiO_2, hexanes/EtOAc 5:5] to give 0.199 g (70%) of **2c**.

2c: White solid, m.p.: 143-145 ºC. $[\alpha]_D$ = – 12.3 (c = 2.0, $CHCl_3$). ^{1}H NMR (300 MHz, $CDCl_3$) δ: 7.86-7.83 (m, 4H), 7.77-7.74 (m, 2H), 7.40 (d, J = 7.8 Hz, 2H), 5.89 (d, J = 3.9 Hz, 1H), 4,72 (t, J = 3.9 Hz, 2H), 4.25 (ddd, J = 7.8, 5.1, 2.7 Hz, 1H), 4.21-4.07 (m, 2H), 2.45 (s, 3H), 1.87-1.76 (m, 2H), 1.67-1.60 (m, 2H), 1.48 (s, 3H), 1.29 (s, 3H); ^{13}C NMR (75 MHz, $CDCl_3$) δ: 163.6(2C), 145.6, 134.6(2C), 133.0, 130.3(2C), 129.1(2C), 128.2(2C), 123.6(2C), 112.2, 104.5, 83.6, 82.9, 78.5, 77.9, 26.6, 26.3, 24.8, 24.3, 21.8. HRMS (DART-TOF) *m/z:* $[M+H]^+$ calcd for $C_{25}H_{28}NO_9S$, 518.14793; found, 518.14807.

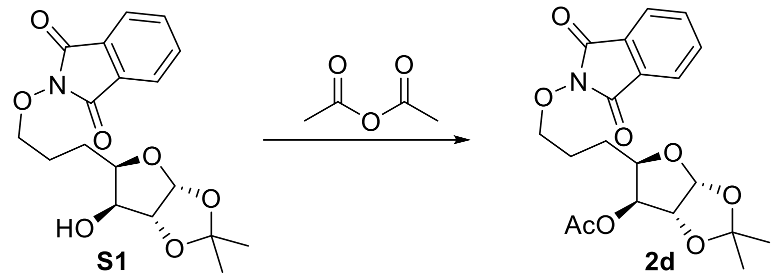

(3aR,5R,6S,6aR)-5-(3-((1,3-dioxoisoindolin-2-yl)oxy)propyl)-2,2-dimethyltetrahydro-furo[2,3-d][1,3]dioxol-6-yl acetate (2d): To a mixture of alcohol **S1**[3] (0.16 g, 0.44 mmol), and DMAP (5.4 mg, 0.044 mmol) in dry CH_2Cl_2 (4.4 mL) under an inert atmosphere at 0 °C NEt_3 (0.18 mL, 1.32 mmol) was added dropwise, followed by acetic anhydride (0.12 mL, 1.32 mmol). The reaction was allowed to warm at room temperature and was stirred for 2 h before adding H_2O (5 mL). The phases were separated, and the aqueous phase was extracted with CH_2Cl_2 (3 × 5 mL). The combined organic phases were dried with Na_2SO_4, and the solvent was removed under reduced pressure. The crude reaction was purified by column chromatography [SiO_2, hexanes/EtOAc 5:5] to give 142.7 mg (80%) of **2d**.

2d: White solid, m.p.: 153-154 ºC. $[\alpha]_D$ = – 10.4 (*c* = 2.2, $CHCl_3$). ^{1}H NMR (500 MHz, $CDCl_3$) δ: 7.84-7.80 (m, 2H), 7.75-7.72 (m, 2H), 5.88 (d, *J* = 4.0 Hz, 1H), 5.15 (d, *J* = 2.5 Hz, 1H), 4.50 (d, *J* = 4.0 Hz, 1H), 4.32 (td, *J* = 6.5, 3.0 Hz, 1H), 4.26-4.19 (m, 2H), 2.11 (s, 3H), 1.99-1.91 (m, 1H), 1.87-1.81 (m, 3H), 1.51 (s, 3H), 1.29 (s, 3H); ^{13}C NMR (125 MHz, $CDCl_3$) δ: 170.1, 163.7(2C), 134.6(2C), 129.1(2C), 123.6(2C), 111.9, 104.5, 83.7, 78.8, 78.1, 77.0, 26.7, 26.3, 25.2, 24.5, 20.9. HRMS (ESI-TOF) *m/z:* $[M+H]^+$ calcd for $C_{20}H_{24}NO_8$, 406.15019; found, 406.15132.

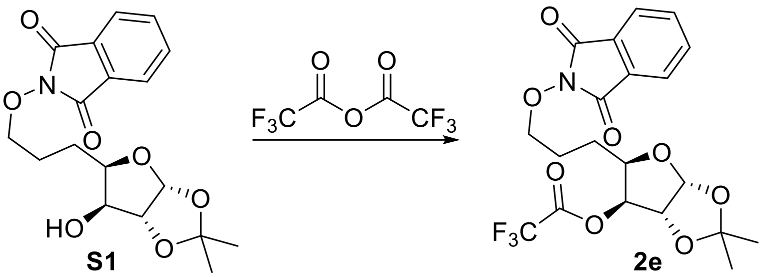

(3aR,5R,6S,6aR)-5-(3-((1,3-dioxoisoindolin-2-yl)oxy)propyl)-2,2-dimethyltetrahydro-furo[2,3-d][1,3]dioxol-6-yl 2,2,2-trifluoroacetate (2e): To a mixture of alcohol **S1**[3] (0.25 g, 0.688 mmol), and DMAP (8.5 mg, 0.069 mmol) in dry CH_2Cl_2 (6.9 mL) under an inert atmosphere at 0 °C, NEt_3 (0.29 mL, 2.06 mmol) was added dropwise, followed by trifluoroacetic anhydride (0.29 mL, 2.06 mmol). The reaction was allowed to warm at room temperature and was stirred for 2 h before adding H_2O (7 mL). The phases were separated, and the aqueous phase was extracted with CH_2Cl_2 (3 × 10 mL). The combined organic phases were dried with Na_2SO_4, and the solvent was removed under reduced pressure. The crude reaction was purified by column chromatography [SiO_2, CH_2Cl_2/EtOAc 97:3] to give 158 mg (50%) of **2e**.

2e: Colorless oil. $[\alpha]_D$ = – 15.9 (c = 1.5, $CHCl_3$). ^{1}H NMR (300 MHz, $CDCl_3$) δ: 7.85-7.81 (m, 2H), 7.79-7.75 (m, 2H), 5.97 (d, J = 3.9 Hz, 1H), 5.35 (d, J = 2.7 Hz, 1H), 4.61 (d, J = 3.9 Hz, 1H), 4.49-4.44 (m, 1H), 4.31-4.19 (m, 2H), 2.02-1.83 (m, 4H), 1.55 (s, 3H), 1.33 (s, 3H); ^{13}C NMR (75 MHz, $CDCl_3$) δ: 163.6(2C), 156.7 (q, J = 42.8 Hz), 134.5(2C), 129.0(2C), 123.6(2C), 114.4 (q, J = 284.3 Hz), 112.3, 104.4, 83.1, 80.7, 78.1, 77.7, 26.5, 26.2, 25.0, 24.0. ^{19}F NMR (282 MHz, $CDCl_3$) δ: -74.75. HRMS (DART-TOF) *m/z:* $[M-C3H6O]^+$ calcd for $C_{15}H_{14}NO_5$, 288.08665; found, 288.08637.

Synthesis of N-alkoxyphthalimide ***4***

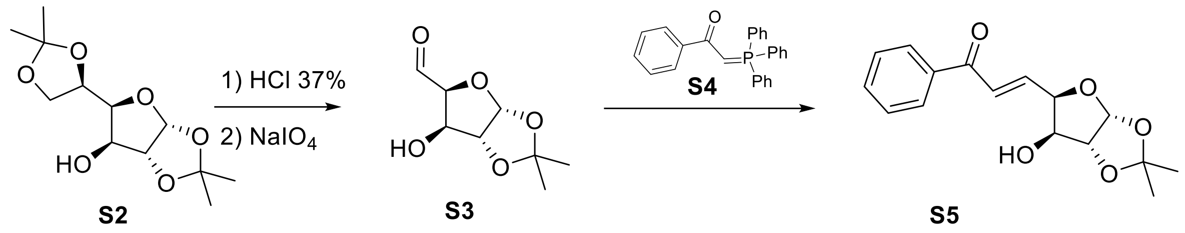

3-((3aR,5R,6S,6aR)-6-hydroxy-2,2-dimethyltetrahydrofuro[2,3-d][1,3]dioxol-5-yl)-1-phenylprop-2-en-1-one (S5): To a solution of diacetone-D-glucose **S2** (2 g, 7.68 mmol) in a mixture of EtOH:H_2O (7:3; 40 mL), 1 mL of HCl (37%) was added at 0 ºC. Then, the reaction mixture was stirred at r.t. Upon completion, Na_2CO_3 was added until the mixture reached a pH of 7. Then, $NaIO_4$ (1.64 g, 7.68 mmol) was slowly added. After 30 min, the mixture solution was filtered through a sintered funnel and rinsed with EtOH (5 × 10 mL). The organic solvent was removed under reduced pressure, and the aqueous phase was extracted with EtOAc (3 × 15 mL). The combined organic phases were dried with Na_2SO_4, and the solvent was removed under reduced pressure to obtain **S3**, which was submitted to the next reaction without previous purification. To a solution of the phosphorus ylide **S4**[4] (3.5 g, 9.2 mmol) in THF (26 mL) was added a solution of **S3** in THF (12 mL) at 0 °C. Then, the reaction mixture was stirred at room temperature for 12 h. Next, the reaction was cooled to 0 °C and quenched with 20 mL of H_2O. The organic solvent was evaporated under reduced pressure, and the aqueous phase was extracted with EtOAc (4 × 20 mL). The combined organic phases were dried with Na_2SO_4, and the solvent was removed under reduced pressure. The crude reaction was purified by column chromatography [SiO_2, hexanes/EtOAc 7:3, then 5:5] to give 1.11 g (50%) of **S5**.

[4] S.-Y. Luo, G.-Q. Lin, Z.-T. He, "Asymmetric Copper-Catalyzed Alkynylallyclic Dimethylamination" *Org. Chem. Front.* **2024**, *11*, 690-695.

S5: Light yellow oil. ^{1}H NMR (300 MHz, $CDCl_3$) δ: 7.96-7.90 (m, 2H), 7.58-7.52 (m, 1H), 7.48-7.41 (m, 2H), 7.34 (dd, *J* = 15.6, 2.1 Hz, 1H), 7.01 (dd, *J* = 15.6, 3.9 Hz, 1H), 6.03 (d, *J* = 3.6 Hz, 1H), 4.96-4.93 (m, 1H), 4.59 (d, *J* = 3.9 Hz, 1H), 4.34 (d, *J* = 3.0 Hz, 1H), 1.51 (s, 3H), 1.32 (s, 3H); ^{13}C NMR (75 MHz, $CDCl_3$) δ: 190.7, 141.2, 137.1, 133.4, 128.9(2C), 128.7(2C), 127.4, 112.1, 104.9, 85.1, 80.1, 76.1, 26.9, 26.2. HRMS (DART-TOF) *m/z:* $[M+H]^+$ calcd for $C_{16}H_{19}O_5$, 291.12270; found, 291.12235.

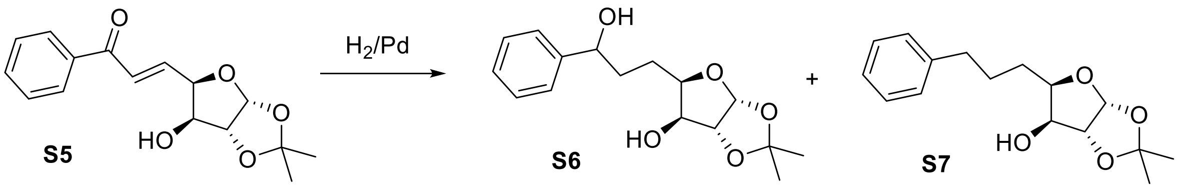

(3aR,5R,6S,6aR)-5-(3-hydroxy-3-phenylpropyl)-2,2-dimethyltetrahydrofuro[2,3-d][1,3]dioxol-6-ol (S6): A mixture of **S5** (1.1 g, 3.78 mmol), and Pd/C (10 wt. %, 0.11 g) in EtOAc (15 mL) was stirred under H_2 atmosphere (one balloon) for 2 h. After this time, the reaction was filtered through a Celite pad and rinsed with EtOAc. The solvent was removed under reduced pressure. The residue was purified by column chromatography [SiO2, hexanes/EtOAc, 7:3, then 1:1] to give 0.16 g (15%) of **S7**, and 0.78 g (70%) of **S6**.

S6: Colorless oil. Reported as a diastereoisomeric mixture 1:1. ^{1}H NMR (500 MHz, $CDCl_3$) δ: 7.30-7.26 (m, 8H), 7.24-7.20 (m, 2H), 5.77 (d, *J* = 4.0 Hz, 2H), 4.66 (dd, *J* = 7.5, 4.0 Hz, 1H), 4.61 (dd, *J* = 8.5, 3.5 Hz, 1H), 4.40 (d, *J* = 4.5 Hz, 2H), 4.04-3.98 (m, 4H), 1.89-1.73 (m, 6H), 1.70-1.62 (m, 2H), 1.43 (s, 3H), 1.42 (s, 3H), 1.24 (s, 6H); ^{13}C NMR (125 MHz, $CDCl_3$) δ: 144.4, 144.2, 128.4(2C), 128.3(2C), 127.5, 127.4, 125.8(2C), 125.7(2C), 111.2(2C), 104.3, 104.1, 85.2, 85.0, 80.7, 80.6, 75.2, 74.6, 74.3, 73.7, 35.0(2C), 26.6, 26.5, 26.1, 26.0, 24.3, 23.6. HRMS (DART-TOF) *m/z:* $[M-H]^+$ calcd for $C_{16}H_{21}O_5$, 293.13835; found, 293.13816; $[M-OH]^+$ calcd for $C_{16}H_{21}O_4$, 277.14344; found, 277.14337.

S7: White solid; m.p.: 100-101 ºC. $[\alpha]_D$ = – 18.1 (*c* = 3.0, $CHCl_3$). ^{1}H NMR (500 MHz, $CDCl_3$) δ: 7.26-7.23 (m, 2H), 7.17-7.13 (m, 3H), 5.84 (d, *J* = 4.0 Hz, 1H), 4.44 (d, *J* = 4.0 Hz, 1H), 4.10-4.06 (m, 1H), 3.98 (br s, 1H), 2.69-2.63 (m, 2H), 1.80-1.59 (m, 4H), 1.46 (s, 3H), 1.27 (s, 3H); ^{13}C NMR (125 MHz, $CDCl_3$) δ: 142.0, 128.3(4C), 125.8, 111.2, 104.2, 85.3, 80.3, 75.1, 35.8, 27.7, 27.2, 26.5, 26.1. HRMS (DART-TOF) *m/z:* $[M+H]^+$ calcd for $C_{16}H_{23}O_4$, 279.15909; found, 279.15876.

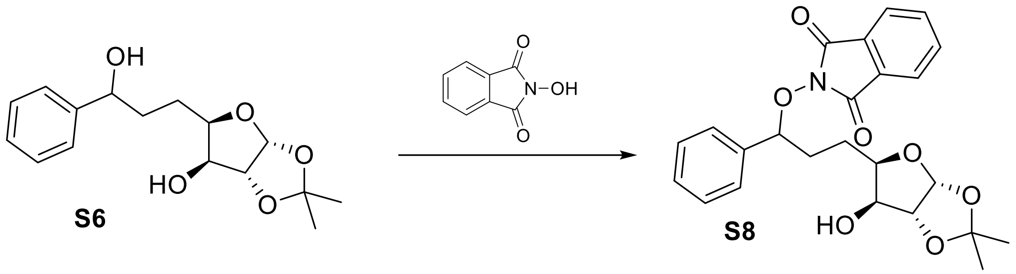

2-(3-((3aR,5R,6S,6aR)-6-hydroxy-2,2-dimethyltetrahydrofuro[2,3-d][1,3]dioxol-5-yl)-1-phenylpropoxy)isoindoline-1,3-dione (S8): To a mixture of **S6** (0.3 g, 1.02 mmol), PPh_3 (0.32 g, 1.22 mmol) and *N*-hydroxyphthalimide (0.2 g, 1.22 mmol) in THF (10.2 mL), DIAD (0.26 mL, 1.33 mmol)was added at 0 °C. After 15 minutes, the reaction mixture was stirred at room temperature for 3 h. Next, the solvent was removed under reduced pressure, and the residue was purified by column chromatography [SiO_2, hexanes/EtOAc 7:3, then 5:5] to give 0.32 g (72%) of **S8**.

S8: White solid. Reported as a diastereoisomeric mixture ~1:1. ^{1}H NMR (300 MHz, $CDCl_3$) δ: 7.74-7.65 (m, 8H), 7.47-7.43 (m, 4H), 7.33-7.27 (m, 6H), 5.89 (d, *J* = 3.9 Hz, 2H), 5.36-5.30 (m, 2H), 4.53 (d, *J* = 3.9 Hz, 2H), 4.21-4.11 (m, 4H), 2.54 (br, 1OH), 2.46 (br, 1OH), 2.41-2.20 (m, 2H), 2.15-1.78 (m, 6H), 1.49 (s, 3H), 1.48 (s, 3H), 1.31 (s, 6H); ^{13}C NMR (75 MHz, $CDCl_3$) δ: 164.0(2C), 163.9(2C), 138.0, 137.8, 134.5(2C), 134.4(2C), 129.2(2C), 129.1(2C), 128.9, 128.8, 128.5(2C), 128.4(2C), 128.1(2C), 127.9(2C), 123.5(4C),

111.5(2C), 104.4(2C), 89.7, 88.8, 85.5, 85.4, 80.5, 80.3, 75.4, 75.3, 31.9, 31.5, 26.7(2C), 26.3(2C), 24.5, 23.6. HRMS (DART-TOF) *m/z:* $[M+H]^+$ calcd for $C_{24}H_{26}NO_7$, 440.17038; found, 440.17070.

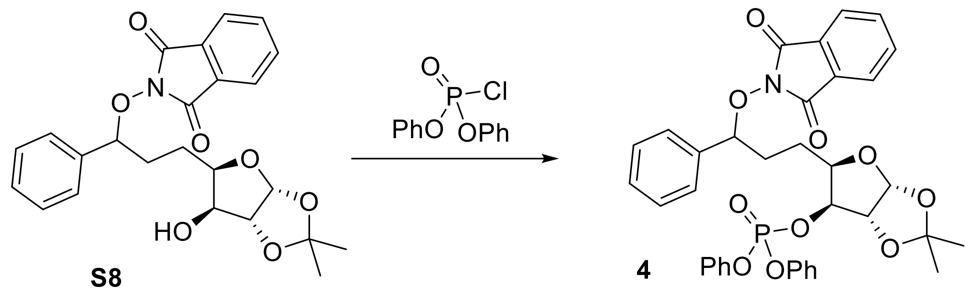

(3aR,5R,6S,6aR)-5-(3-((1,3-dioxoisoindolin-2-yl)oxy)-3-phenylpropyl)-2,2-dimethyl-tetrahydrofuro[2,3-d][1,3]dioxol-6-yl diphenyl phosphate (4): Employing the same procedure as for **2a**, **S8** (0.3 g, 0.68 mmol) was used to obtain **4**. The residue was purified by column chromatography [SiO_2, hexanes/EtOAc 7:3, then 5:5] to give 298 mg (65%) of **4**.

4: Colorless oil. Reported as a diastereoisomeric mixture 1:0.8. ^1H NMR (300 MHz, $CDCl_3$) δ: 7.71-7.61 (m, 7.2H), 7.43-7.17(m, 27H), 5.83 (d, *J* = 3.9 Hz, 0.8H), 5.81 (d, *J* = 3.9 Hz, 1H), 5.28 (apparent q, *J* = 6.9 Hz, 1.8H), 4.86 (apparent dt, *J* = 7.8, 2.7 Hz, 1.8H), 4.64 (d, *J* = 3.9 Hz, 1.8H), 4.38-4.31 (m, 0.8H), 4.28-4.22 (m, 1H), 2.39-2.18 (m, 1.8H), 2.13-1.66 (m, 5.4H), 1.50 (s, 2.4H), 1.47 (s, 3H), 1.26 (s, 2.4H), 1.25 (s, 3H); ^{13}C NMR (75 MHz, $CDCl_3$) δ: 163.6(4C), 150.4 (d, *J* = 7.5 Hz, 2C), 150.3 (d, *J* = 6.8 Hz, 2C), 137.8, 137.7, 134.3(2C), 134.2(2C), 129.9(8C), 129.1(4C), 128.9(2), 128.4(2C), 128.3(2C), 128.0(4C), 125.7(2C), 125.6(2C), 123.3(4C), 120.2 (apparent d, *J* = 5.3 Hz, 4C), 120.1 (apparent dd, *J* = 5.3, 3.0 Hz, 4C), 112.0(2C), 104.4, 104.3, 88.9, 88.4, 83.6(2C), 82.0 (d, *J* = 6.8 Hz), 81.7 (d, *J* = 6.0 Hz), 79.3 (d, *J* = 7.5 Hz), 78.9 (d, *J* = 7.5 Hz), 31.5, 31.4, 26.6(2C), 26.2(2C), 24.4, 24.3. $^{31}P\{^1H\}$ NMR (202 MHz, $CDCl_3$) δ: -12.34, -12.38. HRMS (DART-TOF) *m/z:* $[M+H]^+$ calcd for $C_{36}H_{35}NO_{10}P$, 672.19931; found, 672.19960.

*Synthesis of Spirocycles **3** and **5***

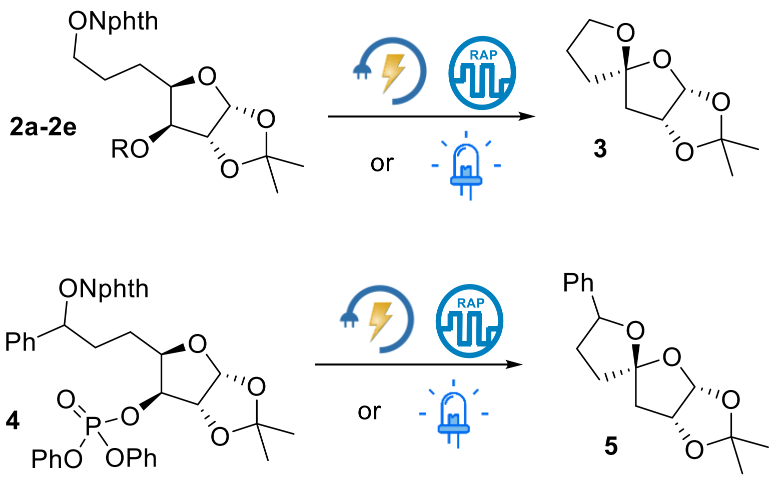

General electrochemical procedure

In an ElectraSyn 10 mL glass-vial with a stir-bar was put nBu_4NPF_6 (95%; 1 mmol, 0.1 M), Hantzsch ester (0.4 mmol), and *N*-alkoxyphthalimide **2** or **4** (0.2 mmol). The IKA cell cap equipped with two standard glassy carbon electrodes was laid. Then, 10 mL of dry dichloromethane was added (0.5 mL extra to compensate for evaporation), and the solution was stirred and degassed with nitrogen for 5 minutes. The reaction was performed with the following experiment programming: rapid alternating polarity, 25 ms (20 Hz), current = 17.1 mA (immersion depth: 3.4 cm; geometric area = 2.72 cm^2; current density = 6.3 mA cm^2), 12 F/mol, and 1000 rpm. Upon completion, the solvent was removed under reduced pressure. Then, the electrolyte was crystallized with Et_2O, and the solids were filtered and washed with Et_2O (3 × 10 mL). The ethereal phase was evaporated, and the residue was purified by column chromatography.

Note: No temperature rise was detected at the end of the experiment.

General photocatalytic procedure

In a 20 mL glass-vial with a stir-bar was put [Ir(dtbbpy)(ppy)$_2$]PF_6 (2 mol%; 0.004 mmol), Hantzsch ester (0.4 mmol), and *N*-alkoxyphthalimide **2** or **4** (0.2 mmol). The vial was stopped with a septum and placed under an inert atmosphere. Then, 10 mL of dry dichloromethane was added (0.5 mL extra to compensate for evaporation), and the solution was stirred and degassed with nitrogen for 5 minutes. Then, the vial was set in the photoreactor, stirred, and irradiated with a 450nm blue-LED lamp for 3 h at 0 ºC. Upon completion, H_2O (5 mL) was added. The phases were separated, and the aqueous phase was extracted with CH_2Cl_2 (3 × 10 mL). The combined organic phases were dried with Na_2SO_4, and the solvent was removed under reduced pressure. The crude reaction was purified by column chromatography

3: Purification [SiO_2, Hexanes/EtOAc 8:2]. Colorless oil. $[\alpha]_D$ = + 21.5 (*c* = 0.7, $CHCl_3$). ^1H NMR (400 MHz, $CDCl_3$) δ: 5.81 (d, *J* = 4.0 Hz, 1H), 4.78 (ddd, *J* = 6.0, 4.0, 1.6 Hz, 1H), 3.96 (td, *J* = 8.0, 4.8 Hz, 1H), 3.88-3.83 (m, 1H), 2.37 (dd, *J* = 14.4, 6.0 Hz, 1H), 2.31-2.25 (m, 2H), 2.16-2.09 (m, 1H), 1.97-1.91 (m, 2H), 1.54 (s, 3H), 1.34 (s, 3H); ^{13}C NMR (75 MHz, $CDCl_3$) δ: 115.8, 112.2, 104.6, 80.1, 67.8, 41.3, 37.2, 27.5, 26.7, 24.5. HRMS (DART-TOF) *m/z:* $[M-H]^+$ calcd for $C_{10}H_{15}O_4$, 199.09649; found, 199.09615; $[M-C_3H_6O]^+$ calcd for $C_7H_{11}O_3$, 143.07027; found, 143.07094.

5a: Purification [SiO_2, DCM/EtOAc 96:4]. Colorless oil. $[\alpha]_D$ = + 27.8 (*c* = 0.5, $CHCl_3$). ^1H NMR (400 MHz, $CDCl_3$) δ: 7.46-7.43 (m, 2H), 7.35-7.30 (m, 2H), 7.28-7.23 (m, 1H), 5.94 (d, *J* = 4.0 Hz, 1H), 4.97 (dd, *J* = 9.6, 6.0 Hz, 1H), 4.81 (ddd, *J* = 6.0, 4.0, 1.6 Hz, 1H), 2.49-2.42 (m, 2H), 2.37-2.31 (m, 2H), 2.24-2.06 (m, 2H), 1.57 (s, 3H), 1.36 (s, 3H); ^{13}C NMR (75 MHz, $CDCl_3$) δ: 142.8, 128.6(2C), 127.8, 126.7(2C), 116.1, 112.2, 104.6, 82.7, 80.0, 41.6, 39.0, 34.7, 27.5, 26.7. HRMS (DART-TOF) *m/z:* $[M-H]^+$ calcd for $C_{16}H_{19}O_4$, 275.12779; found, 275.12781; $[M-C_3H_6O]^+$ calcd for $C_{13}H_{15}O_3$, 219.10157; found, 219.10149.

5b: Purification [SiO_2, DCM/EtOAc 96:4]. Obtained and reported with traces of **5a**. Light yellow oil. **5b** chemical shifts: ^{1}H NMR (400 MHz, $CDCl_3$) δ: 7.35-7.25 (m, 5H), 5.87 (d, *J* = 4.0 Hz, 1H), 5.15 (t, *J* = 7.0 Hz, 1H), 4.83 (ddd, *J* = 6.0, 4.0, 2.0 Hz, 1H), 2.58-2.49 (m, 2H), 2.44-2.31 (m, 2H), 2.24-2.16 (m, 1H), 1.87 (dddd, *J* =12.2, 9.4, 6.4, 5.2 Hz, 1H), 1.56 (s, 3H), 1.37 (s, 3H); ^{13}C NMR (100 MHz, $CDCl_3$) δ: 142.5, 128.5(2C), 127.6, 125.8(2C), 116.2, 112.6, 105.0, 80.3, 80.2, 41.9, 37.1, 33.5, 27.7, 26.9. HRMS (DART-TOF) *m/z:* $[M-H]^+$ calcd for $C_{16}H_{19}O_4$, 275.12779; found, 275.12791; $[M-C_3H_6O]^+$ calcd for $C_{13}H_{15}O_3$, 219.10157; found, 219.10144.

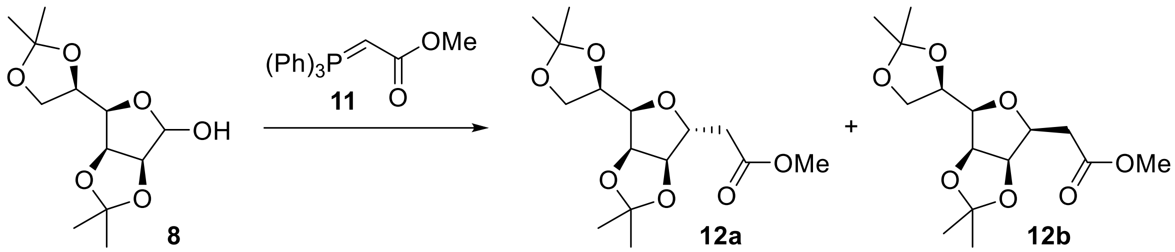

Methyl 2-((3aR,6R,6aS)-6-((R)-2,2-dimethyl-1,3-dioxolan-4-yl)-2,2-dimethyltetra-hydrofuro[3,4-d][1,3]dioxol-4-yl)acetate (12): A solution of **8**[5] (1.0 g, 3.84 mmol), and **11** (2.57 g, 7.68 mmol) in CH_3CN (48 mL) was stirred under reflux (85 °C) for 16 h. Next, the solvent was removed under reduced pressure, and the residue was purified by column chromatography [SiO_2, hexanes/EtOAc 8:2] to give 547 mg (45%) of **12b**, and 522 mg (43%) of **12a**.[6]

Epimerization of ***12a****:* To a solution of alcohol **12a** (0.5 g, 1.58 mmol) in anhydrous Et_2O (79 mL) at 0 °C, NaHMDS (1M; 2.37 mL) was added dropwise. After 10 minutes, the reaction was stirred for 12 h at r.t. Then, H_2O (20 mL) was added at 0 ºC. The phases were separated, and the organic phase was washed with Brine (2 × 30 mL). The combined organic phases were dried with Na_2SO_4, and the solvent was removed under reduced pressure. The crude reaction was purified by column chromatography [SiO_2, hexanes/EtOAc 8:2] to give 430 mg (86%) of **12b**.

---

[5] P. Thumbs, T. T. Ensfelder, M. Hillmeier, M. Wagner, M. Heiss, C. Scheel, A. Schön, M. Müller, S. Michalakis, S. Kellner, T. Carell, "Synthesis of Galactosyl-Queuosine and Distribution of Hypermodified Q-Nucleosides in Mouse Tissues" *Angew. Chem. Int. Ed.* **2020**, *59*, 12352 – 12356.

[6] NMR corresponded to data reported: H. Ohrui, G. H. Jones, J. G. Moffatt, M. L. Maddox, A. T. Christensen, S. K. Byram, "C-Glycosyl Nucleosides. V. Some Unexpected Observations on the Relative Stabilities of Compounds Containing Fused Five-Membered Rings with Epimerizable Substituents" *J. Am. Chem. Soc.* **1975**, *97*, 4602-4613.

12b: $[\alpha]_D$ = – 5.86 (*c* = 6.4, $CHCl_3$). ^1H NMR (300 MHz, $CDCl_3$) δ: 4.76-4.71 (m, 2H), 4.35 (ddd, *J* = 7.5, 6.0, 4.8 Hz, 1H), 4.05 (dd, *J* = 8.7, 6.0 Hz, 1H), 4.00 (dd, *J* = 8.7, 4.8 Hz, 1H), 3.91 (tdd, *J* = 6.8, 1.8, 0.9 Hz, 1H), 3.68 (s, 3H), 3.49 (ddd, *J* = 7.5, 1.8, 1.5 Hz, 1H), 2.78 (dd, *J* = 16.8, 7.5 Hz, 1H), 2.70 (dd, *J* = 16.8, 6.3 Hz, 1H), 1.44 (s, 3H), 1.42 (s, 3H), 1.35 (s, 3H), 1.31 (s, 3H); ^{13}C NMR (75 MHz, $CDCl_3$) δ: 171.5, 112.6, 109.1, 81.7, 81.1, 80.8, 77.8, 73.2, 67.0, 51.8, 33.4, 27.0, 25.8, 25.3, 24.7. HRMS (DART-TOF) *m/z:* $[M+H]^+$ calcd for $C_{15}H_{25}O_7$, 317.15948; found, 317.15903.

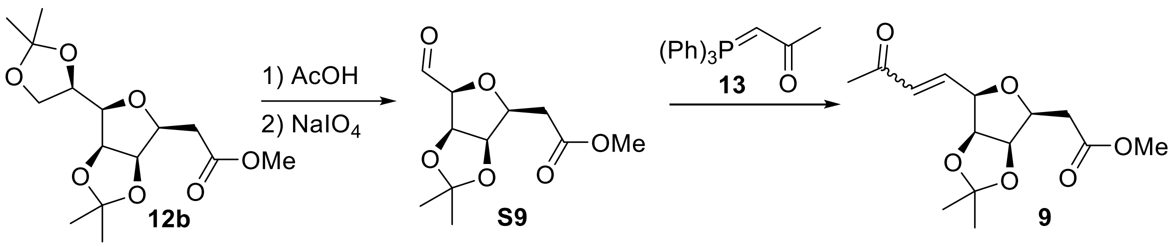

Methyl 2-((3aR,4S,6R,6aS)-2,2-dimethyl-6-(3-oxobut-1-en-1-yl)tetrahydrofuro[3,4-d][1,3]dioxol-4-yl)acetate (9): A solution of **12b** (0.9 g, 2.84 mmol) in a mixture of AcOH:H_2O (7:3; 28.4 mL) was vigorously stirred at r.t. Upon completion, the solvent was removed under reduced pressure. Then, the residue was dissolved in a mixture of EtOH:H_2O (5:5; 28.4 mL), and Na_2CO_3 was added until the mixture reached a pH of 7. Then, $NaIO_4$ (0.91 g, 4.26 mmol) was slowly added. After 30 min, the mixture solution was filtered through a sintered funnel and rinsed with EtOH (5 × 10 mL). The organic solvent was removed under reduced pressure, and the aqueous phase was extracted with EtOAc (3 × 15 mL). The combined organic phases were dried with Na_2SO_4, and the solvent was removed under reduced pressure to obtain **S9**, which was submitted to the next reaction without purification. To a solution of the phosphorus ylide **13** (1.8 g, 5.68 mmol) in anhydrous THF (22 mL) was added a solution of **S9** in anhydrous THF (6 mL) at 0 °C. Then, the reaction mixture was stirred at room temperature for 12 h. Next, the reaction was cooled to 0 °C and

quenched with 10 mL of H_2O. The organic solvent was evaporated under reduced pressure, and the aqueous phase was extracted with EtOAc (4 × 15 mL). The combined organic phases were dried with Na_2SO_4, and the solvent was removed under reduced pressure. The crude reaction was purified by column chromatography [SiO_2, hexanes/EtOAc 6:4] to give 485 mg (60%) of **9** as a diastereoisomeric mixture (~1:6). Small sample of both diastereoisomers were separated by column chromatography [SiO_2, hexanes/EtOAc 9:1].

Minor diastereoisomer-**9**: White solid; m.p.: 61-62 ºC. $[\alpha]_D$ = – 158.65 (c = 2.0, $CHCl_3$). ^1H NMR (300 MHz, $CDCl_3$) δ: 6.34 (dd, J = 11.7, 1.5 Hz, 1H), 6.17 (dd, J = 11.7, 6.3 Hz, 1H), 5.07 (dd, J = 6.0, 3.6 Hz, 1H), 4.88 (ddd, J = 6.3, 3.9, 1.5 Hz, 1H), 4.77 (dd, J = 6.0, 3.6 Hz, 1H), 3.96 (td, J = 6.6, 3.9 Hz, 1H), 3.71 (s, 3H), 2.84 (dd, J = 16.5, 6.6 Hz, 1H), 2.77 (dd, J = 16.5, 6.9 Hz, 1H), 2.25 (s, 3H), 1.45 (s, 3H), 1.29 (s, 3H); ^{13}C NMR (75 MHz, $CDCl_3$) δ: 198.6, 171.6, 143.2, 127.4, 112.5, 83.3, 81.4, 79.5, 77.6, 52.0, 33.7, 31.4, 26.0, 24.9. HRMS (ESI-TOF) m/z: $[M+H]^+$ calcd for $C_{14}H_{21}O_6$, 285.13381; found, 285.13373.

Major diastereoisomer-**9**: Colorless oil. $[\alpha]_D$ = – 21.7 (c = 0.67, $CHCl_3$). ^1H NMR (300 MHz, $CDCl_3$) δ: 6.76 (dd, J = 16.2, 5.7 Hz, 1H), 6.31 (dd, J = 16.2, 1.5 Hz, 1H), 4.81 (dd, J = 6.0, 3.6 Hz, 1H), 4.77 (dd, J = 6.0, 3.6 Hz, 1H), 4.16 (ddd, J = 6.0, 3.6, 1.5 Hz, 1H), 4.02 (td, J = 6.6, 3.6 Hz, 1H), 3.72 (s, 3H), 2.86 (dd, J = 16.8, 6.9 Hz, 1H), 2.79 (dd, J = 16.8, 6.6 Hz, 1H), 2.30 (s, 3H), 1.45 (s, 3H), 1.31 (s, 3H); ^{13}C NMR (75 MHz, $CDCl_3$) δ: 198.3, 171.5, 140.1, 133.0, 113.1, 82.7, 81.5, 81.0, 77.9, 52.0, 33.5, 27.1, 26.0, 25.1. HRMS (DART-TOF) m/z: $[M+H]^+$ calcd for $C_{14}H_{21}O_6$, 285.13326; found, 285.13271.

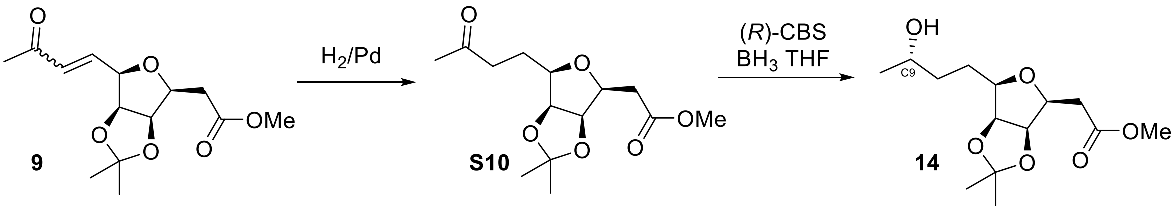

Methyl 2-((3aR,4S,6R,6aS)-2,2-dimethyl-6-(3-oxobutyl)tetrahydrofuro[3,4-d][1,3] dioxol-4-yl)acetate (S10): A mixture of **9** (0.4 g, 1.41 mmol), and Pd/C (10 wt.%, 40 mg) in EtOAc (14 mL) was stirred under H_2 atmosphere (one balloon) for 1 h. After this time, the reaction was filtered through a Celite pad and rinsed with EtOAc. The solvent was removed under reduced pressure. The residue was purified by column chromatography [SiO_2, hexanes/EtOAc, 7:3] to give 355 mg (88%) of **S10**.

S10: Colorless oil. $[\alpha]_D$ = + 10.96 (c = 2.4, $CHCl_3$). ^{1}H NMR (500 MHz, $CDCl_3$) δ: 4.72 (dd, J = 6.5, 3.0 Hz, 1H), 4.60 (dd, J = 6.0, 3.5 Hz, 1H), 3.87-3.83 (m, 1H), 3.71 (s, 3H), 3.49-3.45 (m, 1H), 2.79 (dd, J = 16.5, 7.0 Hz, 1H), 2.72 (dd, J = 16.5, 7.0 Hz, 1H), 2.59 (t, J = 7.0 Hz, 2H), 2.15 (s, 3H), 2.00-1.95 (m, 2H), 1.45 (m, 3H), 1.31 (s, 3H); ^{13}C NMR (125 MHz, $CDCl_3$) δ: 208.5, 171.7, 112.4, 81.6, 81.4, 80.5, 77.2, 51.8, 40.2, 33.5, 29.9, 26.0, 25.1, 22.6. HRMS (ESI-TOF) *m/z:* $[M+H]^+$ calcd for $C_{14}H_{23}O_6$, 287.14946; found, 287.14818.

Methyl 2-((3aR,4S,6R,6aS)-6-((S)-3-hydroxybutyl)-2,2-dimethyltetrahydrofuro[3,4-d] [1,3]dioxol-4-yl)acetate (14): To a solution of (*R*)-(+)-2-methyl-CBS-oxaborolidine (55.4 mg, 0.2 mmol) in anhydrous toluene (5 mL) at -10 °C, BH_3-THF complex (1M; 1.3 mL) was added dropwise. After 15 minutes, compound **S10** (286.3 mg; 1.0 mmol) in toluene (5 mL) was slowly added to reaction flask over 1 h. Upon completion, the reaction mixture was quenched with EtOH (6 mL) followed by H_2O (12 mL) and then stirring for 15 min. The aqueous phase was extracted with Et_2O (3 × 15 mL). The combined organic phases were dried with Na_2SO_4, and the solvent was removed under reduced pressure. The crude reaction was purified by column chromatography [SiO_2, hexanes/EtOAc 5:5] to give 245 mg (85%) of **14**.

14: Colorless oil. Reported as a diastereoisomeric mixture 3:1, favoring C9-(*S*)-isomer. The relationship was determined by ^{13}C NMR. ^{1}H NMR (300 MHz, $CDCl_3$) δ: 4.73 (dd, *J* = 6.0, 3.6 Hz, 1H), 4.62 (dd, *J* = 6.3, 3.6 Hz, 1H), 3.91-3.78 (m, 2H), 3.71 (s, 3H), 3.49 (apparent dt, *J* = 6.6, 3.6 Hz, 1H), 2.81 (dd, *J* = 16.8, 6.9 Hz, 1H), 2.73 (dd, *J* = 16.8, 6.9 Hz, 1H), 2.63 (br, 1OH), 1.85-1.76 (m, 2H), 1.62-1.53 (m, 2H), 1.46 (s, 3H), 1.32 (s, 3H), 1.20 (m, 3H), 1.18 (m, 3H); ^{13}C NMR (75 MHz, $CDCl_3$) δ: 171.63_{min}, 171.58_{maj}, 112.23_{maj}, 112.17_{min}, 81.87_{maj}, 81.75_{min}, 81.62_{maj}, 81.53_{min}, 81.23_{maj}, 81.15_{min}, 77.13_{min}, 77.08_{maj}, 67.71_{maj}, 67.62_{min}, 51.78(2C), 35.95_{maj}, 35.65_{min}, 33.38_{min}, 33.34_{maj}, 25.89(2C), 25.05(2C), 24.91_{maj}, 24.46_{min}, 23.34_{maj}, 23.26_{min}. HRMS (DART-TOF) *m/z:* $[M+H]^+$ calcd for $C_{14}H_{25}O_6$, 289.16456; found, 289.16431.

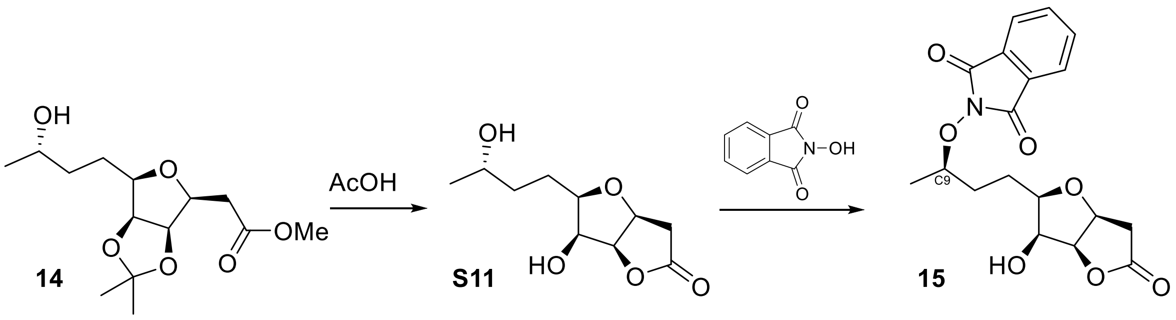

2-(((R)-4-((2R,3S,3aS,6aS)-3-hydroxy-5-oxohexahydrofuro[3,2-b]furan-2-yl)butan-2-yl)oxy)isoindoline-1,3-dione (15): A solution of **14** (0.2 g, 0.69 mmol) in a mixture of AcOH:H_2O (6:4; 6.9 mL) was heated at 120 ºC for 1 h under microwave irradiation in a sealed-pressure vial. Then, the solvent was evaporated under reduced pressure, and the residue was dissolved in a mixture of Et_2O:H_2O (5:5; 20 mL), and Na_2CO_3 was added until the mixture reached a pH of 7. The phases were separated, and the aqueous phase was extracted with Et_2O (3 × 15 mL). The combined organic phases were dried with Na_2SO_4, and the solvent was removed under reduced pressure to obtain **S11**, which was submitted to the next reaction without purification. The reaction crude of **S11**, PPh_3 (217 mg, 0.83 mmol) and

N-hydroxyphthalimide (135 mg, 0.83 mmol) were placed in a sealed-pressure vial. Then, the mixture was dissolved with anhydrous THF (6.9 mL) and stirred at 0 ºC. Then, DIAD (0.2 mL, 1.04 mmol) was added. After 5 minutes, the reaction mixture was heated at 40 ºC for 1 h under microwave irradiation. Next, the solvent was removed under reduced pressure, and the residue was purified by column chromatography [SiO_2, hexanes/EtOAc 5:5, then EtOAc] to give 174.5 g (70%) of **15**.

14:[7] White solid. Reported as a diastereoisomeric mixture 3:1, favoring C9-(*R*)-isomer. The relationship was determined by ^{13}C NMR. ^{1}H NMR (500 MHz, $CDCl_3$) δ: 7.85-7.83 (m, 2H), 7.79-7.77 (m, 2H), 5.05-5.02 (m, 1H), 4.64 (apparent td, *J* = 6.5, 3.5 Hz, 1H), 4.45-4.37 (m, 1H), 4.34-4.29 (m, 1H), 3.91-3.83 (m, 1H), 2.79 (dd, *J* = 18.5, 7.0 Hz, 1H), 2.72 (apparent dd, *J* = 18.5, 3.5 Hz, 1H), 2.09-1.71 (m, 4H), 1.36 (d, *J* = 6.5 Hz, 1H); ^{13}C NMR (125 MHz, $CDCl_3$) δ: 176.7(2C), 164.6(4C), 134.6(4C), 128.8(4C), 123.5(4C), 84.5_{maj}, 84.0_{min}, 83.5(2C), 82.8_{maj}, 82.4_{min}, 75.4(2C), 70.8(2C), 36.0(2C), 31.4_{maj}, 31.1_{min}, 24.5_{maj}, 23.9_{min}, 18.8_{maj}, 18.6_{min}. HRMS (DART-TOF) *m/z:* $[M+H]^+$ calcd for $C_{18}H_{20}NO_7$, 362.12343; found, 362.12317.

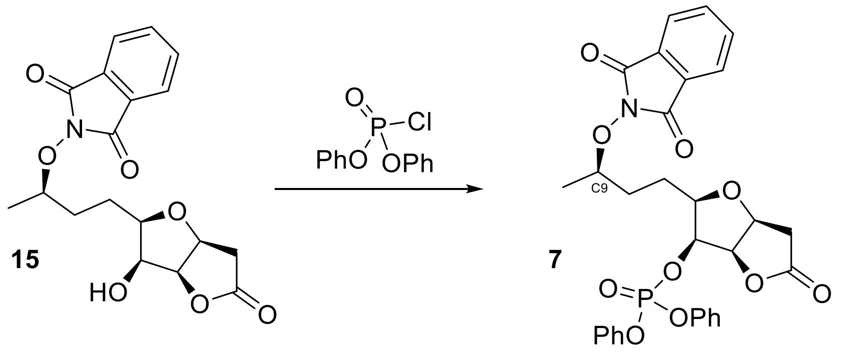

[7] O. Cortezano-Arellano, L. Quintero, F. Sartillo-Piscil, “Total Synthesis of Cephalosporolide E via a Tandem Radical/Polar Crossover Reaction. The Use of the Radical Cations under Nonoxidative Conditions in Total Synthesis” *J. Org. Chem*. **2015**, *80*, 2601-2608.

(2R,3S,3aR,6aS)-2-((R)-3-((1,3-dioxoisoindolin-2-yl)oxy)butyl)-5-oxohexahydrofuro-[3,2-b]furan-3-yl diphenyl phosphate (7): Employing the same procedure as for **2a**, **15** (144.5 mg, 0.4 mmol) was used to obtain **7**. The residue was purified by column chromatography [SiO_2, hexanes/EtOAc 5:5] to give 214 mg (90%) of **7**.

4:[7] Light yellow oil. Reported as a diastereoisomeric mixture 3:1, favoring C9-(*R*)-isomer. The relationship was determined by ^{13}C NMR. ^{1}H NMR (500 MHz, $CDCl_3$) δ: 7.83-7.80 (m, 2H), 7.76-7.73 (m, 2H), 7.35-7.24 (m, 8H), 7.19-7.16 (m, 2H), 5.21-5.07 (m, 2H), 4.69-4.65 (m, 1H), 4.32-4.26 (m, 1H), 4.06 (br, $0.25H_{min}$), 3.99 (br, $0.75H_{maj}$), 2.78 (dd, J = 18.5, 7.5 Hz, 1H), 2.71-2.65 (m, 1H), 1.90-1.64 (m, 4H), 1.27 (d, J = 5.0 Hz, 3H); ^{13}C NMR (125 MHz, $CDCl_3$) δ: 174.62(2C), 164.39(4C), 150.52(dd, J = 7.5, 5.0 Hz, 4C), 134.59_{min}(2C), 134.53_{maj}(2C), 129.98(4C), 129.84(4C), 129.10_{maj}(2C), 129.06_{min}(2C), 125.64(2C), 125.53(2C), 123.57_{min}(2C), 123.54_{maj}(2C), 120.30 (dd, J = 10.0, 5.0 Hz, 8C), 84.11_{maj}, 83.45min, 81.50_{maj} (d, J = 5.0 Hz), 81.47(2C), 81.12_{min}, 77.89_{min} (d, J = 6.3 Hz), 77.65_{maj} (d, J = 6.3 Hz), 75.59_{min}, 75.55_{maj}, 35.91_{maj}, 35.86_{min}, 31.17_{maj}, 31.11_{min}, 24.83_{maj}, 24.35_{min}, 18.92_{min}, 18.84_{maj}. HRMS (DART-TOF) *m/z:* $[M+H]^+$ calcd for $C_{30}H_{29}NO_{10}P$, 594.15236; found, 594.15304.

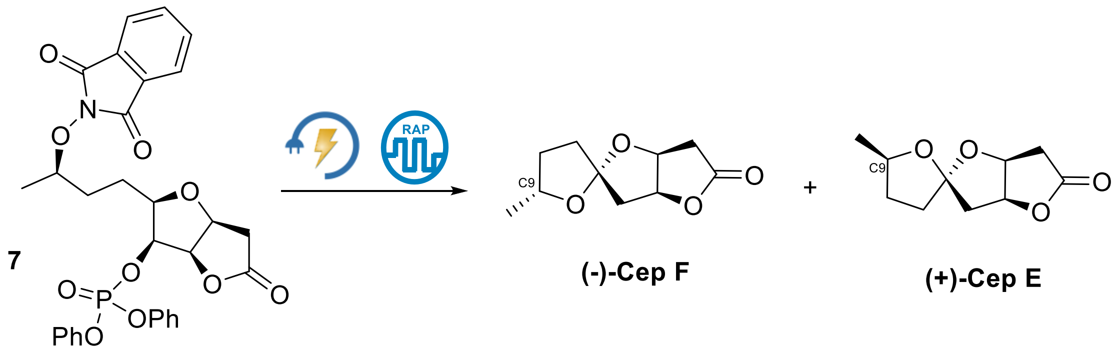

(-)-Cephalosporolide F and **(+)-Cephalosporolide E**: *N*-alkoxyphthalimide **7** (118.7 mg, 0.2 mmol) was subjected to the *general spirocyclization electrochemical procedure*. Current

= 17.1 mA (immersion depth: 3.4 cm; geometric area = 2.72 cm^2; current density = 6.3 mA cm^2), The residue was purified by column chromatography [SiO_2, hexanes/EtOAc 5:5] to give 5.5 mg (14%) of **(-)-Cep F**, and 3.9 mg (10%) of **(+)-Cep E** (along with their putative epimers). Note: No temperature rise was detected at the end of the experiment.

(-)-Cep F:[8] Reported as a diastereoisomeric mixture 4:1, favoring C9-(*R*)-isomer. The relationship was determined by ^{1}H NMR. $[\alpha]_D$ = – 79.0 (*c* = 0.5, $CHCl_3$). ^{1}H NMR (300 MHz, $CDCl_3$) δ: 5.09 (ddd, *J* = 6.6, 4.5, 2.4 Hz, 1H), 4.80 (td, *J* = 4.8, 1.8 Hz, 0.8H_{maj}), 4.73 (td, *J* = 4.8, 1.5 Hz, 0.2H_{min}), 4.26-4.15 (m, 1H), 2.79-2.64 (m, 2H), 2.56 (dd, *J* = 15.0, 6.6 Hz, 0.2H_{min}), 2.52 (dd, *J* = 14.7, 6.6 Hz, 0.8H_{maj}), 2.33 (dd, *J* = 15.0, 2.4 Hz, 1H), 2.19-1.98 (m, 3H), 1.80-1.69 (m, 1H), 1.28 (d, *J* = 6.3 Hz, 2.4H_{maj}), 1.22 (d, *J* = 6.3 Hz, 0.6H_{min}). HRMS (DART-TOF) *m/z:* $[M+H]^+$ calcd for $C_{10}H_{15}O_4$, 199.09649; found, 199.09635.

(+)-Cep E:[7] Reported as a diastereoisomeric mixture 3:1, favoring (*R*)-isomer. The relationship was determined by ^{13}C NMR. $[\alpha]_D$ = + 14.0 (*c* = 0.3, $CHCl_3$). ^{1}H NMR (400 MHz, $CDCl_3$) δ: 5.16 (t, *J* = 6.0 Hz, 1H), 4.91-4.86 (m, 1H), 4.25-4.14 (m, 1H), 2.78-2.63 (m, 2H), 2.45 (d, *J* = 14.0 Hz, 0.75Hmaj), 2.40 (d, *J* = 14.0 Hz, 0.25Hmin), 2.17-2.03 (m, 4H), 2.17-2.02 (m, 4H), 1.76-1.68(m, 0.25Hmin), 1.49-1.41(m, 0.75maj), 1.28 (d, *J* = 6.0 Hz, 0.75Hmin), 1.20 (d, *J* = 6.0 Hz, 2.25Hmaj); ^{13}C NMR (100 MHz, $CDCl_3$) δ: 176.00_{maj}, 175.91_{min}, 115.21_{maj}, 115.08_{min}, 83.52_{maj}, 83.45_{min}, 77.62_{min}, 77.44_{maj}, 77.36_{min}, 75.25_{maj}, 41.78_{maj}, 41.53_{min}, 37.83_{min}, 37.73_{maj}, 35.80_{min}, 34.33_{maj}, 32.10_{min}, 31.44_{maj}, 23.05_{min}, 21.06_{maj}.

[8] L. Xochicale-Santana, O. Cortezano-Arellano, B. A. Frontana-Uribe, V. M. Jimenez-Pérez, F. Sartillo-Piscil, "The Stereoselective Total Synthesis of the Elusive Cephalosporolide F" *J. Org. Chem.* **2023**, *88*, 4880-4885.

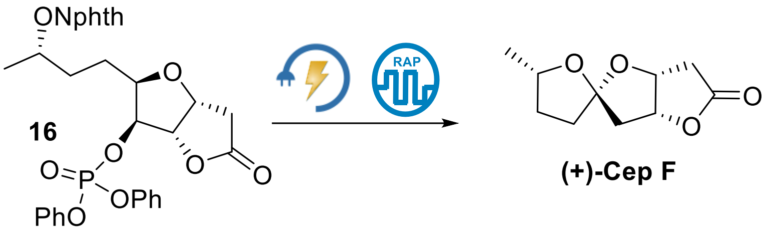

(+)-Cephalosporolide F: *N*-alkoxyphthalimide **16**[8] (59 mg, 0.1 mmol) was subjected to the *general spirocyclization electrochemical procedure.* For this reaction, a 5-mL glass vial was employed. Current = 15.6 mA (immersion depth: 3.1 cm; geometric area = 2.48 cm^2; current density = 6.3 mA cm^2). The residue was purified by column chromatography [SiO_2, hexanes/EtOAc 5:5] to give 4 mg (20%) of **(+)-Cep F**. Note: No temperature rise was detected at the end of the experiment.

(-)-Cep F:[8] ^{1}H NMR (300 MHz, $CDCl_3$) δ: 5.09 (ddd, *J* = 6.6, 4.5, 2.4 Hz, 1H), 4.80 (td, *J* = 4.5, 1.5 Hz, 1H), 4.24-4.18 (m, 1H), 2.75 (dd, *J* =18.6, 4.8 Hz, 1H), 2.67 (dd, *J* = 18.6, 1.8 Hz, 1H), 2.52 (dd, *J* = 14.7, 6.6 Hz, 1H), 2.33 (dd, *J* = 15.0, 1.8 Hz, 1H), 2.19-1.98 (m, 3H), 1.79-1.69 (m, 1H), 1.28 (d, *J* = 6.3 Hz, 3H); ^{13}C NMR (75 MHz, $CDCl_3$) δ: 175.8, 115.6, 84.0, 77.0, 76.7, 42.3, 37.1, 36.2, 32.6, 22.9. HRMS (DART-TOF) *m/z:* $[M+H]^+$ calcd for $C_{10}H_{15}O_4$, 199.09649; found, 199.09604.

Calculation of time and charge per mmol

Compounds **2a**, **4**, and **7** were reacted under electrochemical conditions at a reaction scale of 0.2 mmol, with 12 F mol^{-1}, and a current of I = 17.1 mA (*vide supra*).

Charge applied: $Q = F\,n\,N = 96485\ C\ mmol^{-1} \times 12\ \times 0.0002\ mol =\ 231.564\ C$

Time required: $t = \frac{Q}{I} = \frac{231.564\ C}{0.0171\ A} = 13541.7\ s = 3.76\ h$

$$Time\ per\ mmol = \frac{3.76\ h}{0.2\ mmol} = 18.8\ h\ mmol^{-1}$$

$$Charge\ per\ mmol = \frac{231.564\ C}{0.2\ mmol} = 1157.8\ C\ mmol^{-1}$$

Compounds **16** was reacted under electrochemical conditions at a reaction scale of 0.1 mmol, with 12 F mol^{-1}, and a current of I = 15.6 mA (*vide supra*).

Charge applied: $Q = F\,n\,N = 96485\ \times 12\ \times 0.0001\ mol =\ 115.782\ C$

Time required: $t = \frac{Q}{I} = \frac{115.782\ C}{0.0156\ A} = 7421.9\ s = 2.06\ h$

$$Time\ per\ mmol = \frac{2.06\ h}{0.1\ mmol} = 20.6\ h\ mmol^{-1}$$

$$Charge\ per\ mmol = \frac{115.782\ C}{0.1\ mmol} = 1157.82\ C\ mmol^{-1}$$

Compounds **2a**, and **4** were reacted under photoredox conditions at a reaction scale of 0.2 mmol and a reaction time of 3 h (*vide supra*).

$$Time\ per\ mmol = \frac{3\ h}{0.2\ mmol} = 15\ h\ mmol^{-1}$$

Compound **16** was reacted under photoredox conditions at a reaction scale of 0.025 mmol and a reaction time of 2 h (see Ref. *J. Org. Chem.* **2023**, *88*, 4880-4885).

$$Time\ per\ mmol = \frac{2\ h}{0.025\ mmol} = 80\ h\ mmol^{-1}$$

Cyclic voltammograms

*Cyclic voltammogram of N-alkoxyphthalimide **2a***

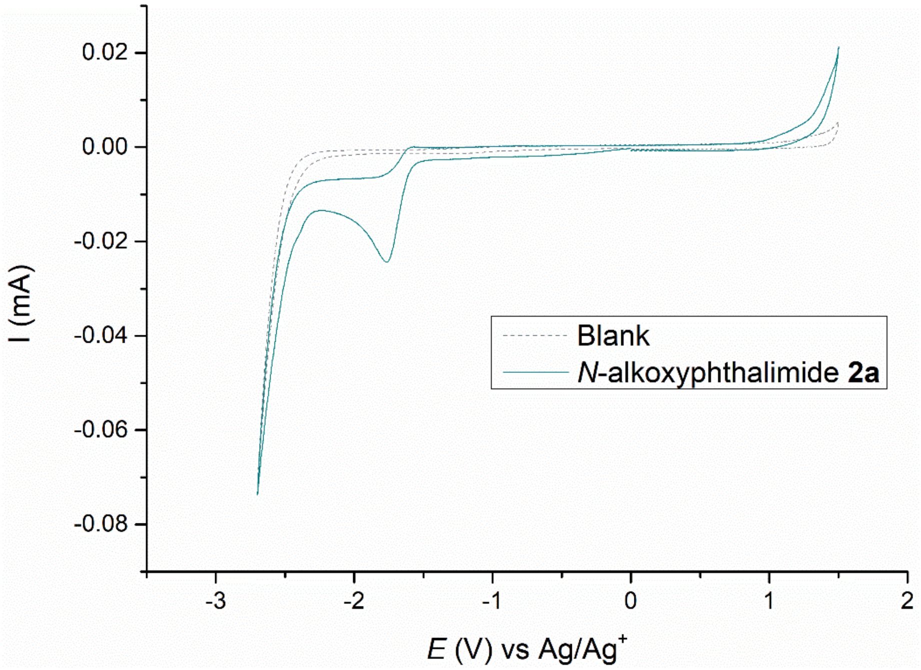

Cyclic voltammetry of **2a** (2 mM), 0.1 M $nBuN_4PF_6$ in CH_2Cl_2, at 0.1 Vs^{-1}. WE: Glassy carbon, CE: Pt, RE: Ag/Ag^+.

*Cyclic voltammograms of N-alkoxyphthalimides **2a-2e***

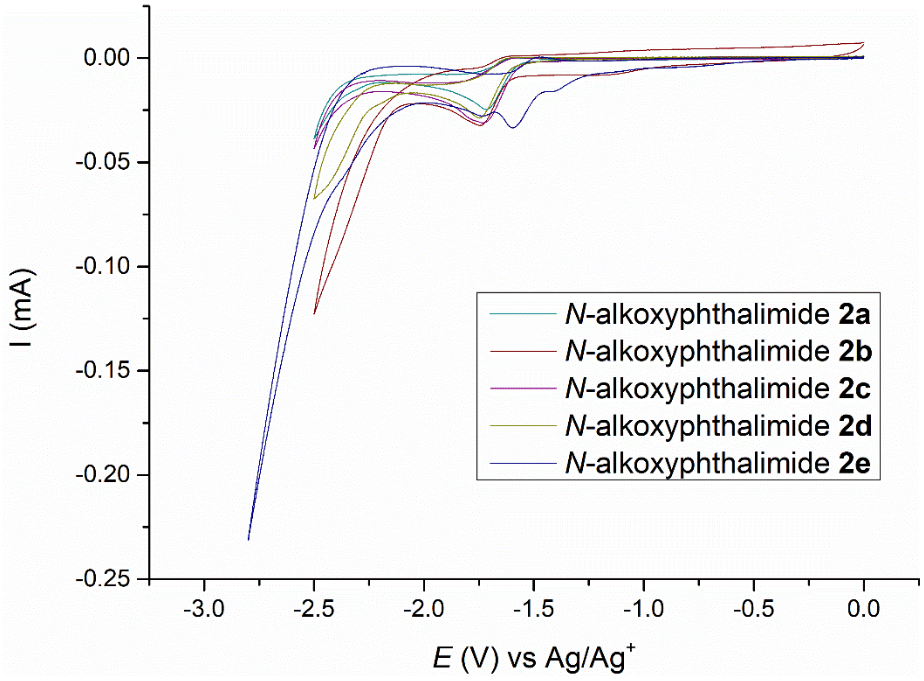

Cyclic voltammetry of **2a-2e** (2 mM), 0.1 M $nBuN_4PF_6$ in CH_2Cl_2, at 0.1 Vs^{-1}. WE: Glassy carbon, CE: Pt, RE: Ag/Ag^+.

*Cyclic voltammogram of N-alkoxyphthalimide **4***

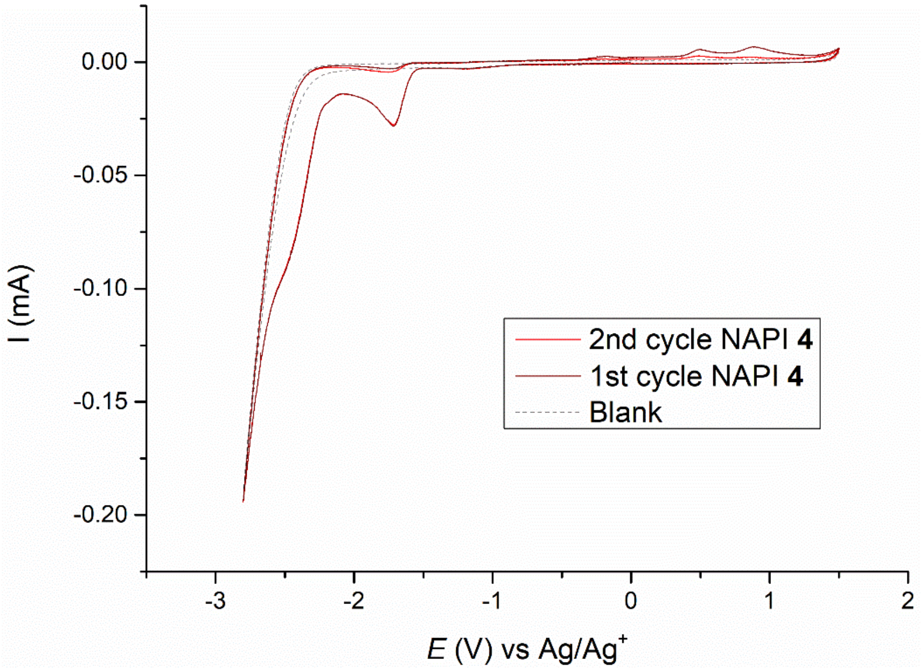

Cyclic voltammetry of **4** (2 mM), 0.1 M $nBuN_4PF_6$ in CH_2Cl_2, at 0.1 Vs^{-1}. WE: Glassy carbon, CE: Pt, RE: Ag/Ag^+.

NMR Spectra

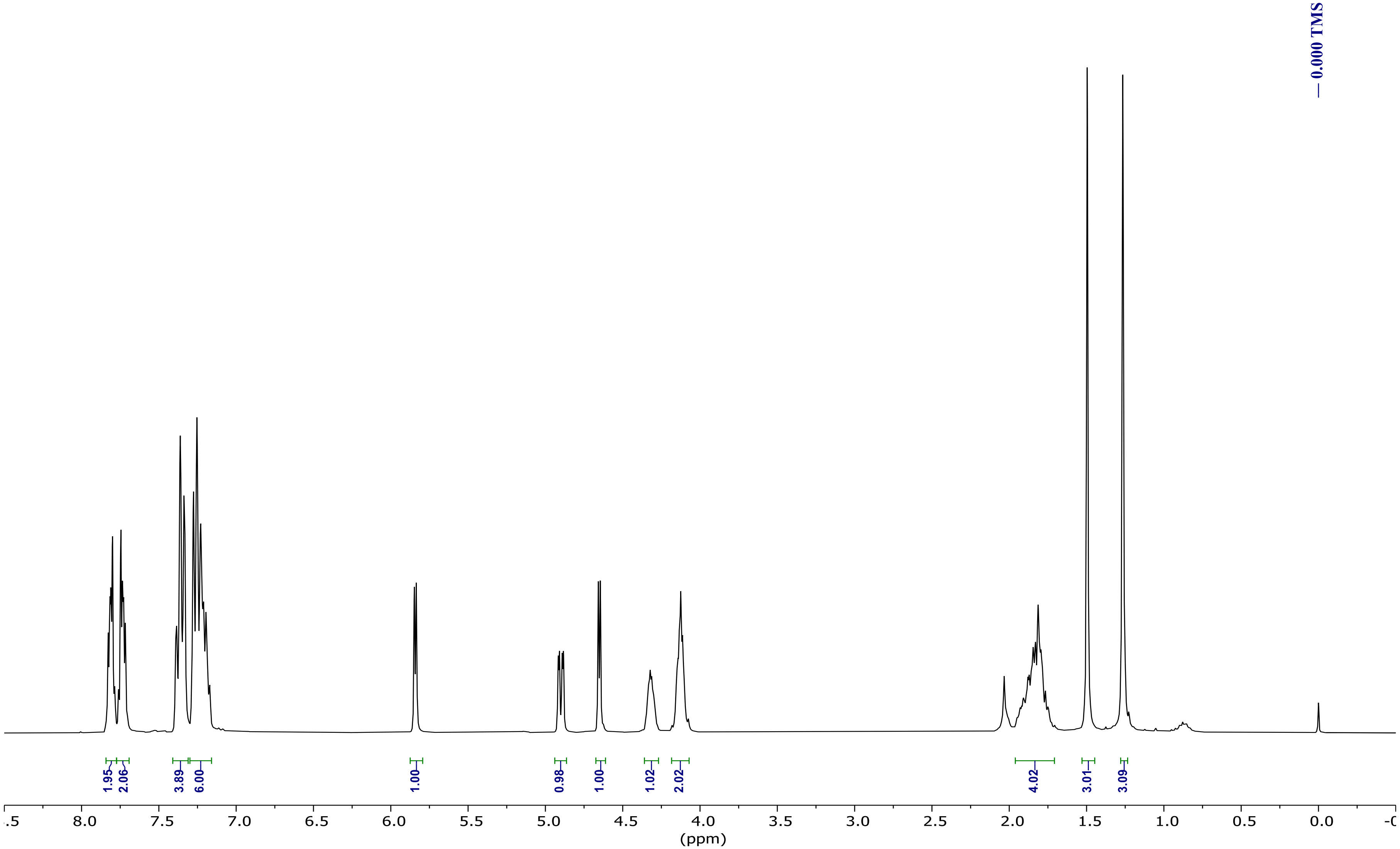

N-alkoxyphthalimide (**2a**): ^{1}H-NMR (300 MHz, $CDCl_3$)

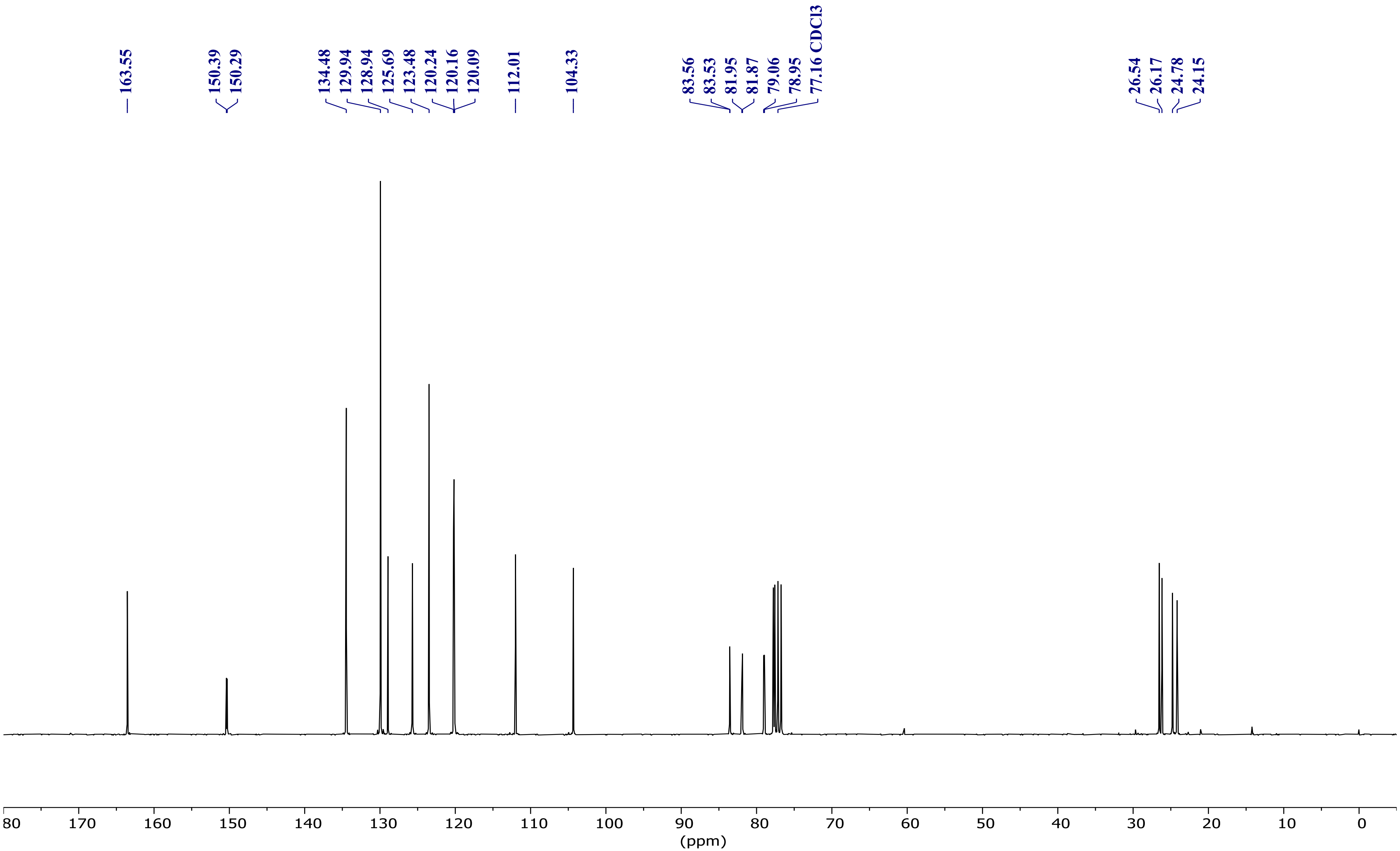

*N-alkoxyphthalimide (**2a**) ^{13}C-NMR (75 MHz, $CDCl_3$)*

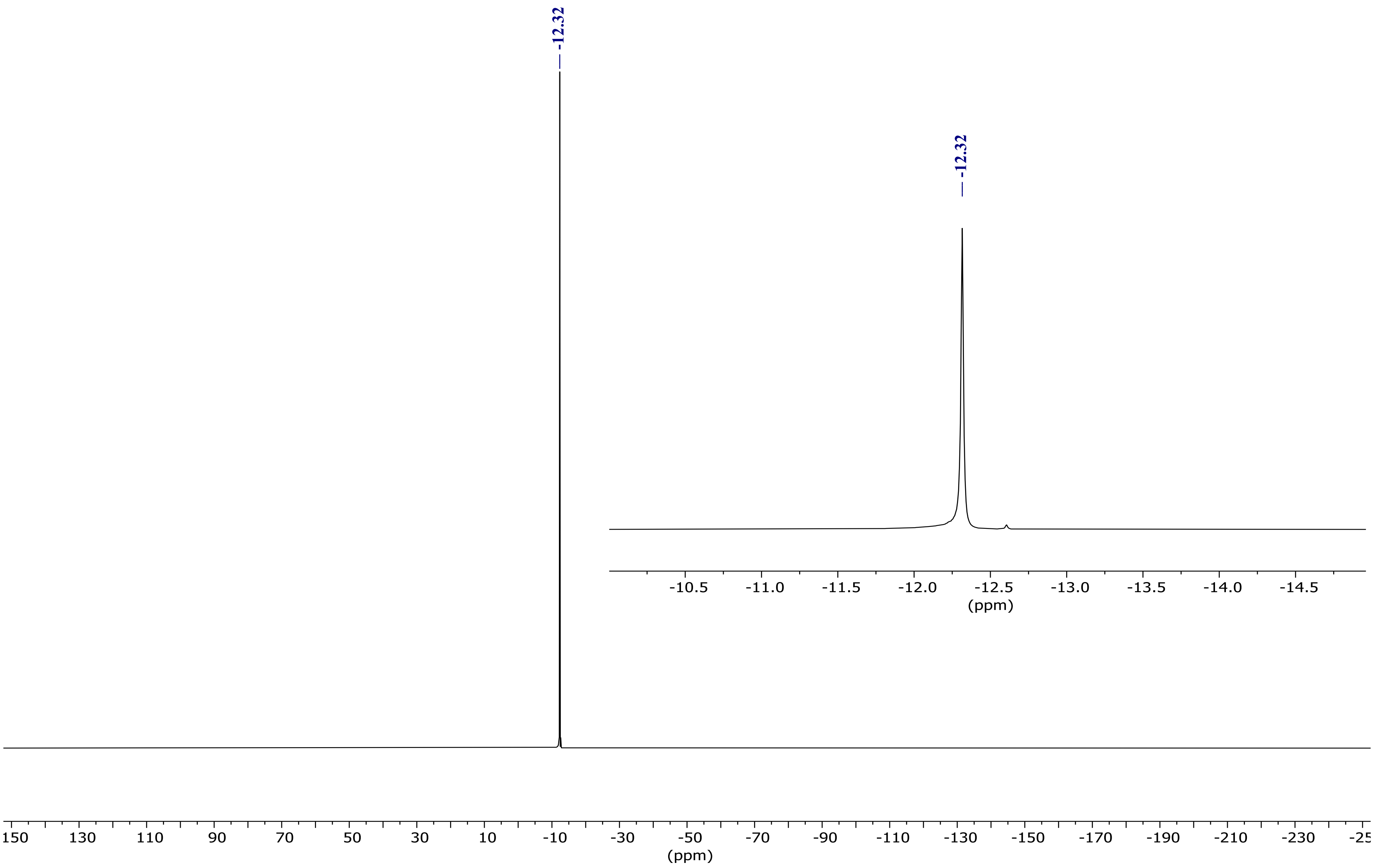

*N-alkoxyphthalimide (**2a**) $^{31}P\{^{1}H\}$-NMR (202 MHz, $CDCl_3$)*

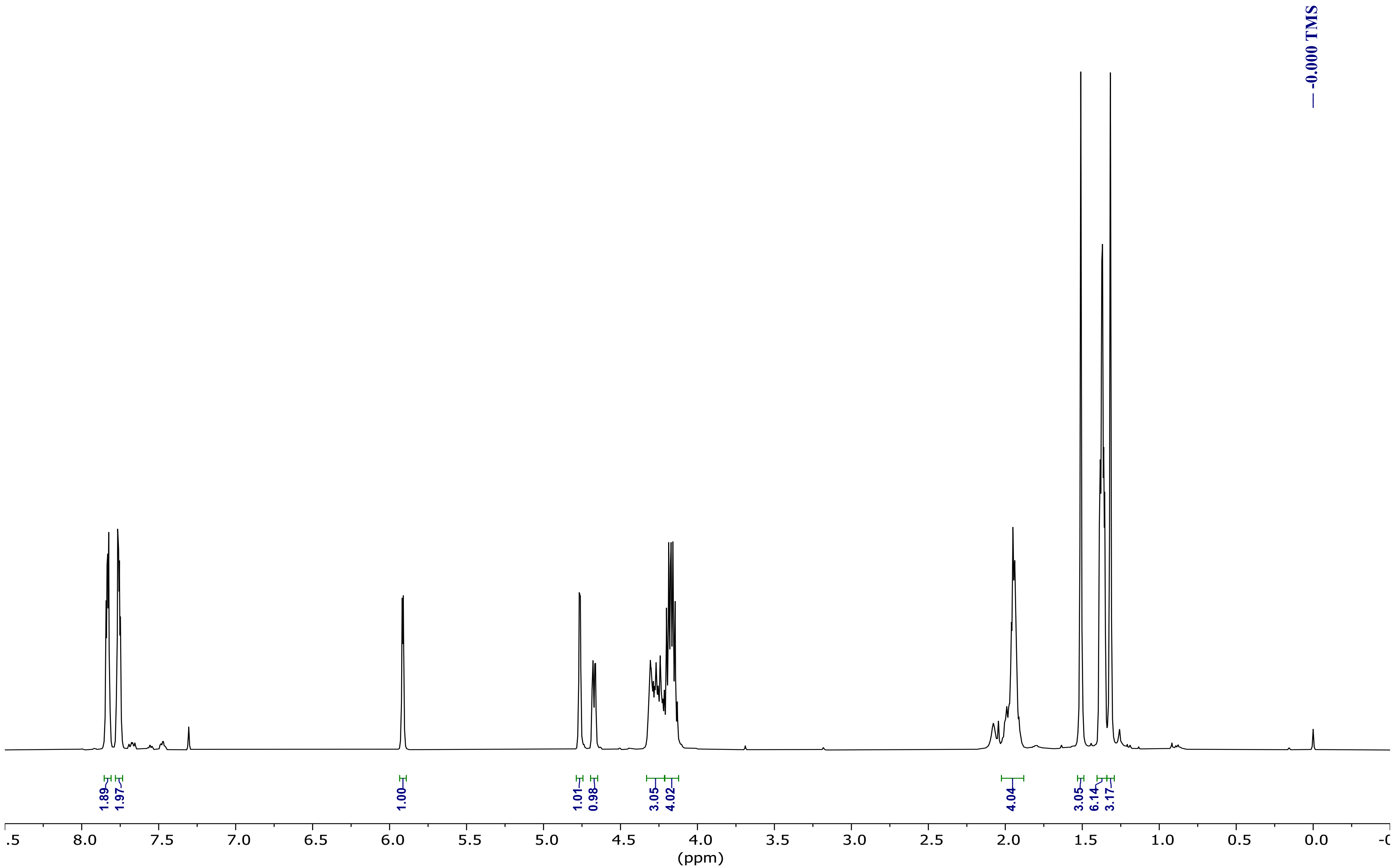

*N-alkoxyphthalimide (**2b**) ^{1}H-NMR (300 MHz, CDCl$_3$)*

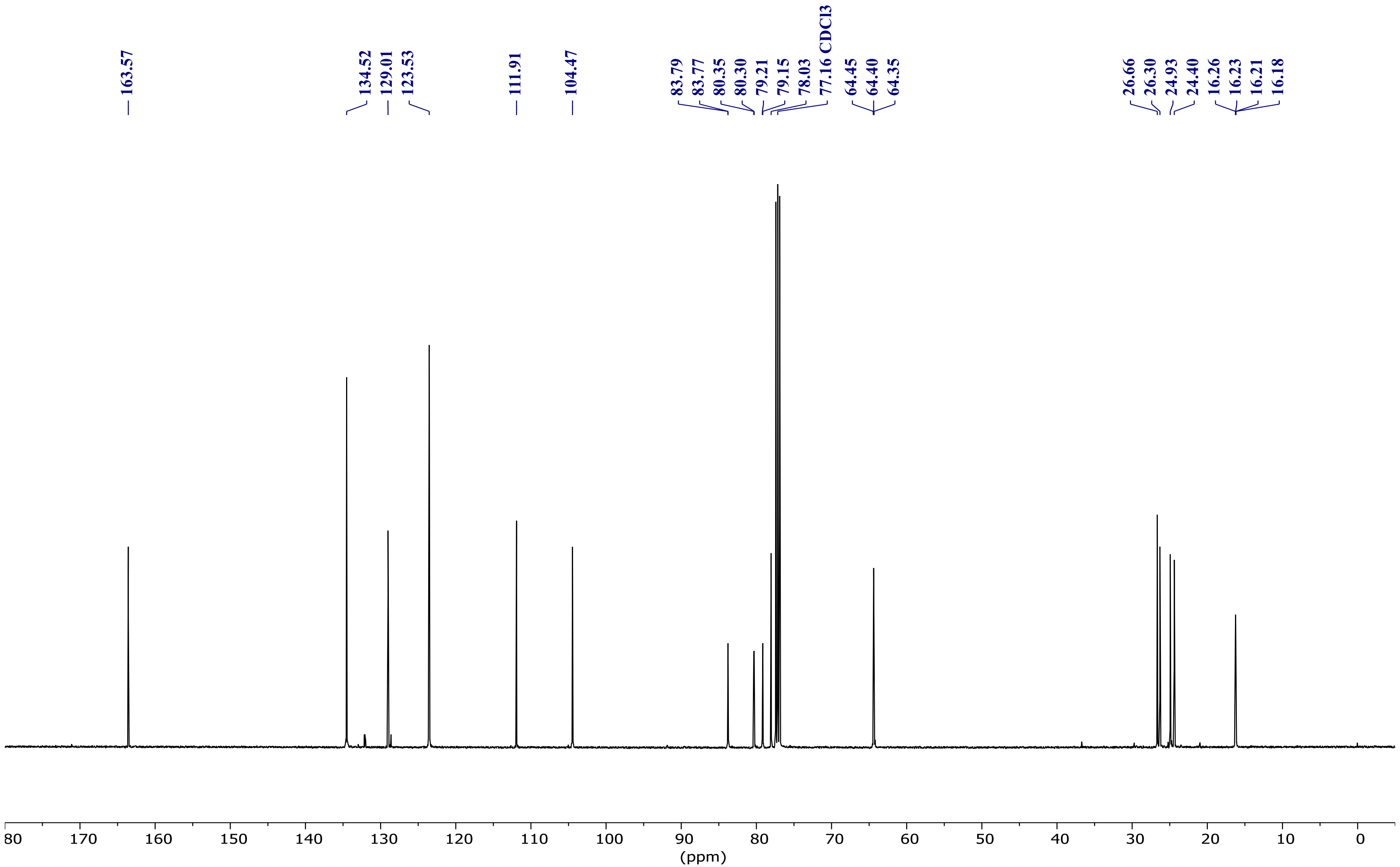

*N-alkoxyphthalimide (**2b**) ^{13}C-NMR (75 MHz, $CDCl_3$)*

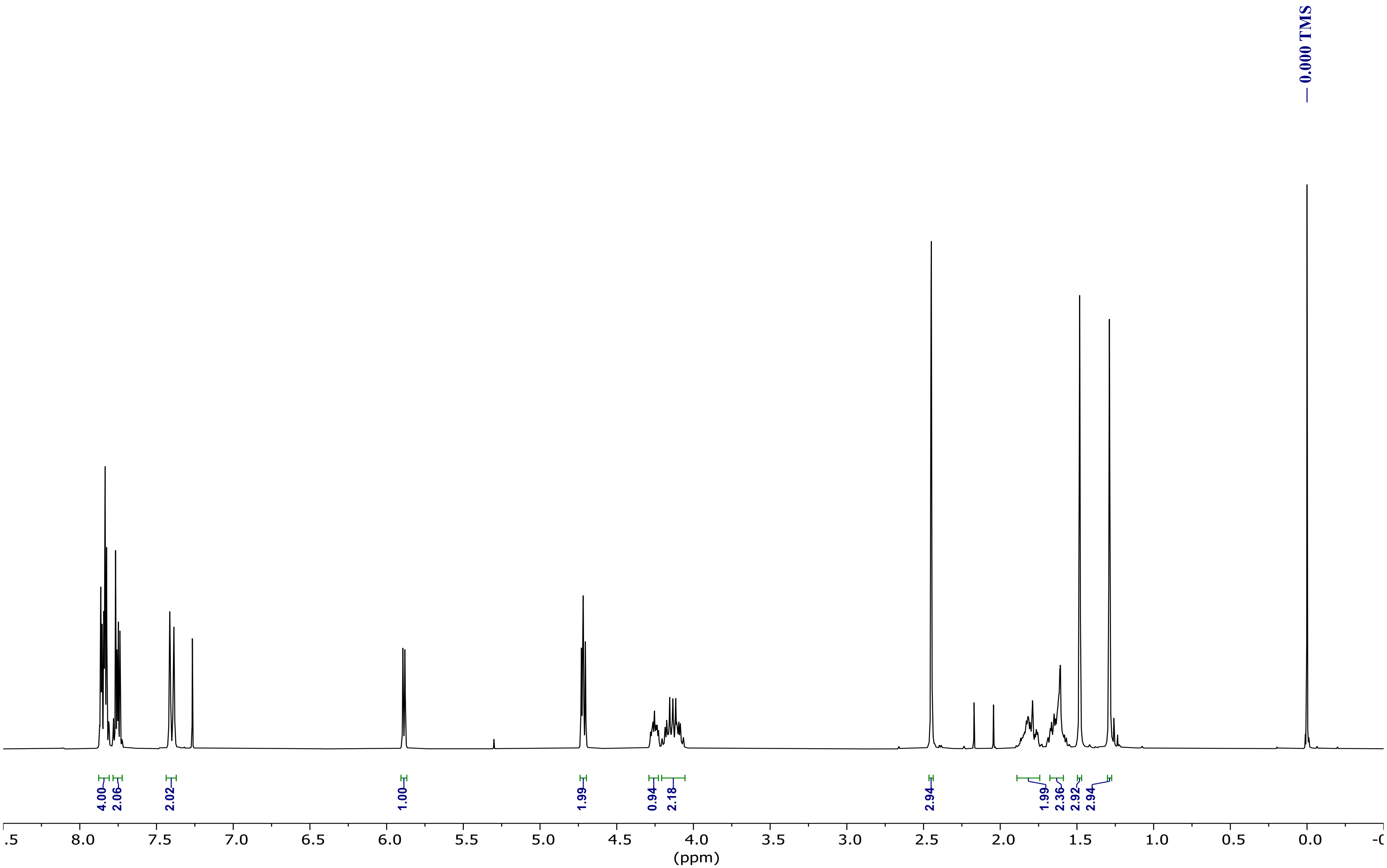

N-alkoxyphthalimide (**2c**) ^{1}H-NMR (300 MHz, $CDCl_3$)

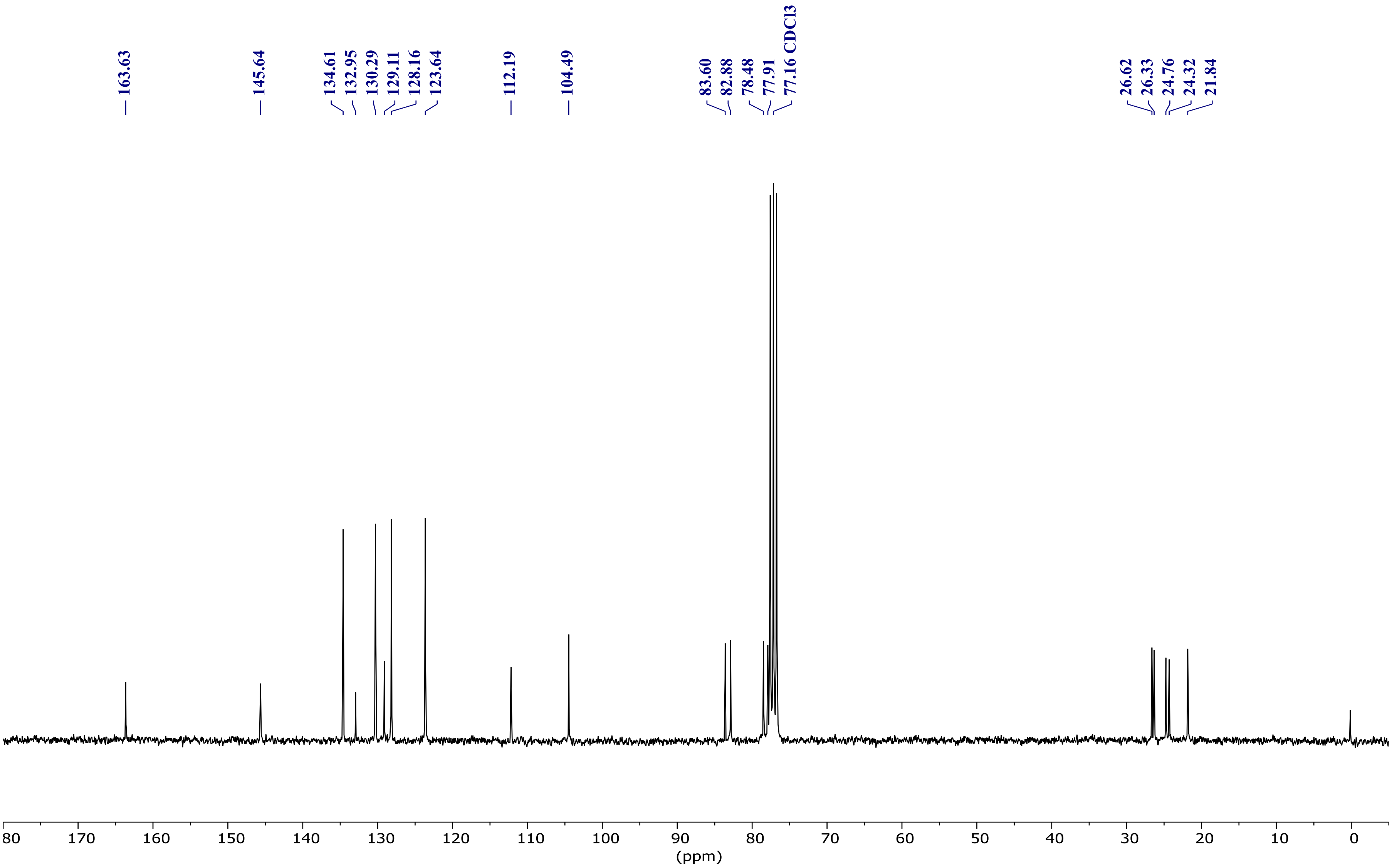

N-alkoxyphthalimide (**2c**)^{13}C-NMR (75 MHz, $CDCl_3$)

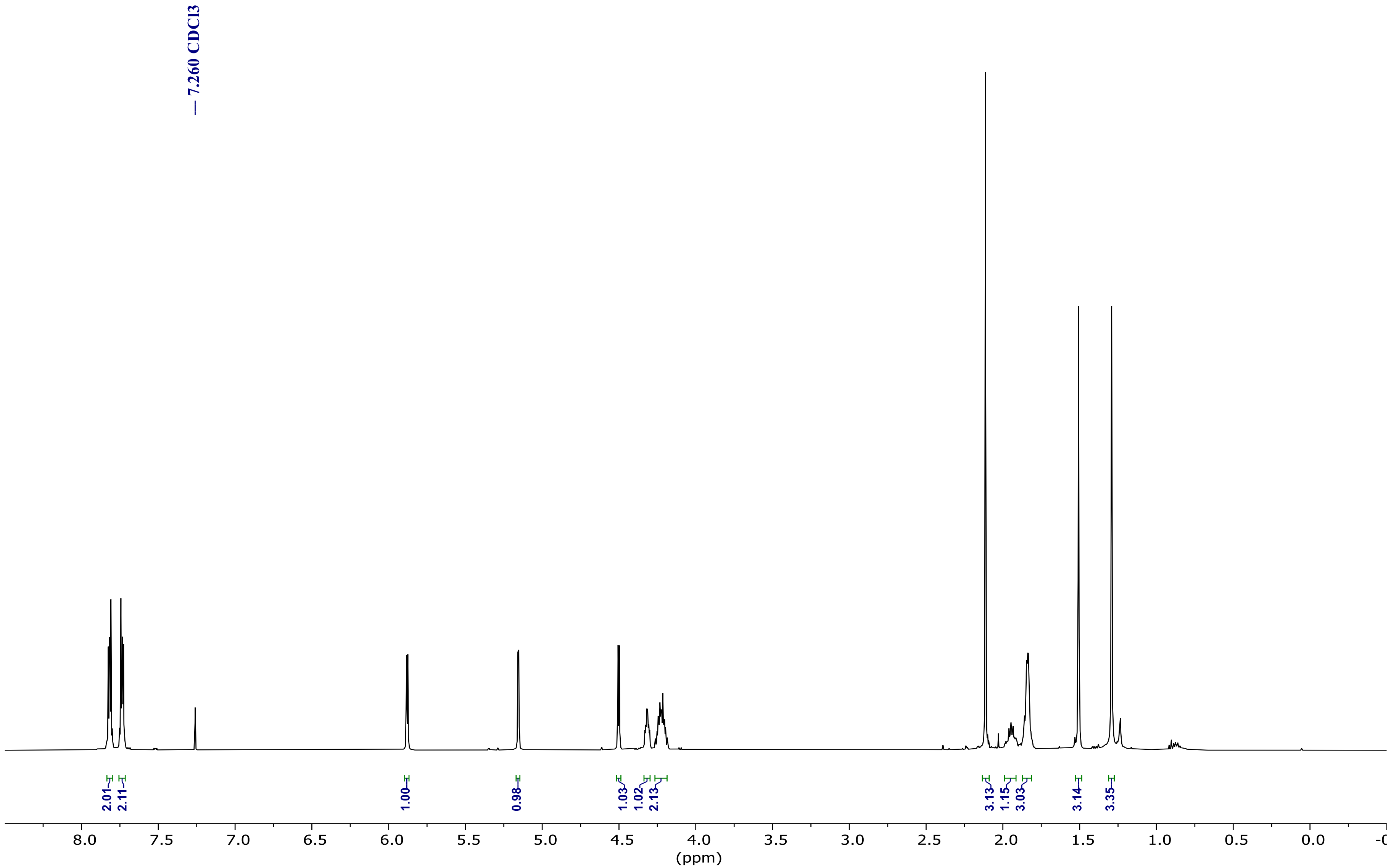

*N-alkoxyphthalimide (**2d**) ^{1}H-NMR (500 MHz, $CDCl_3$)*

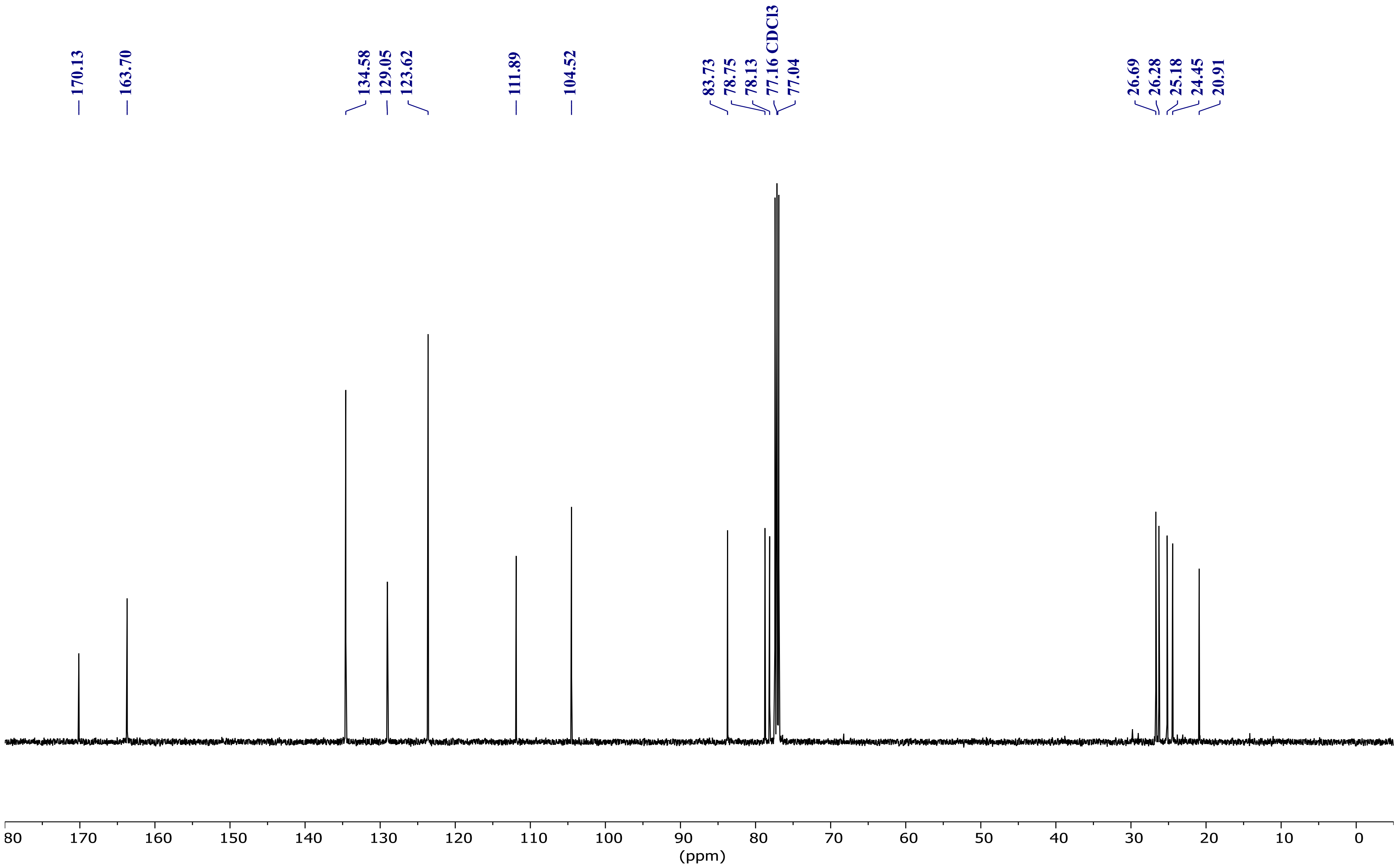

*N-alkoxyphthalimide (**2d**)^{13}C-NMR (125 MHz, $CDCl_3$)*

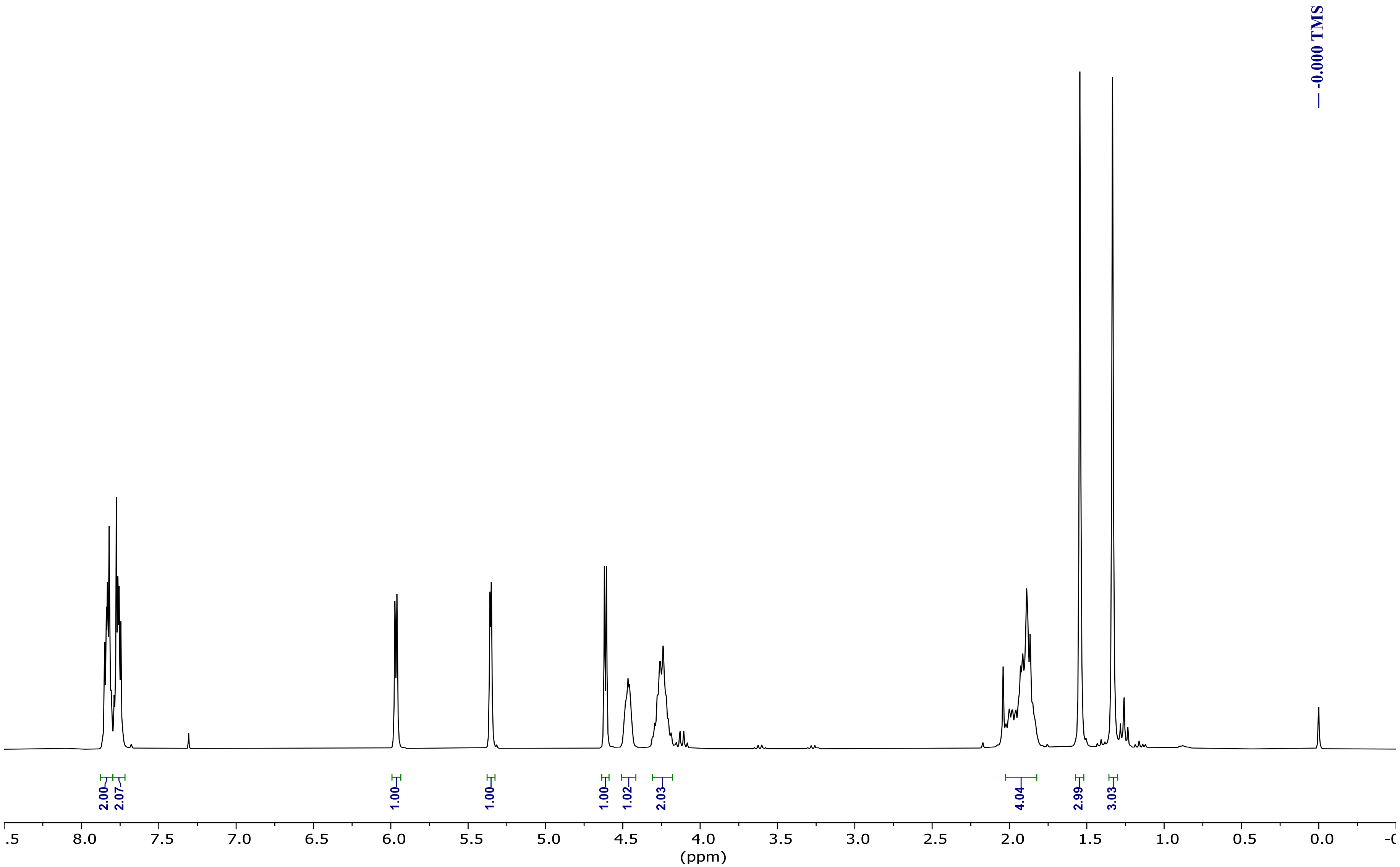

*N-alkoxyphthalimide (**2e**)* ^{1}*H-NMR (300 MHz, CDCl* $_{3}$ *)*

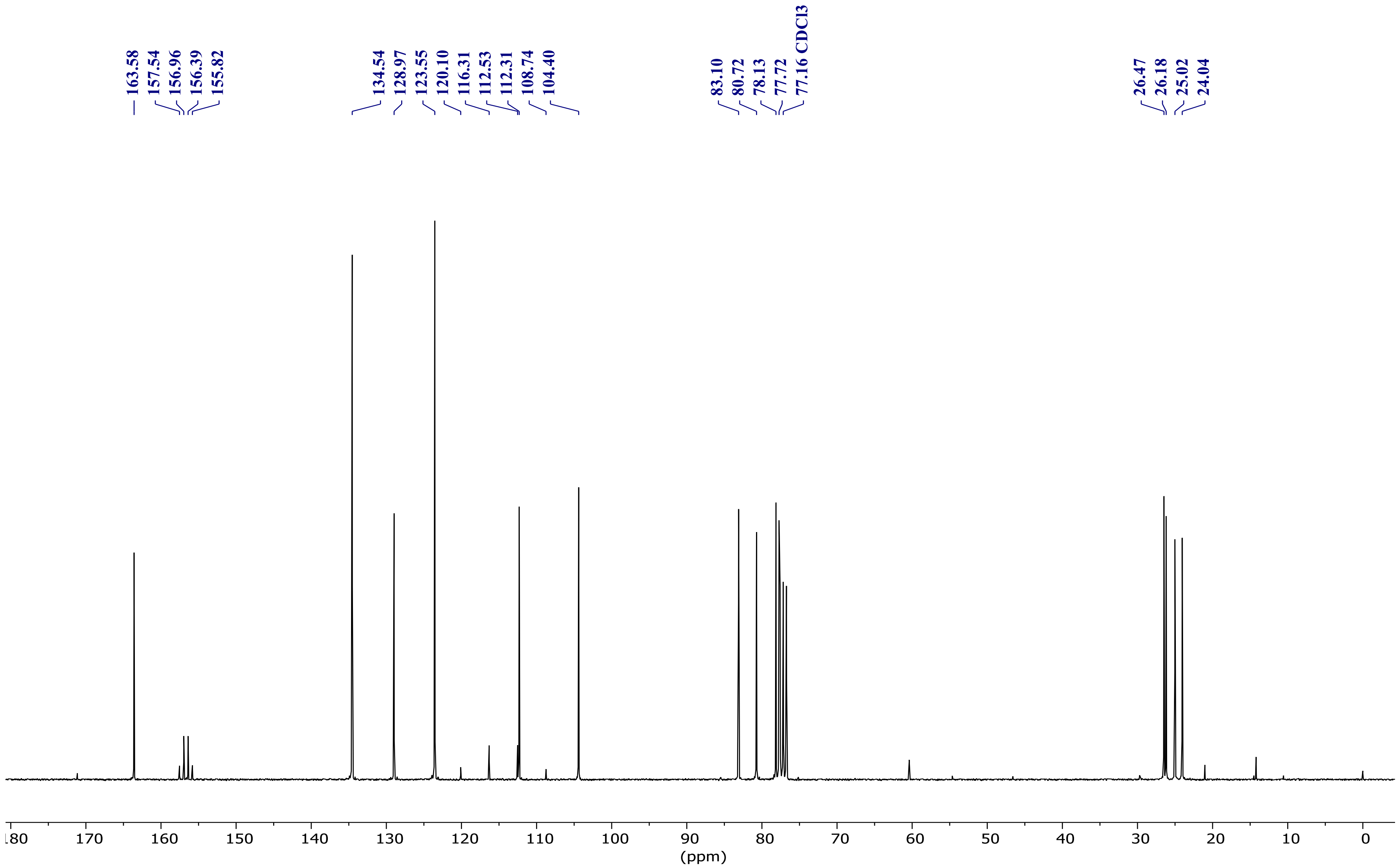

*N-alkoxyphthalimide (**2e**): ^{13}C-NMR (75 MHz, $CDCl_3$)*

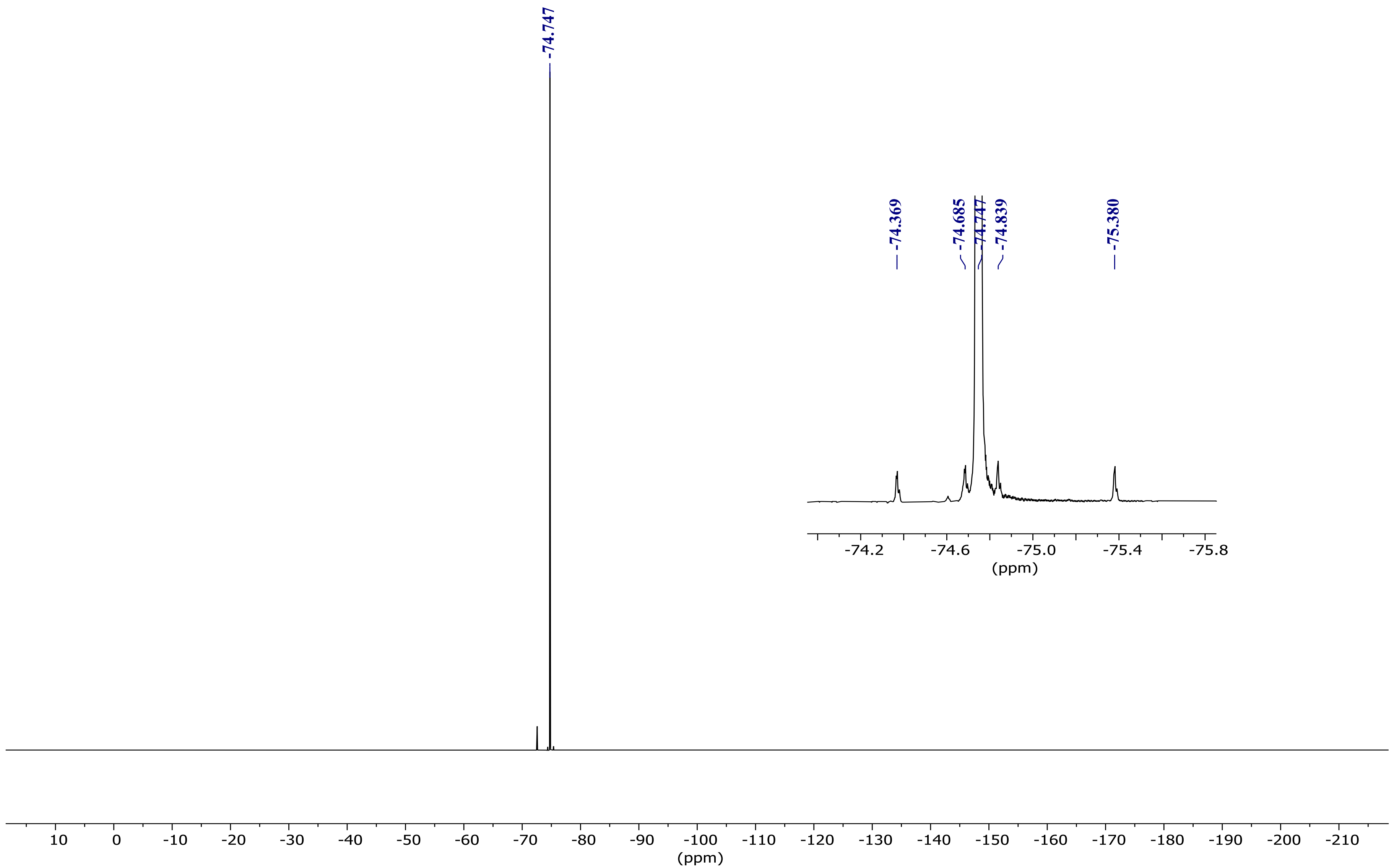

*N-alkoxyphthalimide (**2e**): ^{19}F-NMR (282 MHz, $CDCl_3$)*

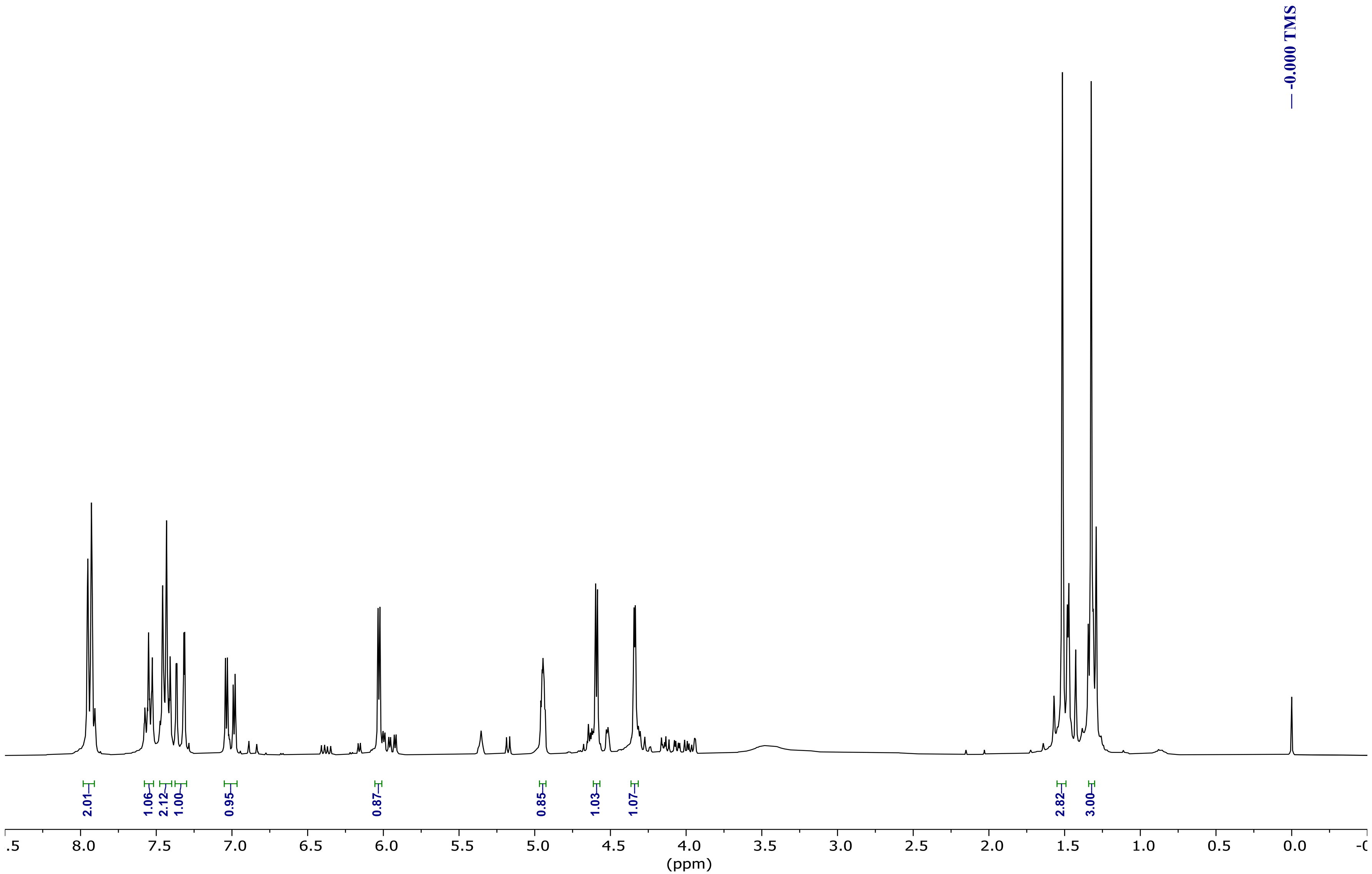

*α,β-unsaturated ketone (**S5**): ^{1}H-NMR (300 MHz, $CDCl_3$)*

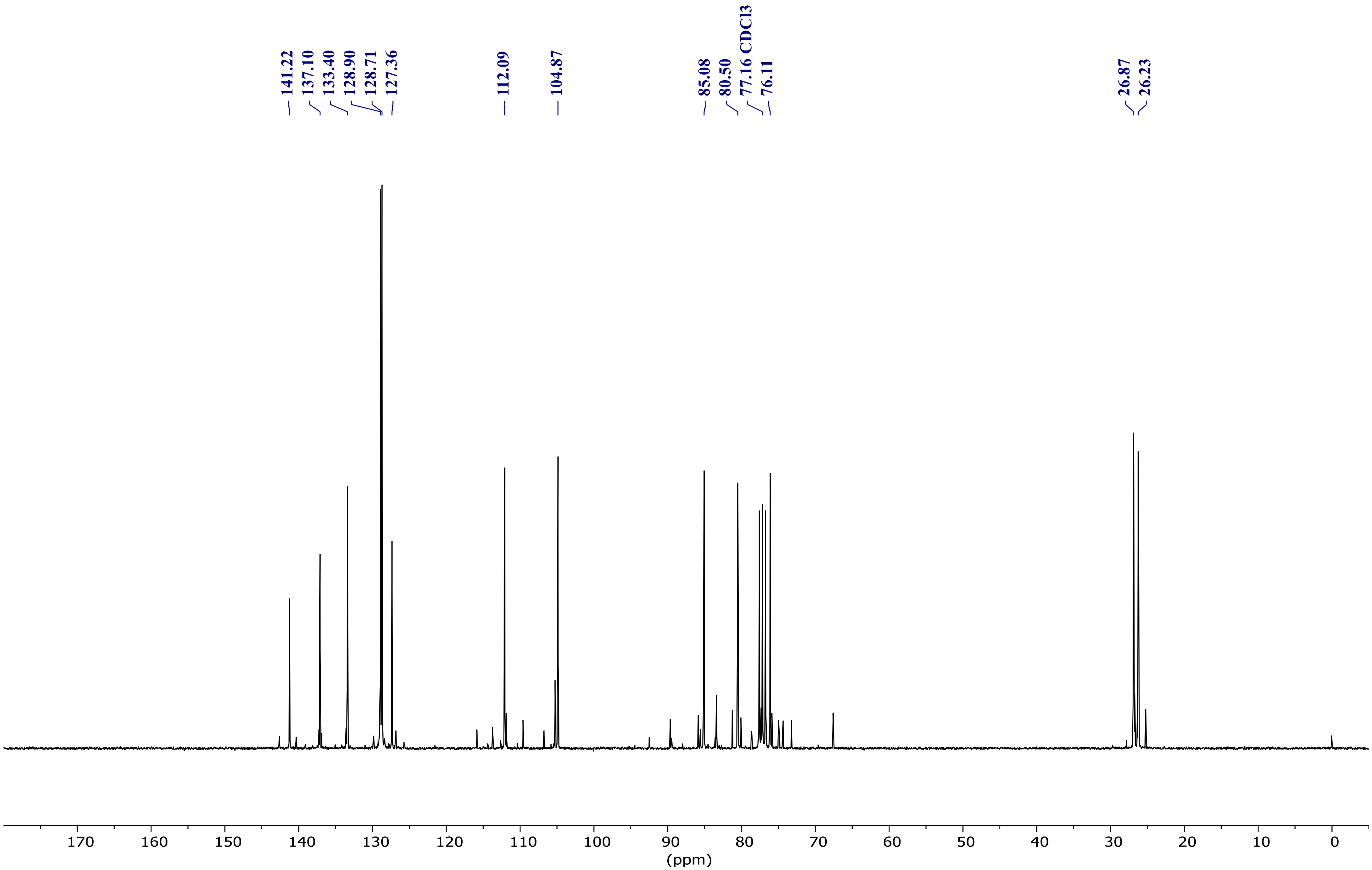

*α,β-unsaturated ketone (**S5**): ^{13}C-NMR (75 MHz, $CDCl_3$)*

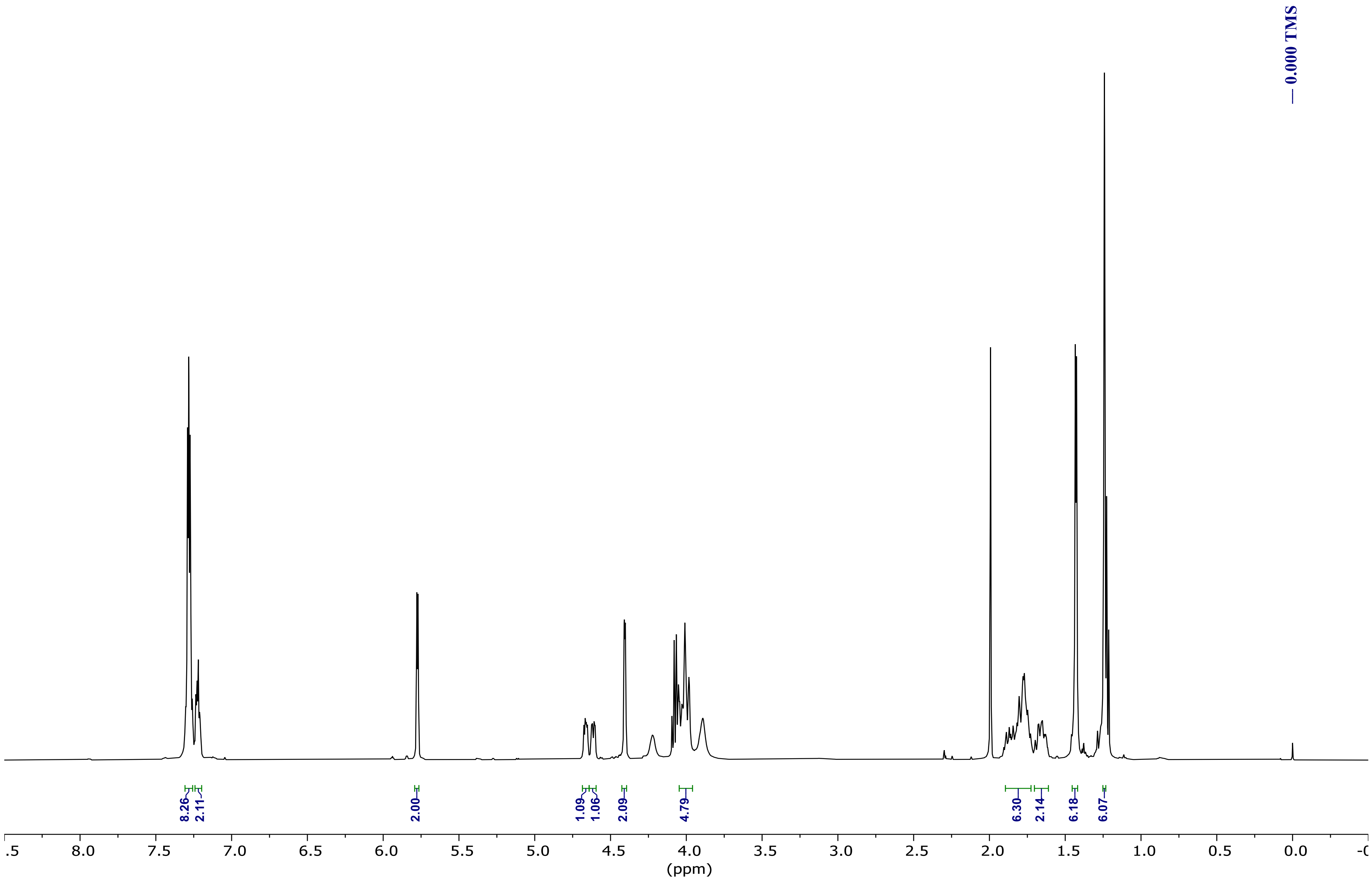

(3aR,5R,6S,6aR)-5-(3-hydroxy-3-phenylpropyl)-2,2-dimethyltetrahydrofuro[2,3-d][1,3]dioxol-6-ol (**S6**)*: ^{1}H-NMR (500 MHz, $CDCl_3$)*

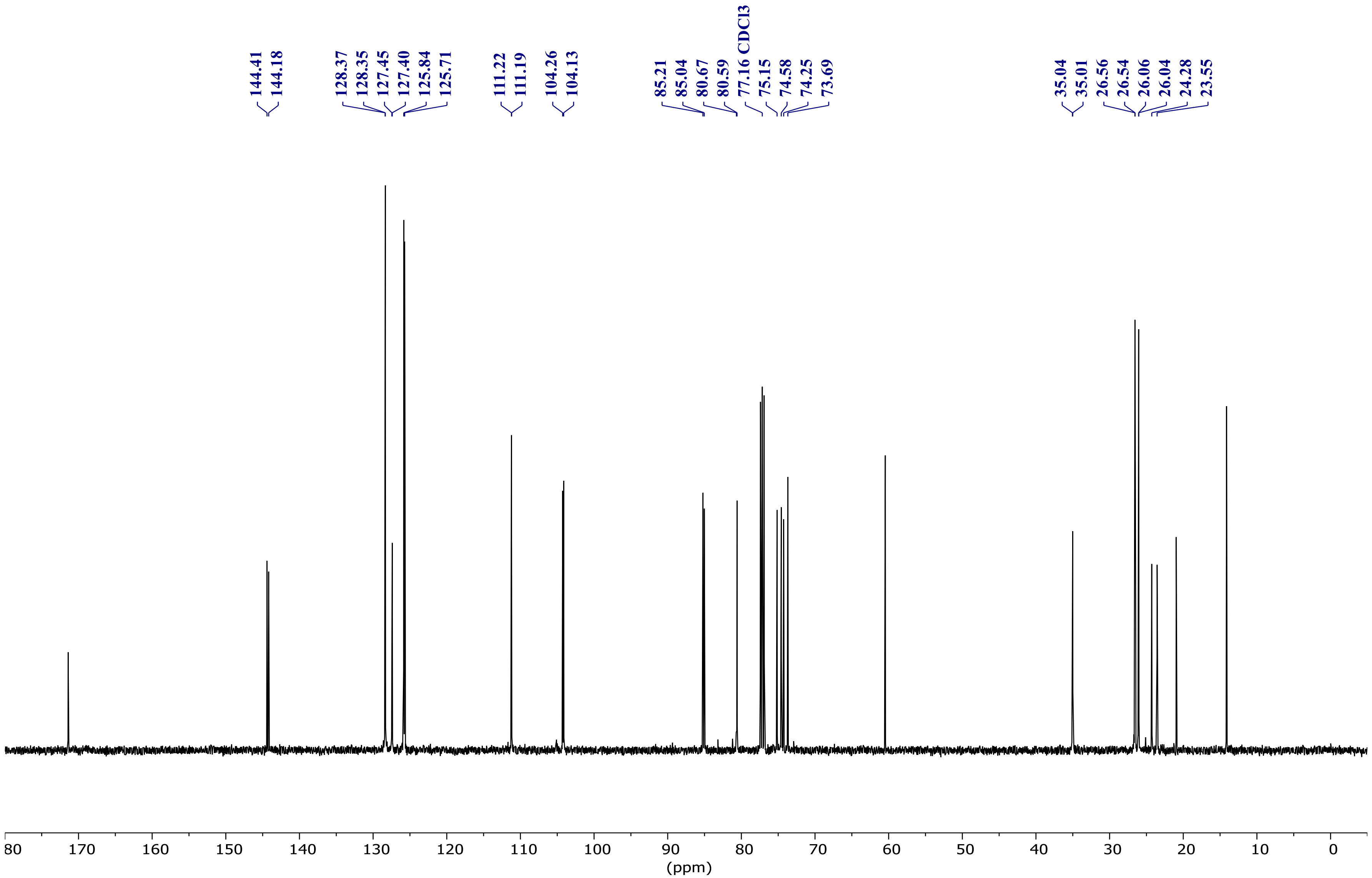

*(3aR,5R,6S,6aR)-5-(3-hydroxy-3-phenylpropyl)-2,2-dimethyltetrahydrofuro[2,3-d][1,3]dioxol-6-ol (**S6**): ^{13}C-NMR (125 MHz, $CDCl_3$)*

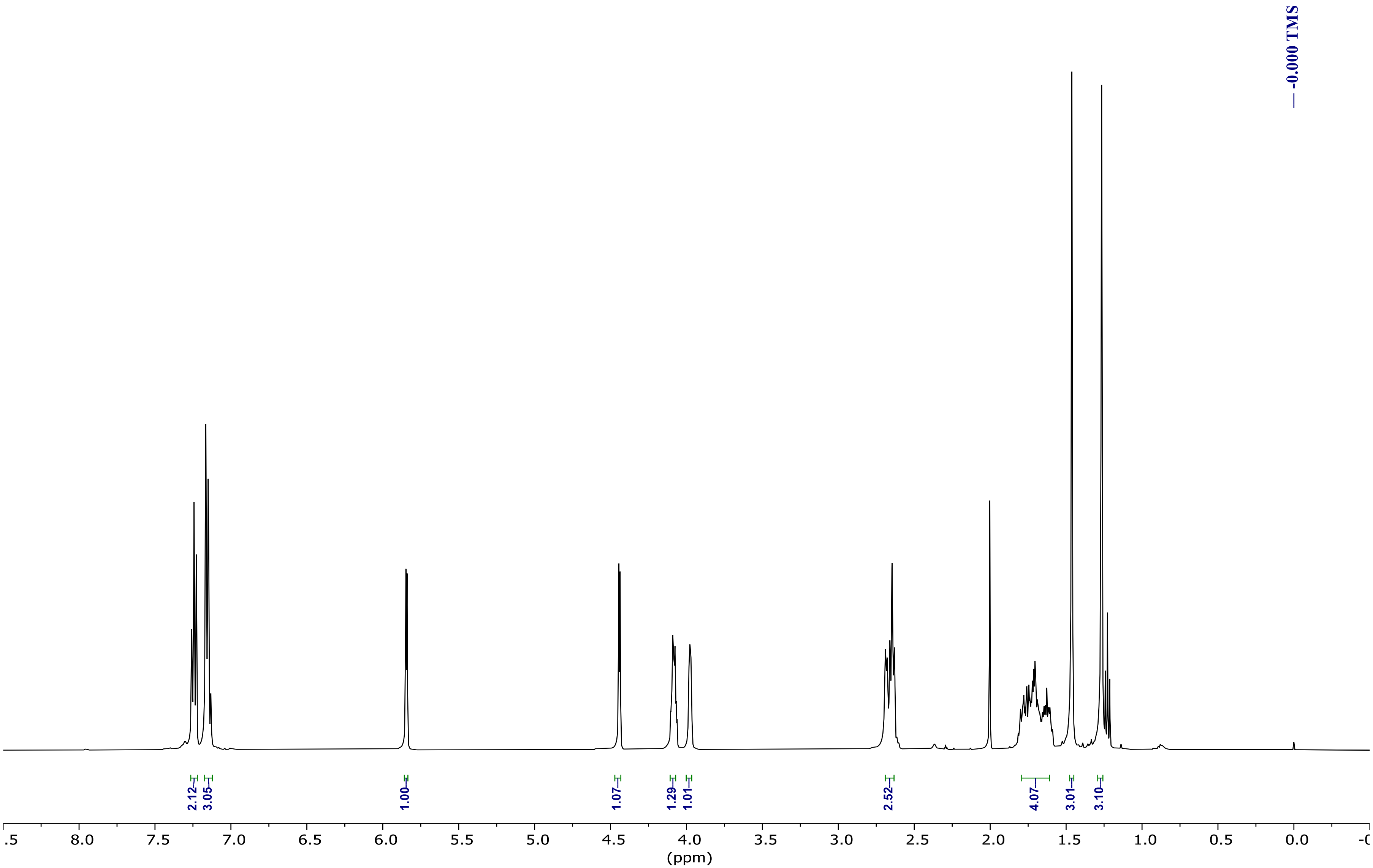

(3aR,5R,6S,6aR)-2,2-dimethyl-5-(3-phenylpropyl)tetrahydrofuro[2,3-d][1,3]dioxol-6-ol (**S7**)*:* ^{1}H-NMR (500 MHz, $CDCl_3$)

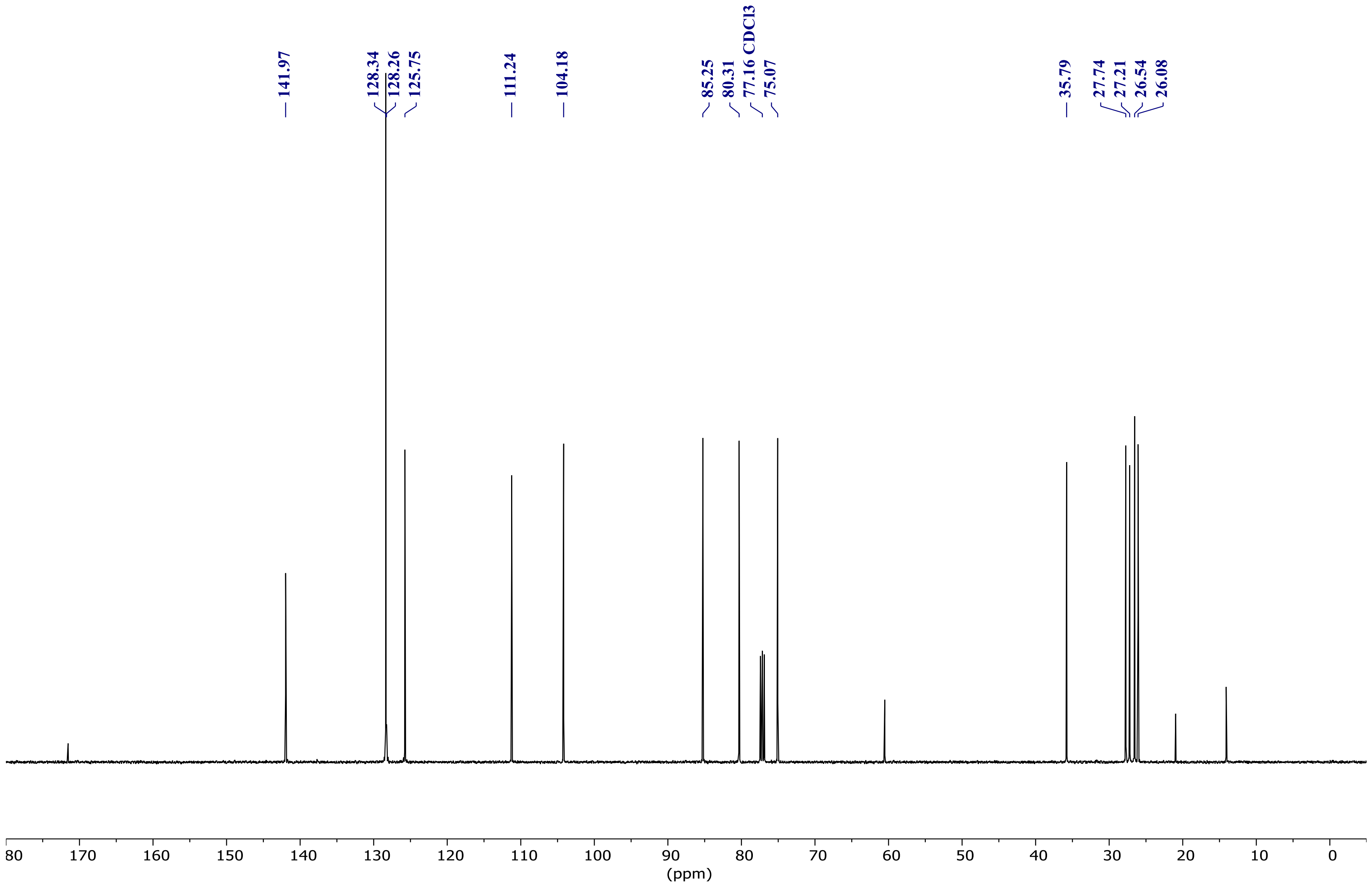

*(3aR,5R,6S,6aR)-2,2-dimethyl-5-(3-phenylpropyl)tetrahydrofuro[2,3-d][1,3]dioxol-6-ol (**S7**): ^{13}C-NMR (125 MHz, $CDCl_3$)*

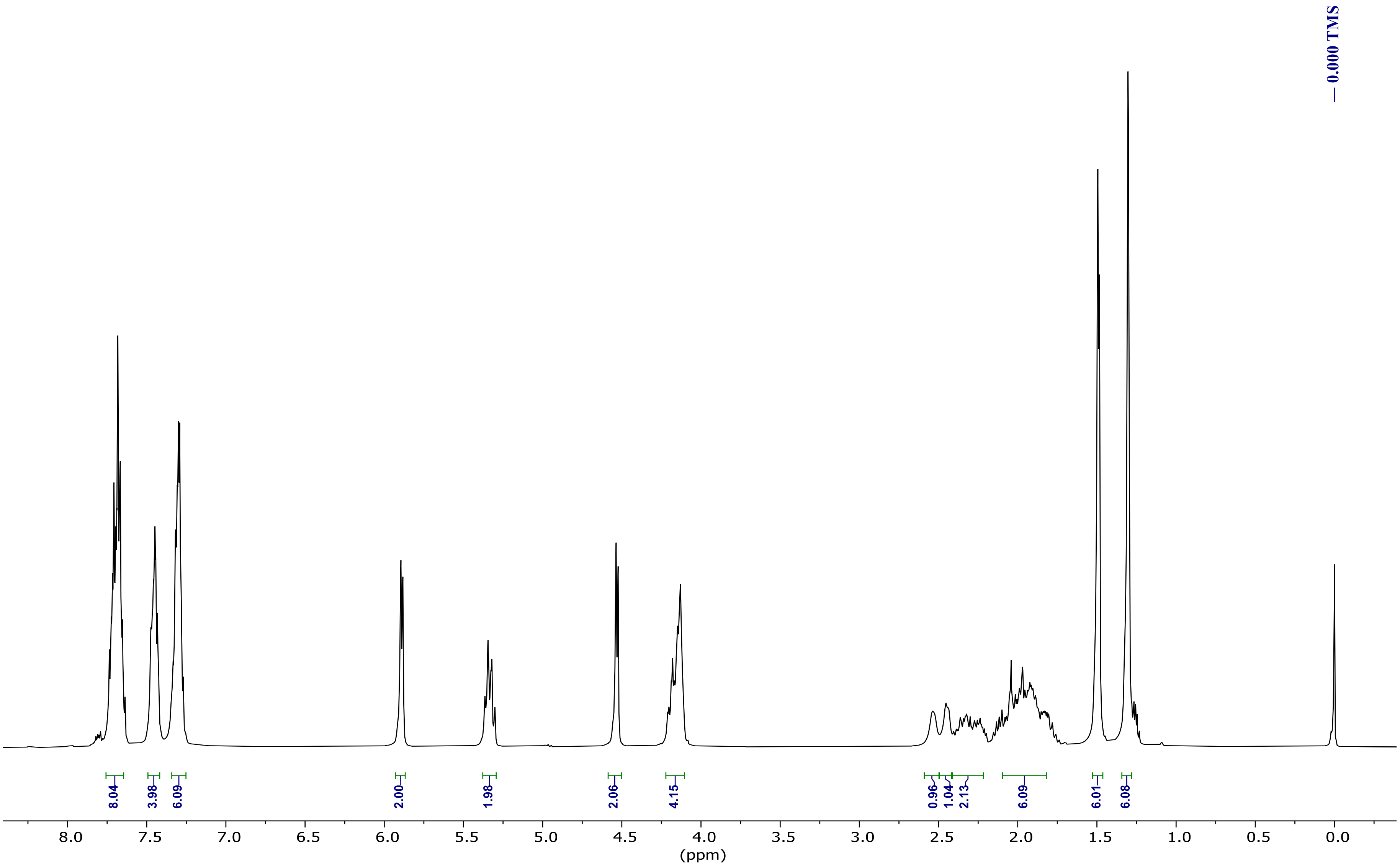

*N-alkoxyphthalimide (**S8**): ^{1}H-NMR (300 MHz, $CDCl_3$)*

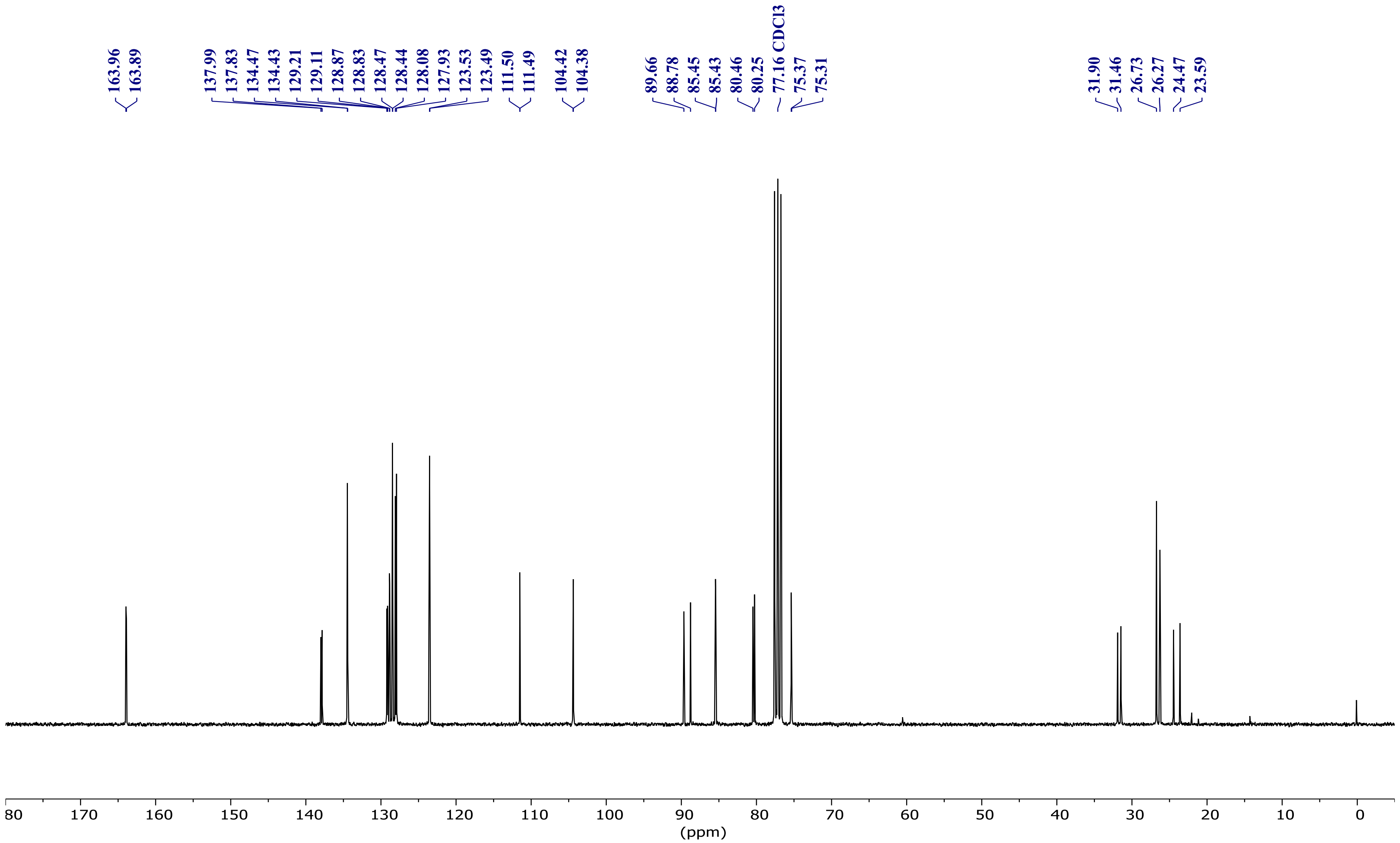

*N-alkoxyphthalimide (**S8**): ^{13}C-NMR (75 MHz, $CDCl_3$)*

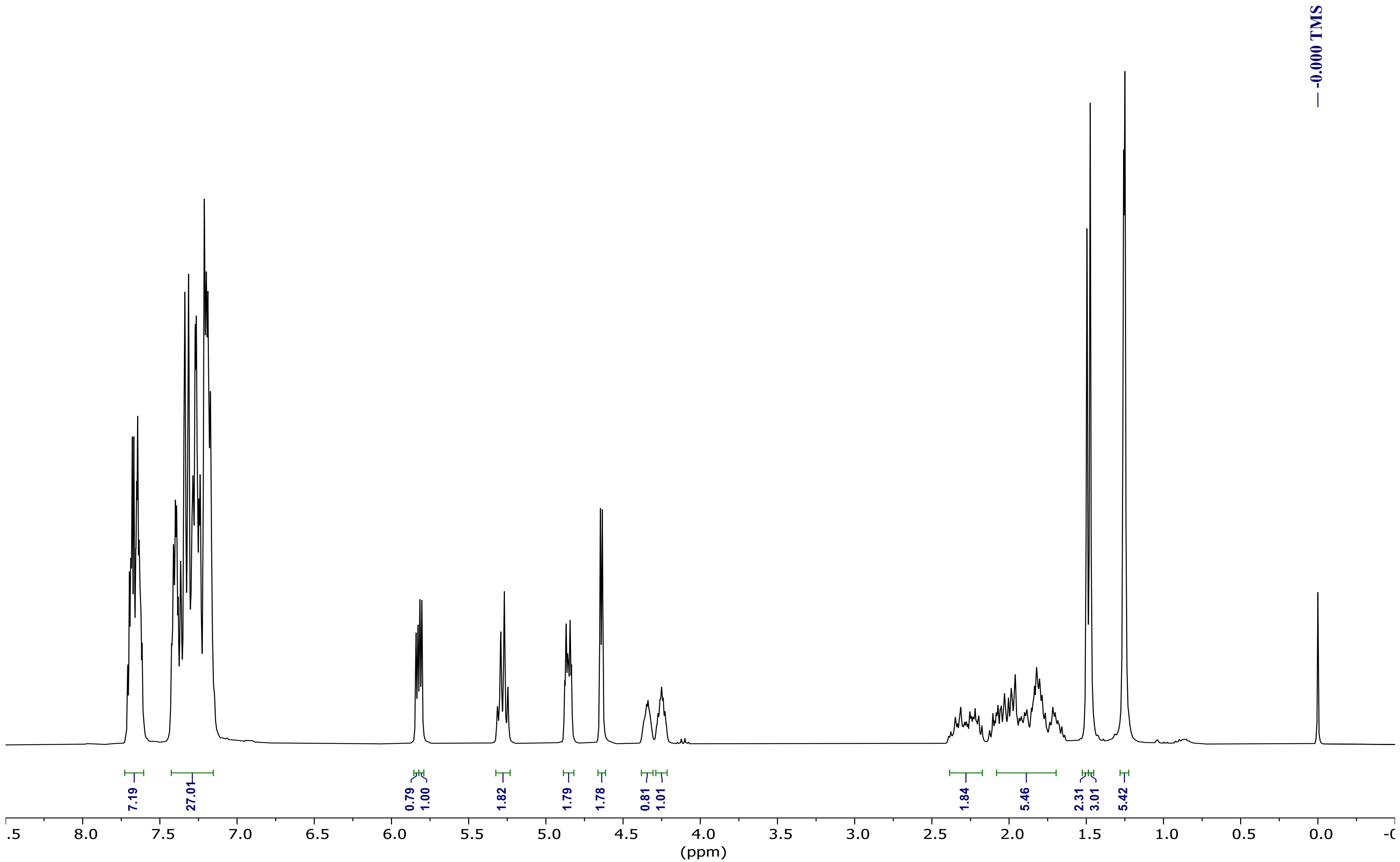

*N-alkoxyphthalimide (**4**): ^{1}H-NMR (300 MHz, $CDCl_3$)*

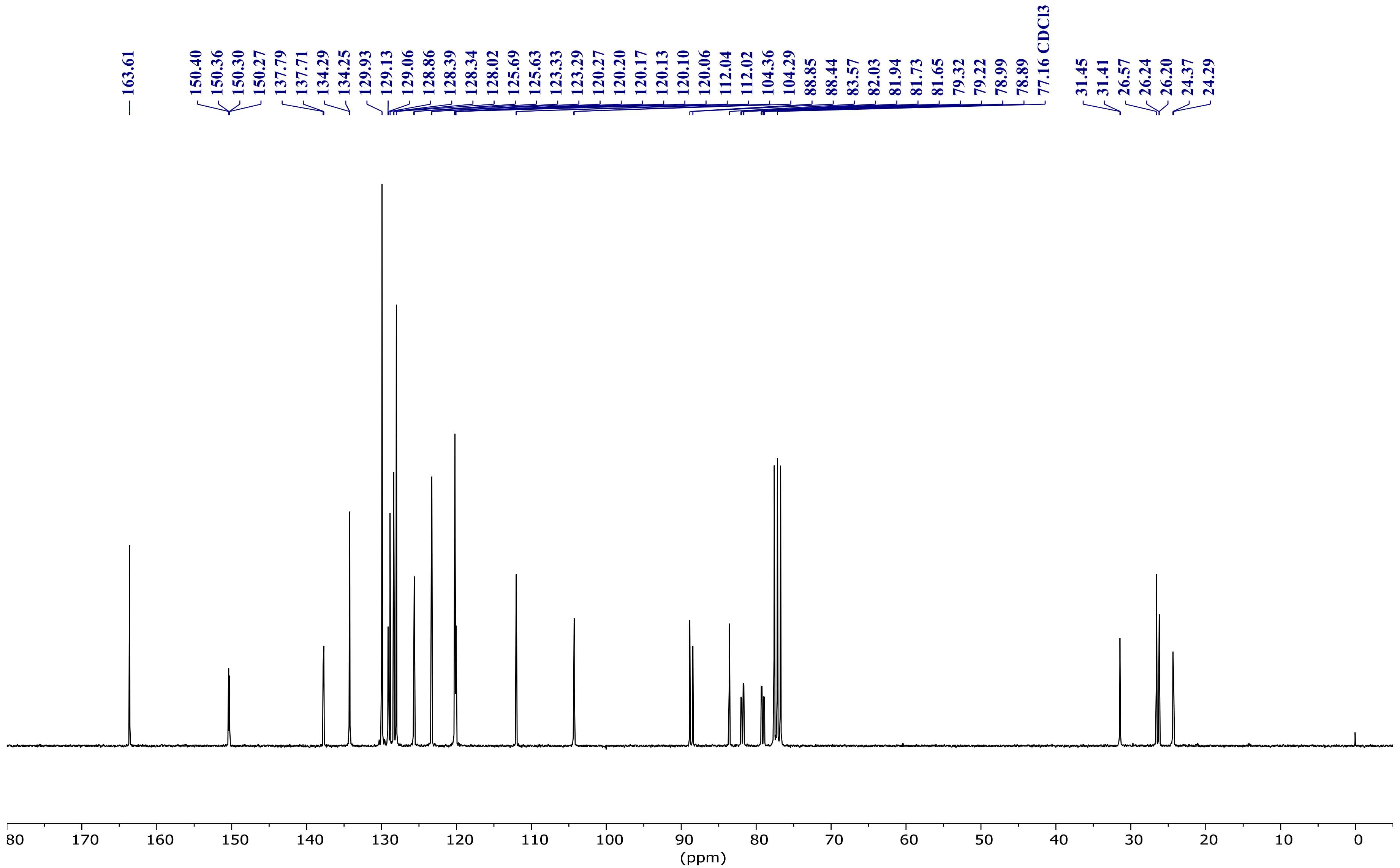

*N-alkoxyphthalimide (**4**): ^{13}C-NMR (75 MHz, $CDCl_3$)*

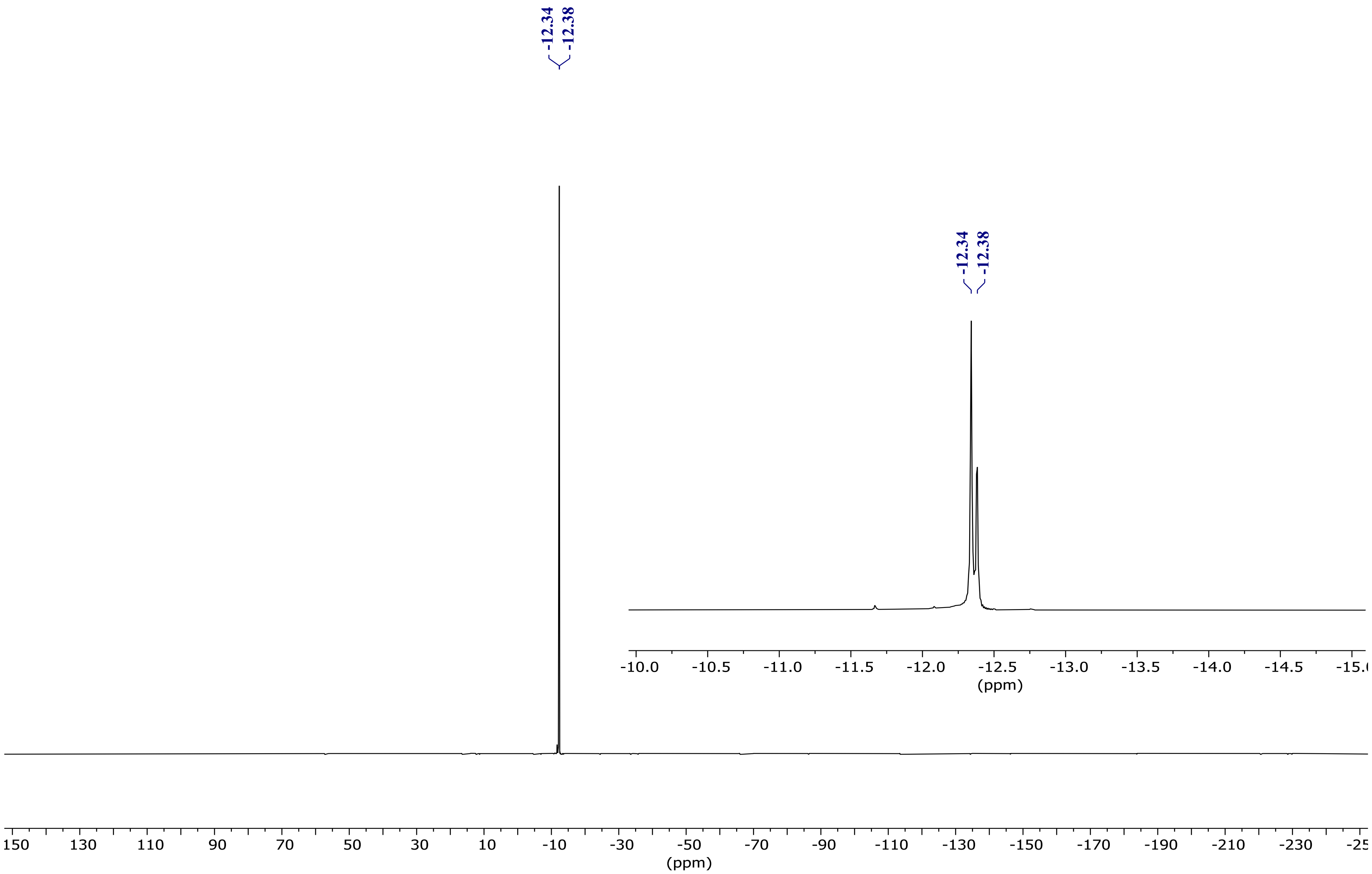

N-alkoxyphthalimide (**4**): $^{31}P\{^{1}H\}$-NMR (202 MHz, $CDCl_3$)

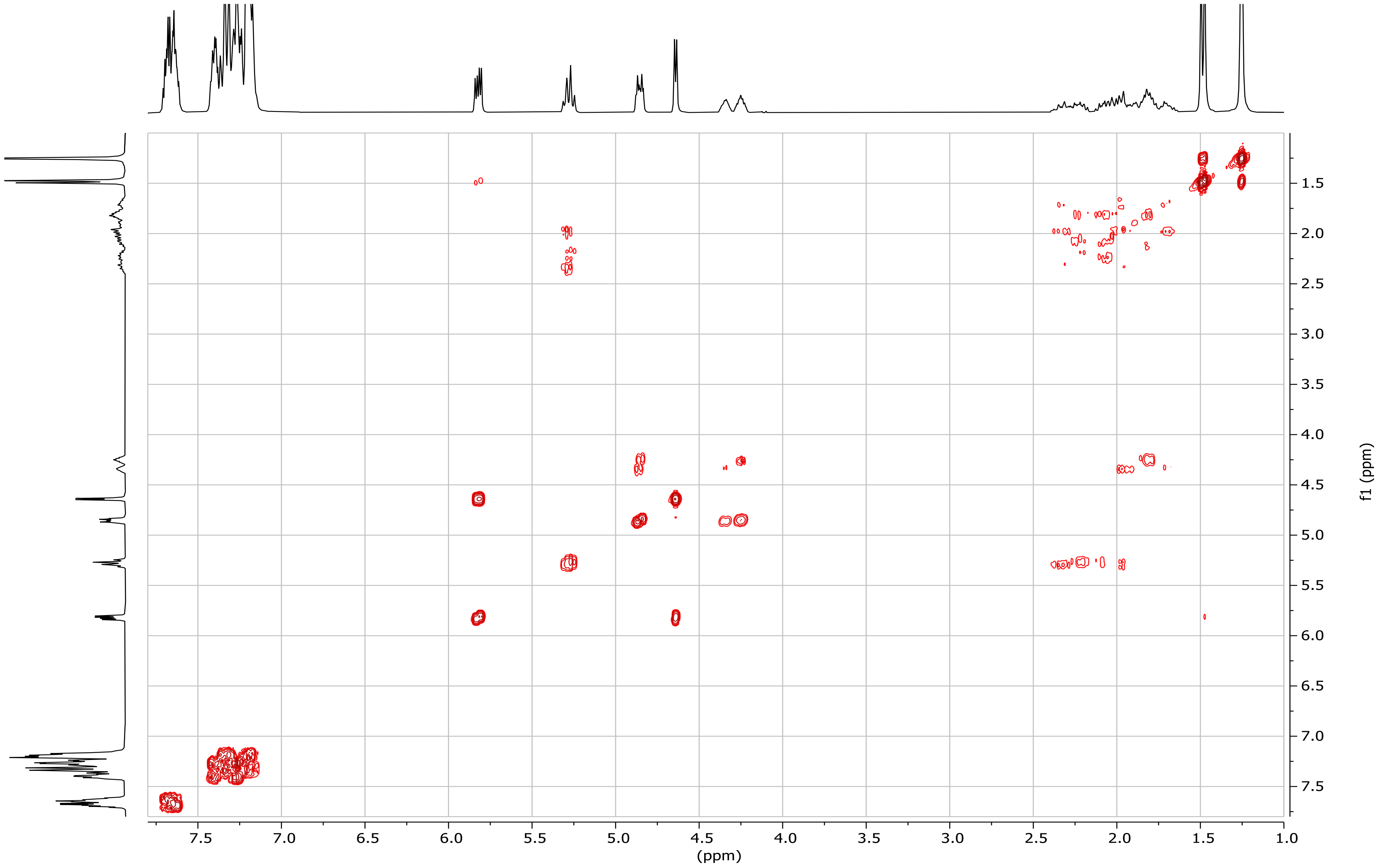

N-alkoxyphthalimide (**4**): COSY ($CDCl_3$)

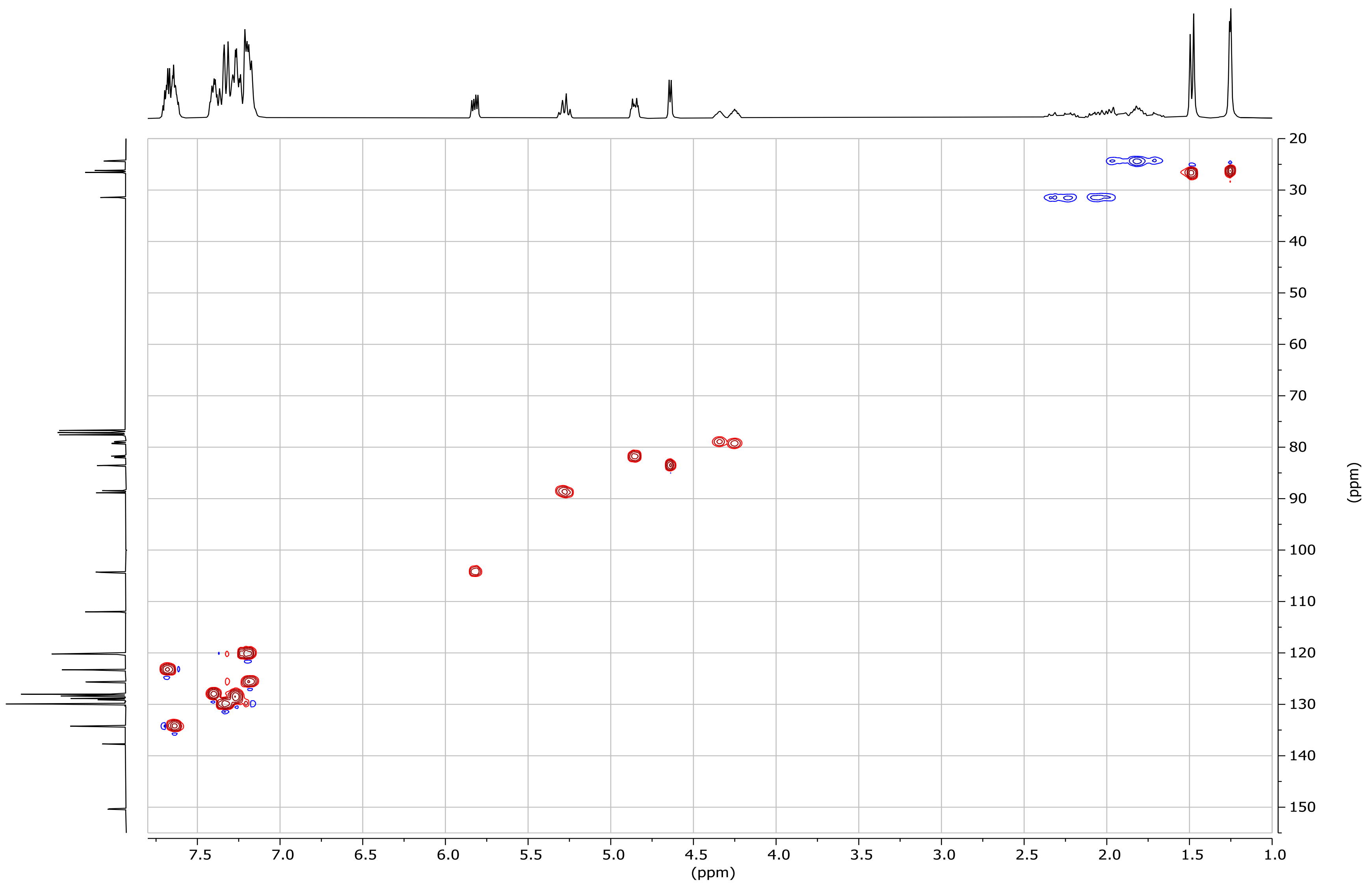

*N-alkoxyphthalimide (**4**): HSQC ($CDCl_3$)*

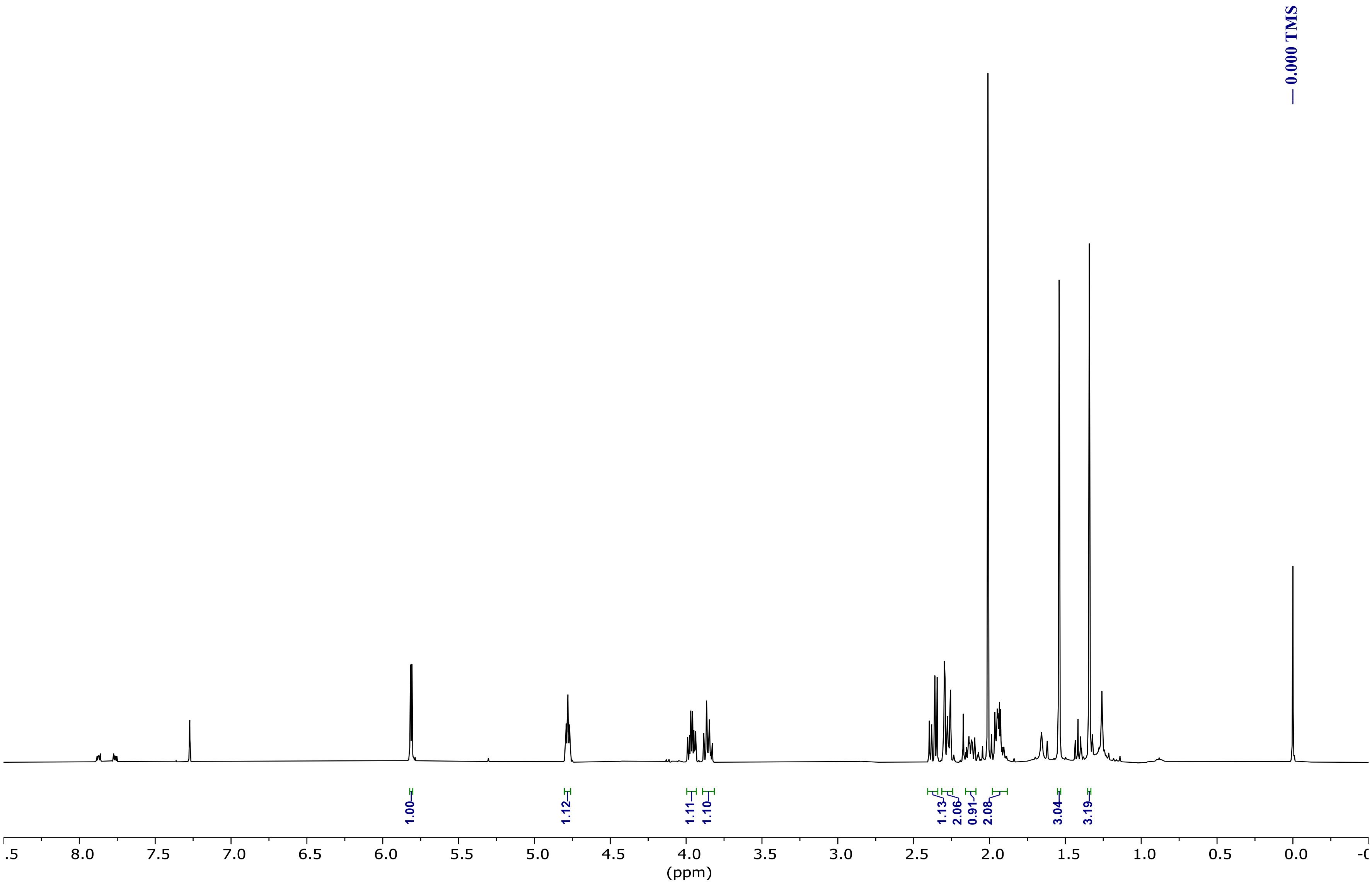

(2S,3a'S,6a'R)-2',2'-dimethyltetrahydro-3H,6'H-spiro[furan-2,5'-furo[2,3-d][1,3]dioxole] (**3**): ^{1}H-NMR (400 MHz, $CDCl_3$)

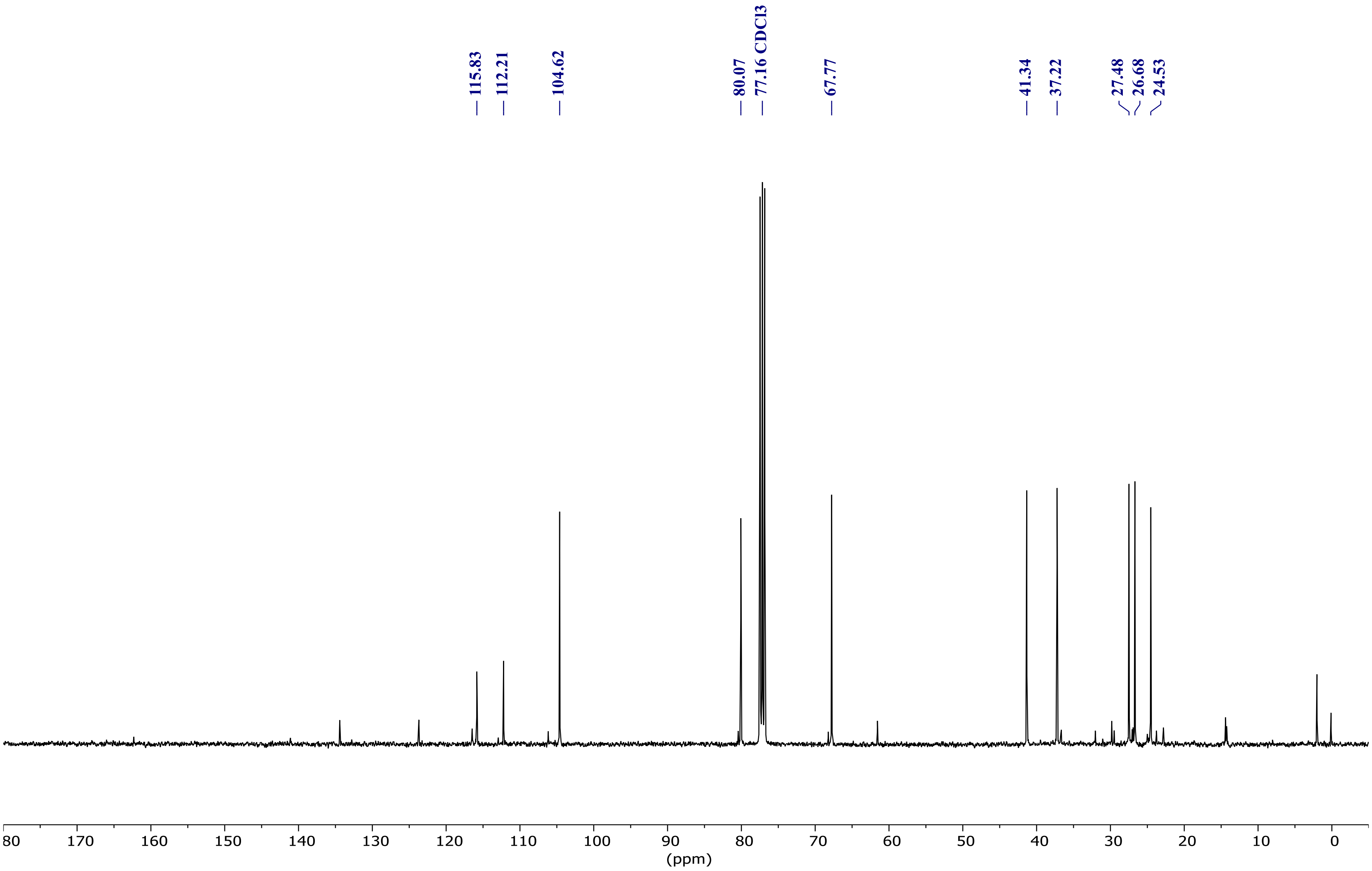

(2S,3a'S,6a'R)-2',2'-dimethyltetrahydro-3H,6'H-spiro[furan-2,5'-furo[2,3-d][1,3]dioxole] (**3**): ^{13}C-NMR (100 MHz, $CDCl_3$)

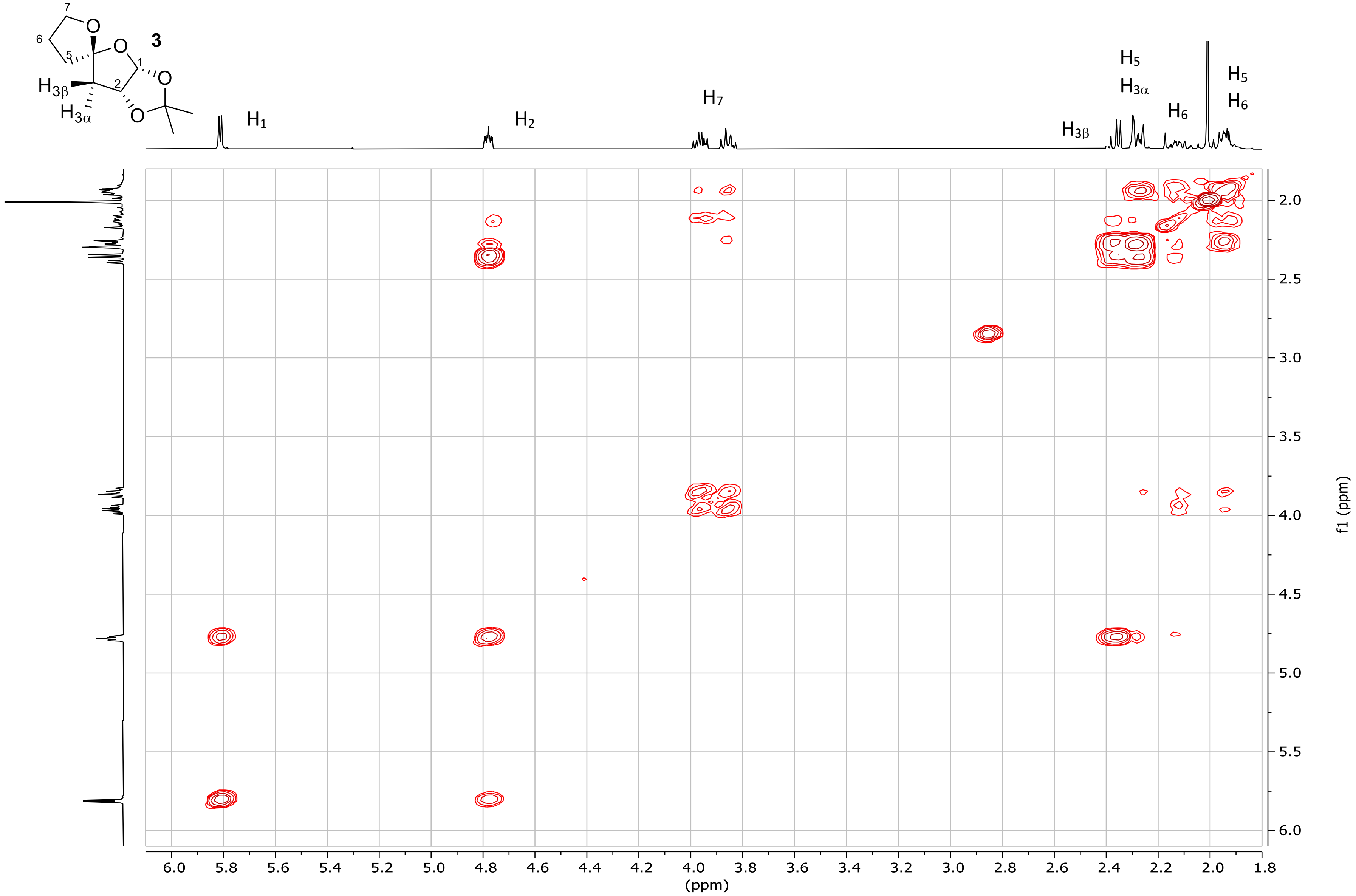

(2S,3a'S,6a'R)-2',2'-dimethyltetrahydro-3H,6'H-spiro[furan-2,5'-furo[2,3-d][1,3]dioxole] (**3**)*: COSY* ($CDCl_3$)

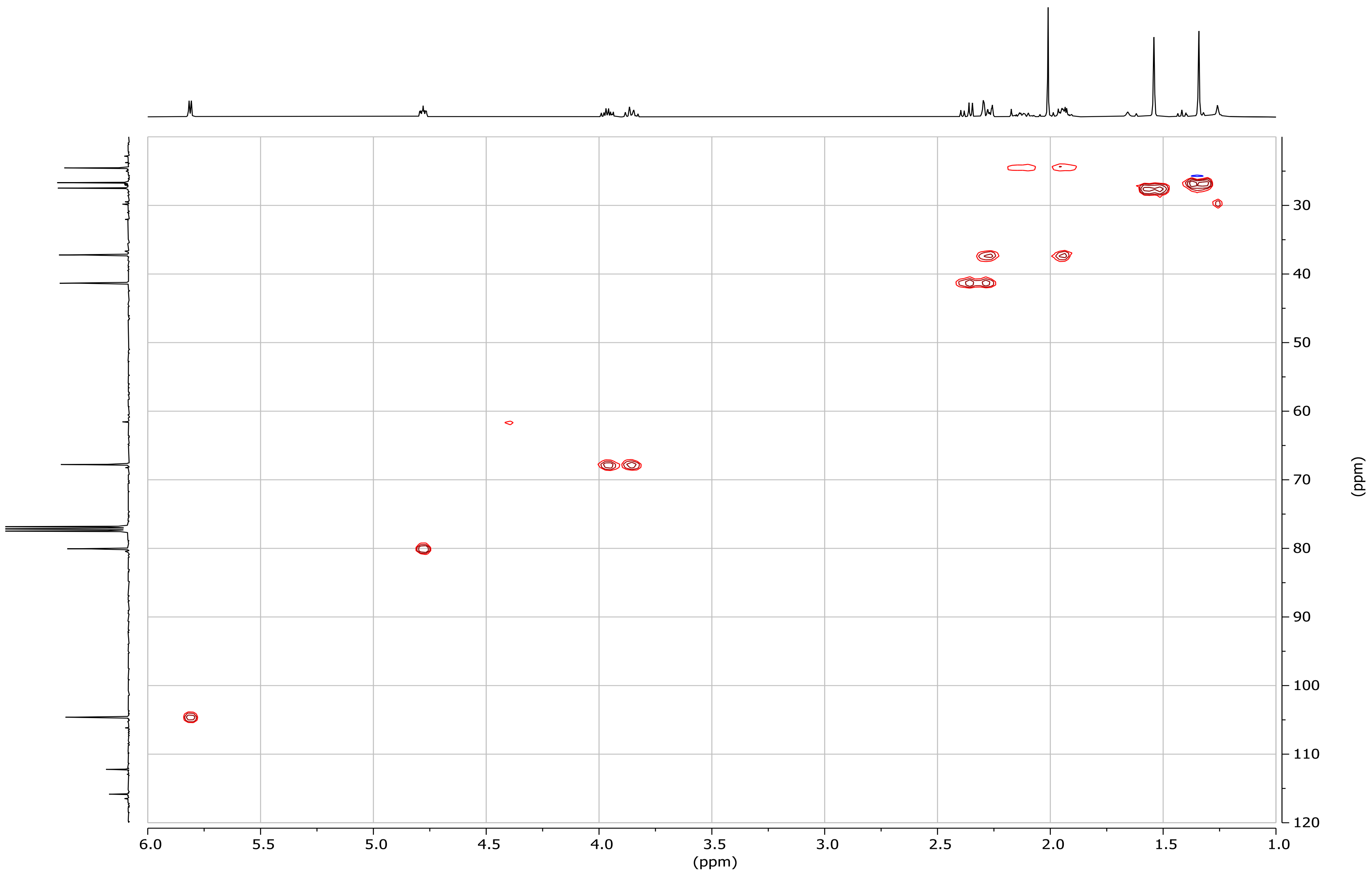

(2S,3a'S,6a'R)-2',2'-dimethyltetrahydro-3H,6'H-spiro[furan-2,5'-furo[2,3-d][1,3]dioxole] (**3**)*: HSQC* ($CDCl_3$)

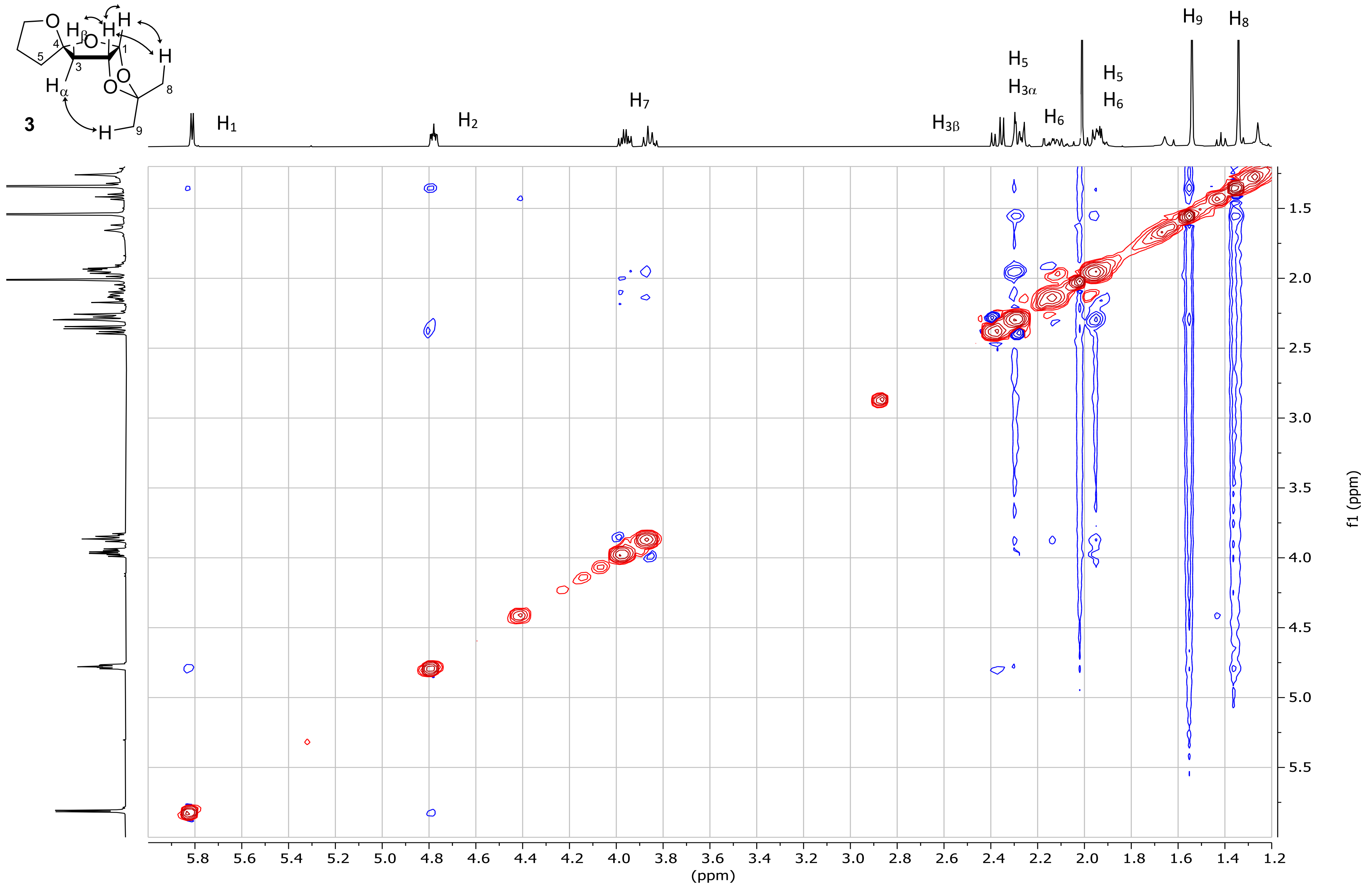

(2S,3a'S,6a'R)-2',2'-dimethyltetrahydro-3H,6'H-spiro[furan-2,5'-furo[2,3-d][1,3]dioxole] (**3**)*: NOESY* ($CDCl_3$)

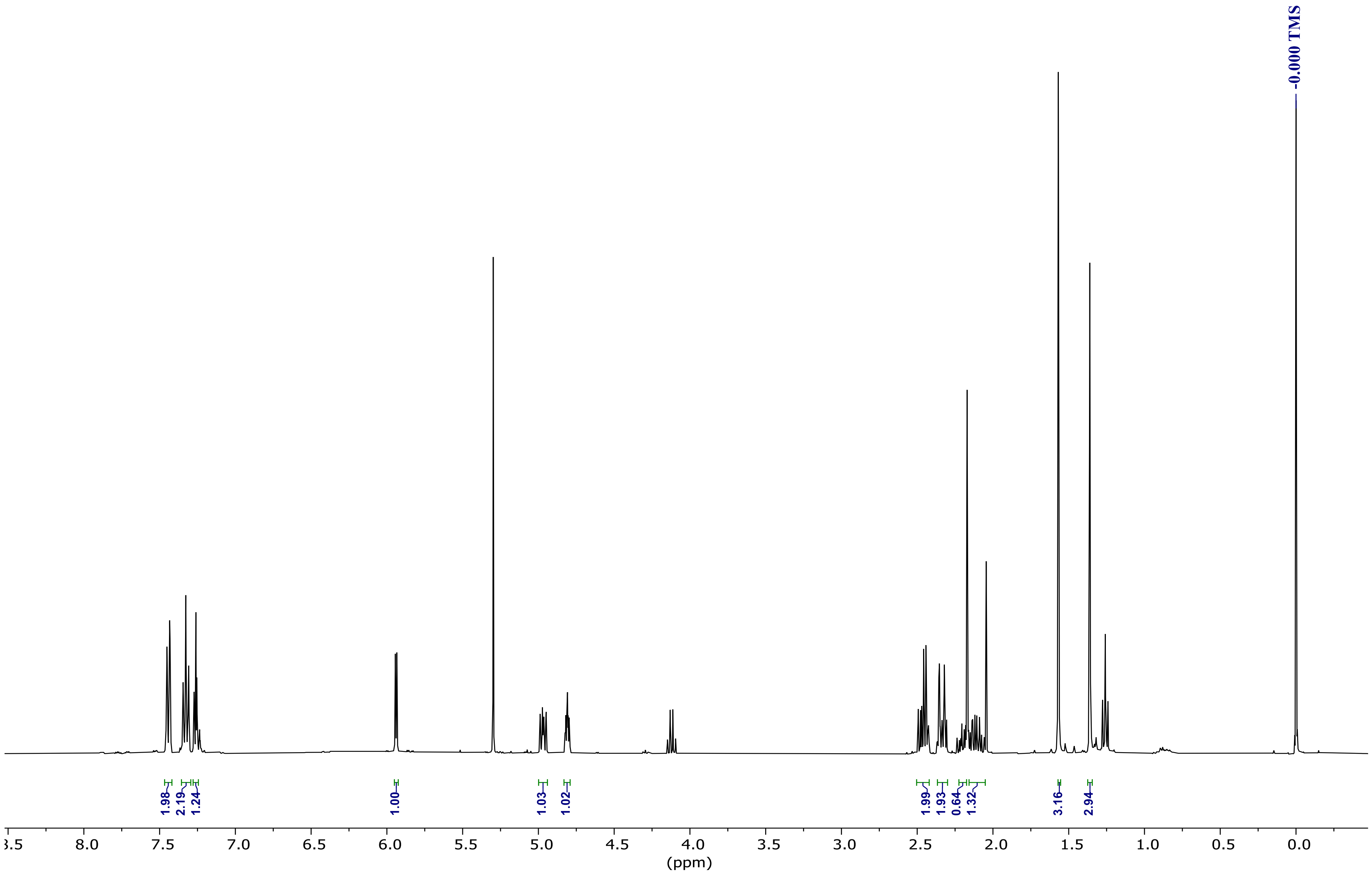

(2S,3a'S,6a'R)-2',2'-dimethyl-5-phenyltetrahydro-3H,6'H-spiro[furan-2,5'-furo[2,3-d][1,3]dioxole] (**5a**): ^{1}H-NMR (400 MHz, $CDCl_3$)

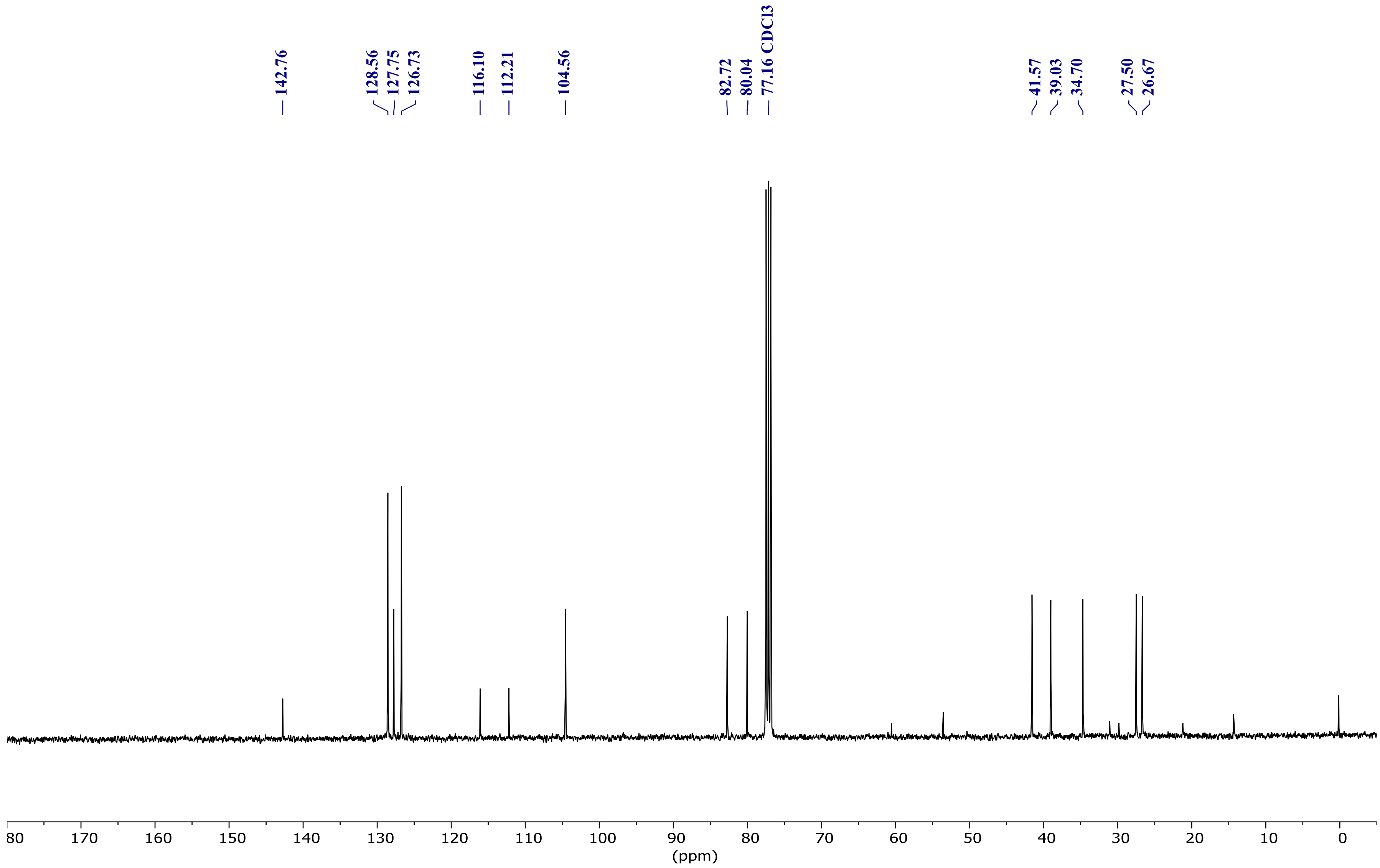

(2S,3a'S,6a'R)-2',2'-dimethyl-5-phenyltetrahydro-3H,6'H-spiro[furan-2,5'-furo[2,3-d][1,3]dioxole] (**5a**): ^{13}C-NMR (100 MHz, $CDCl_3$)

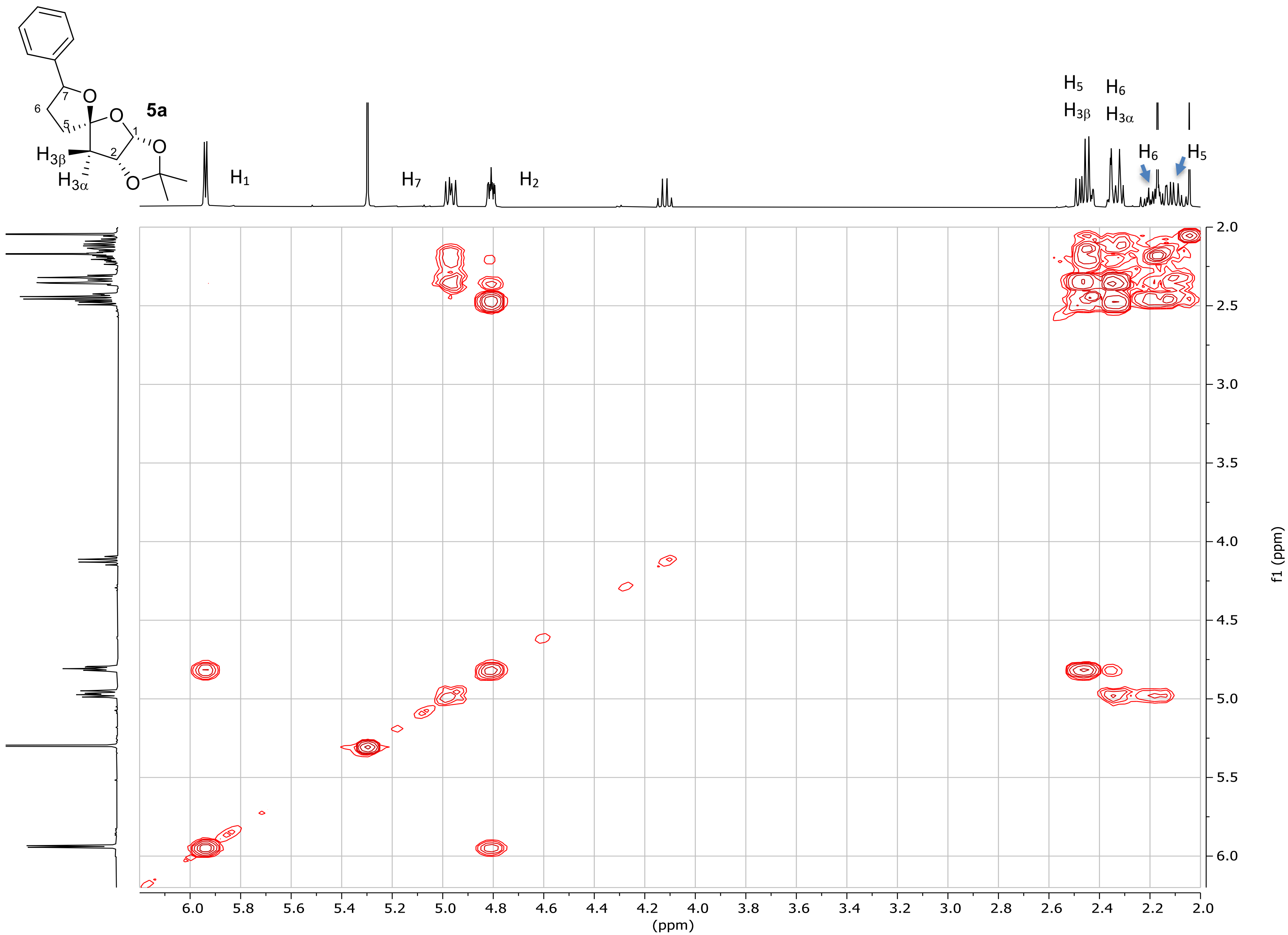

(2S,3a'S,6a'R)-2',2'-dimethyl-5-phenyltetrahydro-3H,6'H-spiro[furan-2,5'-furo[2,3-d][1,3]dioxole] (**5a**): *COSY* ($CDCl_3$)

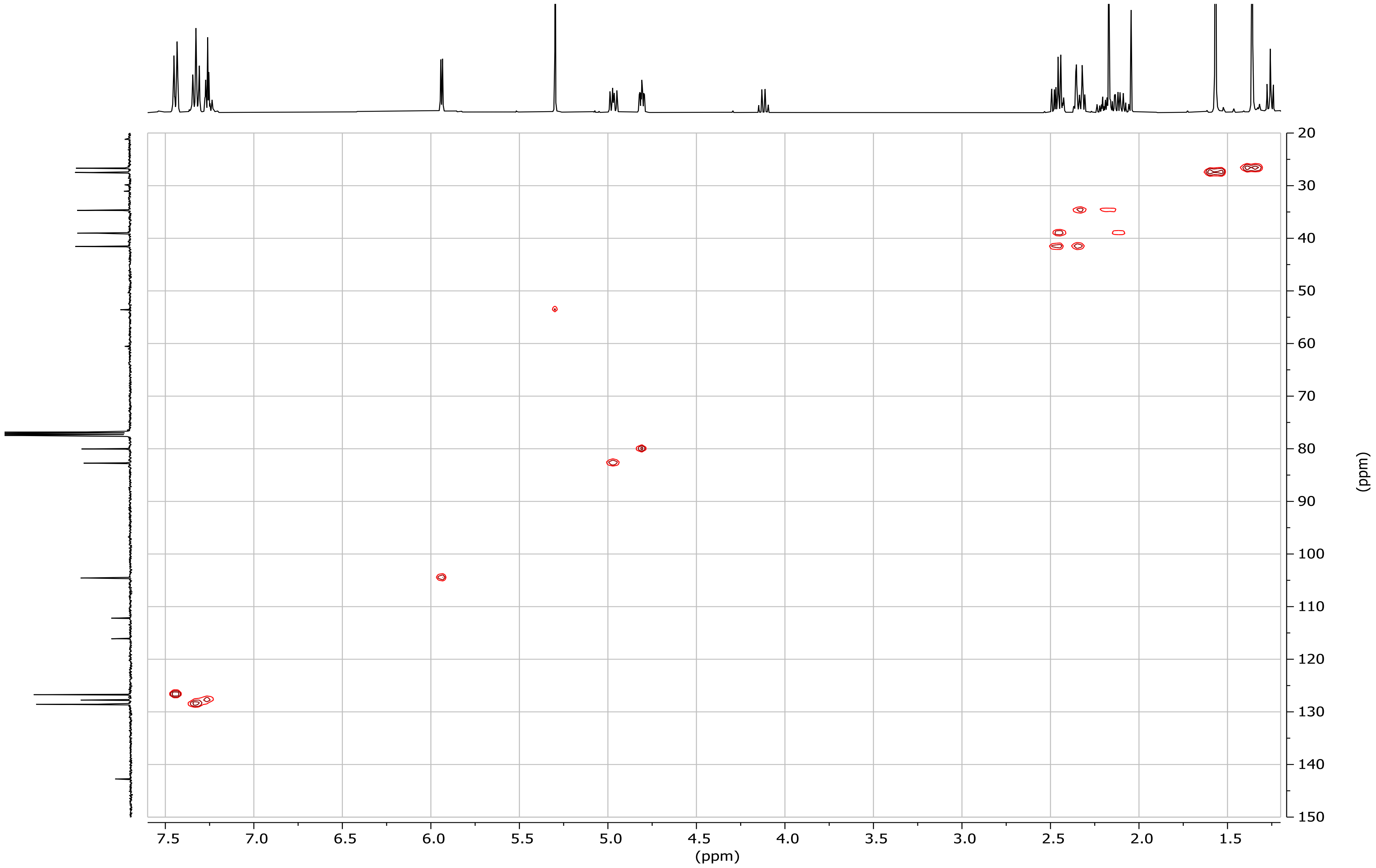

(2S,3a'S,6a'R)-2',2'-dimethyl-5-phenyltetrahydro-3H,6'H-spiro[furan-2,5'-furo[2,3-d][1,3]dioxole] (**5a**)*: HSQC* ($CDCl_3$)

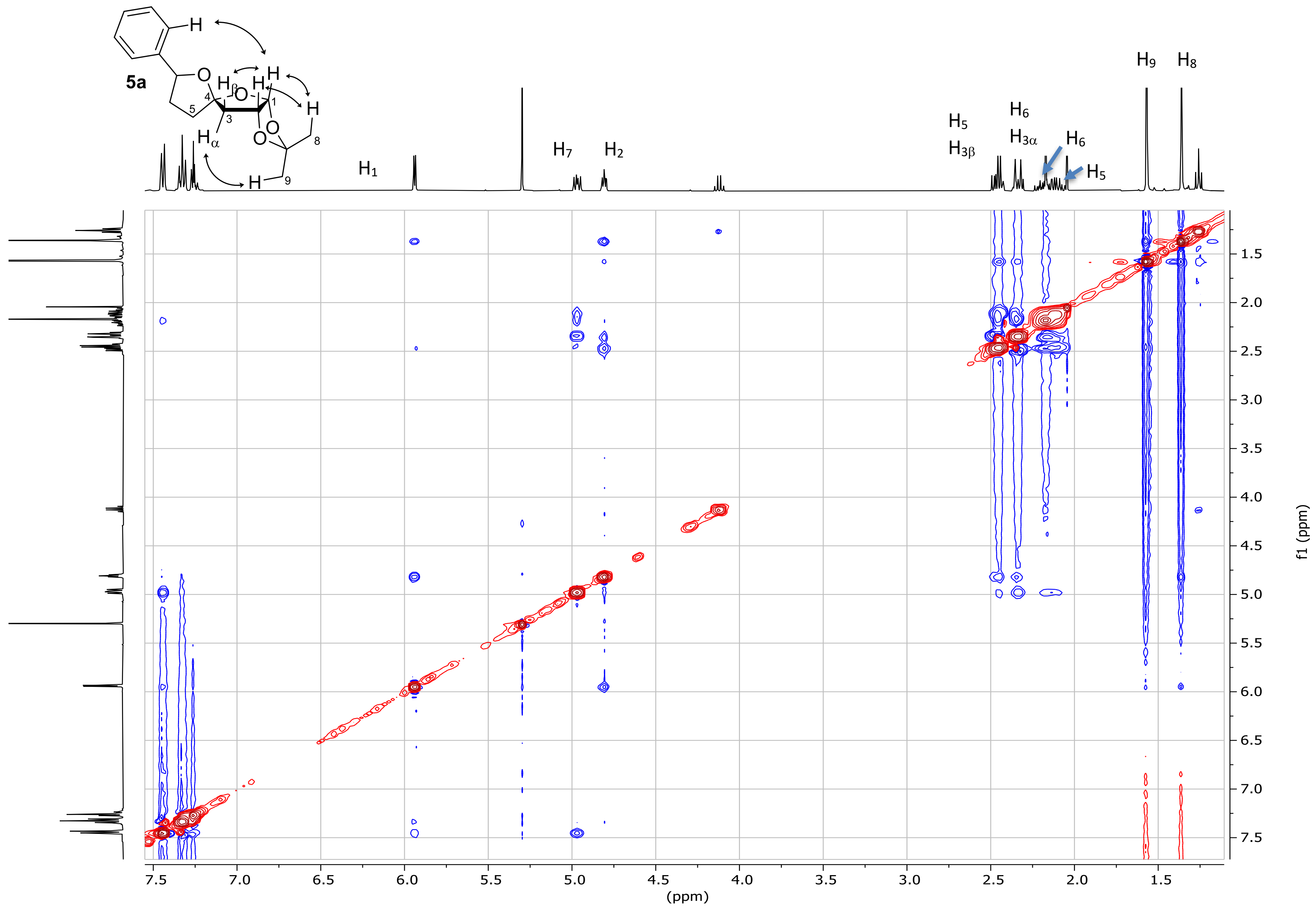

(2S,3a'S,6a'R)-2',2'-dimethyl-5-phenyltetrahydro-3H,6'H-spiro[furan-2,5'-furo[2,3-d][1,3]dioxole] (**5a**): *NOESY* ($CDCl_3$)

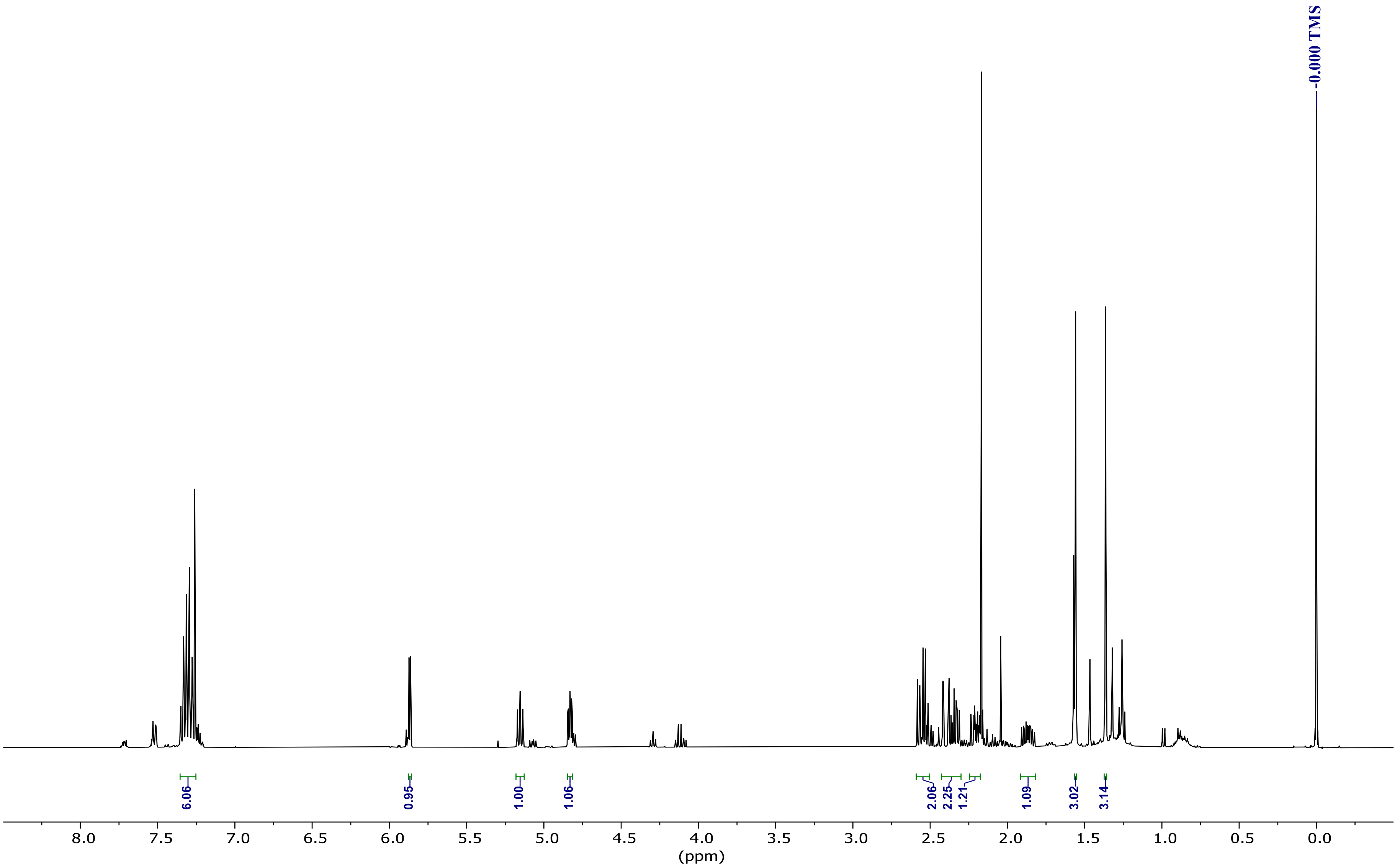

*(2S,3a'S,6a'R)-2',2'-dimethyl-5-phenyltetrahydro-3H,6'H-spiro[furan-2,5'-furo[2,3-d][1,3]dioxole] (**5b**): ^{1}H-NMR (400 MHz, $CDCl_3$)*

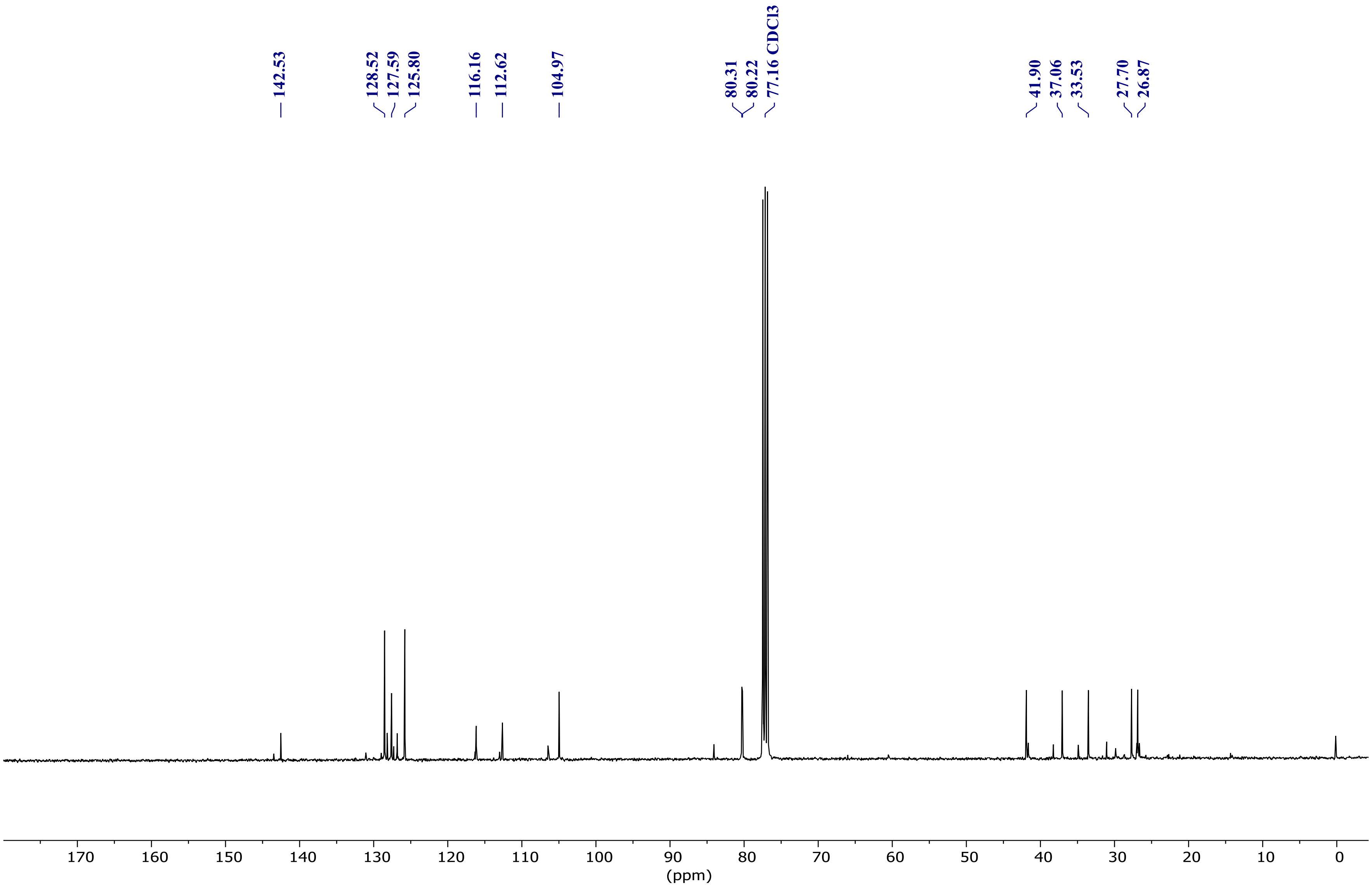

(2S,3a'S,6a'R)-2',2'-dimethyl-5-phenyltetrahydro-3H,6'H-spiro[furan-2,5'-furo[2,3-d][1,3]dioxole] (**5b**): ^{13}C-NMR (100 MHz, $CDCl_3$)

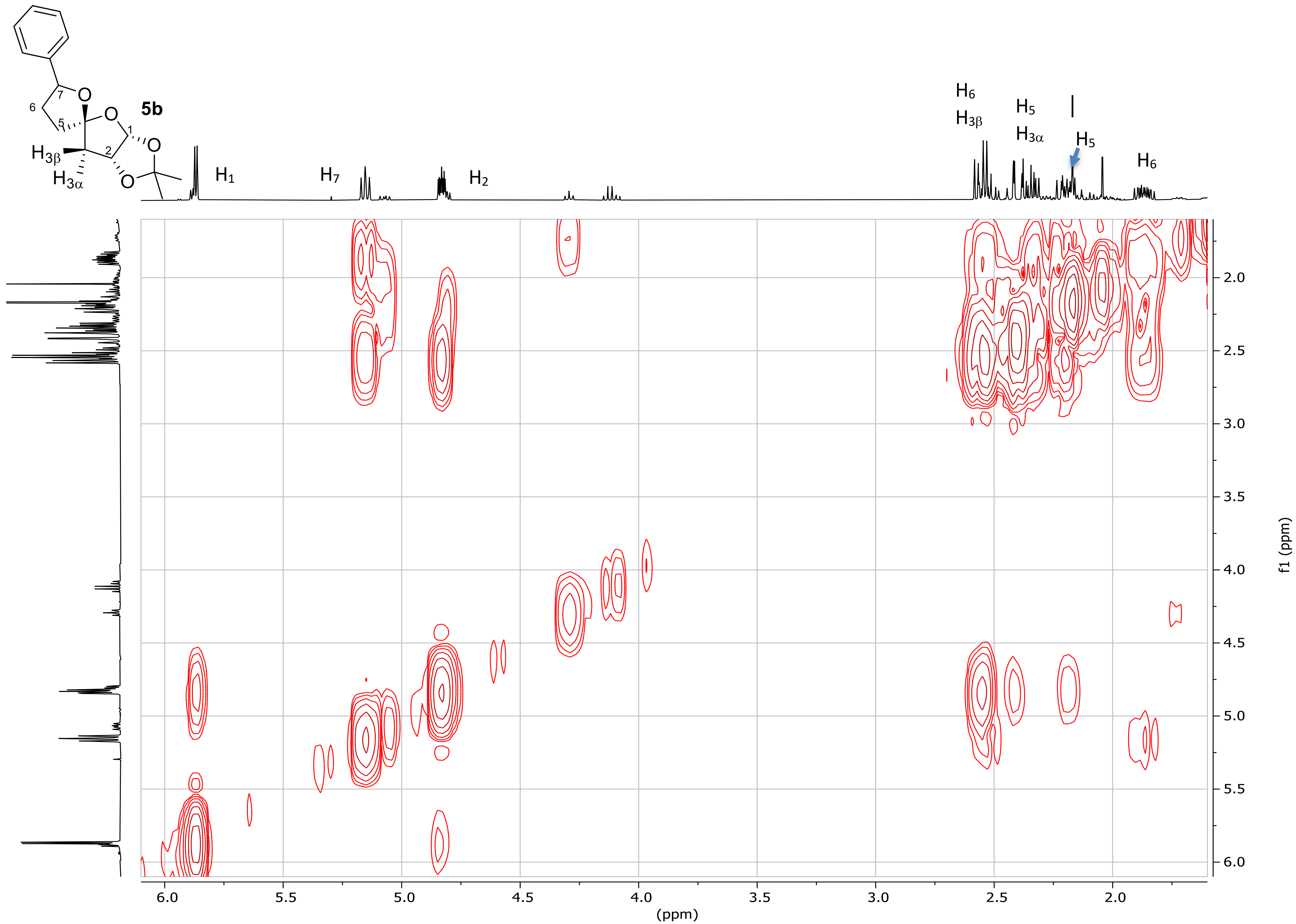

(2S,3a'S,6a'R)-2',2'-dimethyl-5-phenyltetrahydro-3H,6'H-spiro[furan-2,5'-furo[2,3-d][1,3]dioxole] (**5b**): COSY ($CDCl_3$)

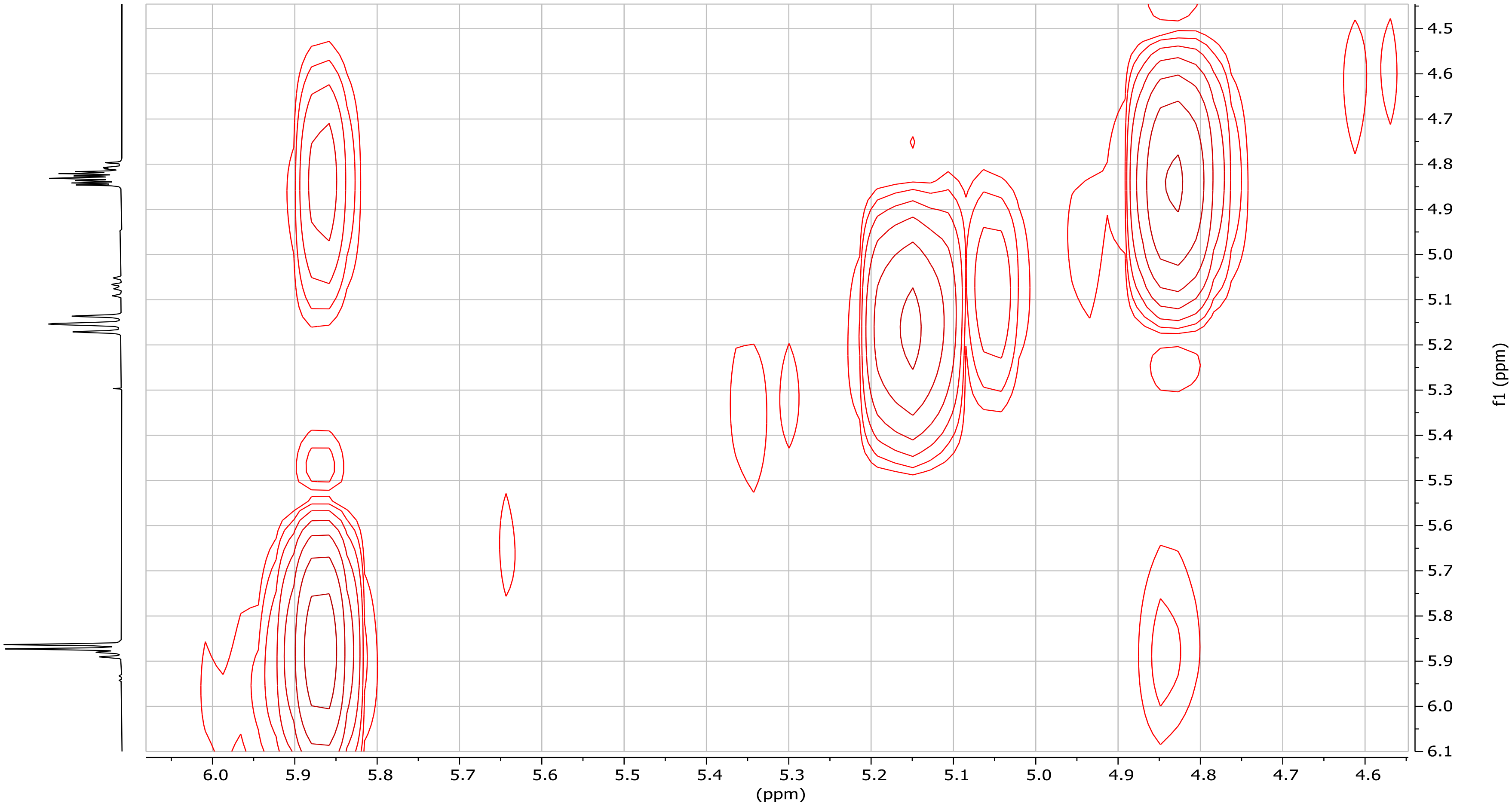

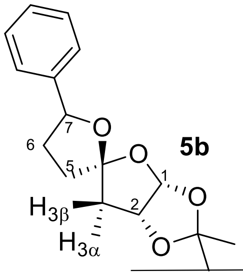

(2S,3a'S,6a'R)-2',2'-dimethyl-5-phenyltetrahydro-3H,6'H-spiro[furan-2,5'-furo[2,3-d][1,3]dioxole] (**5b**)*: COSY (CDCl*$_3$*)*

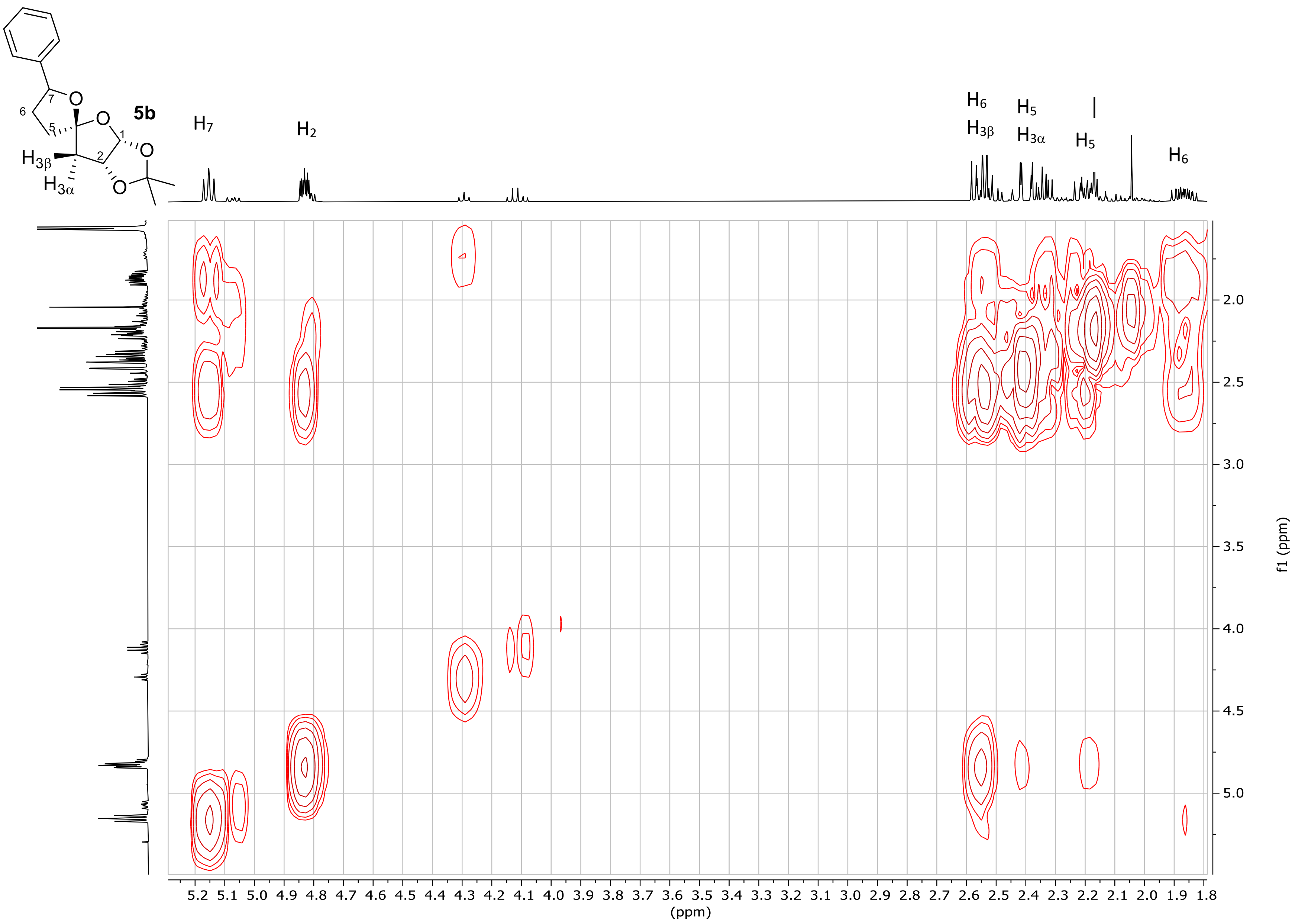

(2S,3a'S,6a'R)-2',2'-dimethyl-5-phenyltetrahydro-3H,6'H-spiro[furan-2,5'-furo[2,3-d][1,3]dioxole] (**5b**)*: COSY* ($CDCl_3$)

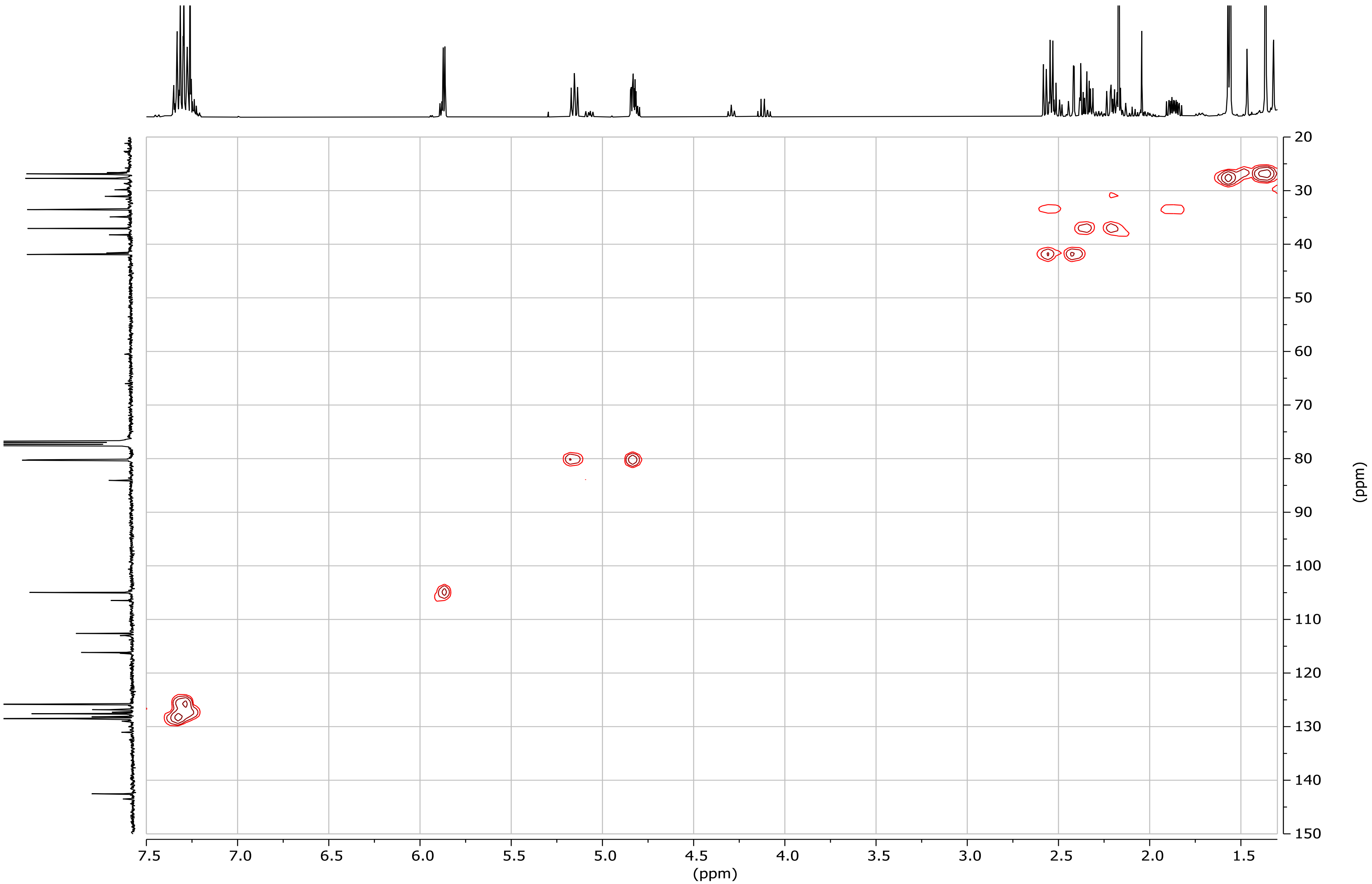

(2S,3a'S,6a'R)-2',2'-dimethyl-5-phenyltetrahydro-3H,6'H-spiro[furan-2,5'-furo[2,3-d][1,3]dioxole] (**5b**)*: HSQC* ($CDCl_3$)

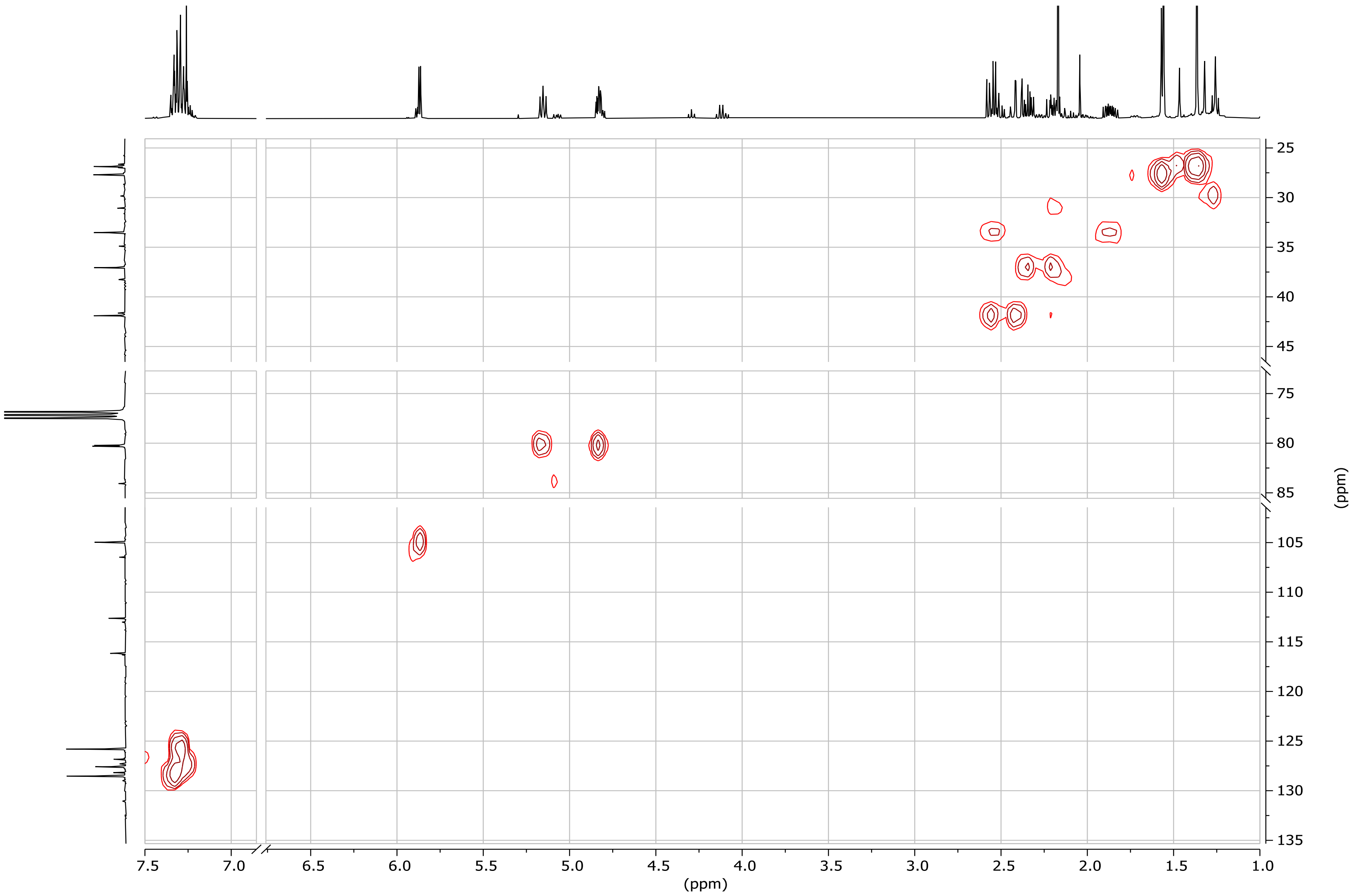

(2S,3a'S,6a'R)-2',2'-dimethyl-5-phenyltetrahydro-3H,6'H-spiro[furan-2,5'-furo[2,3-d][1,3]dioxole] (**5b**)*: HSQC* ($CDCl_3$)

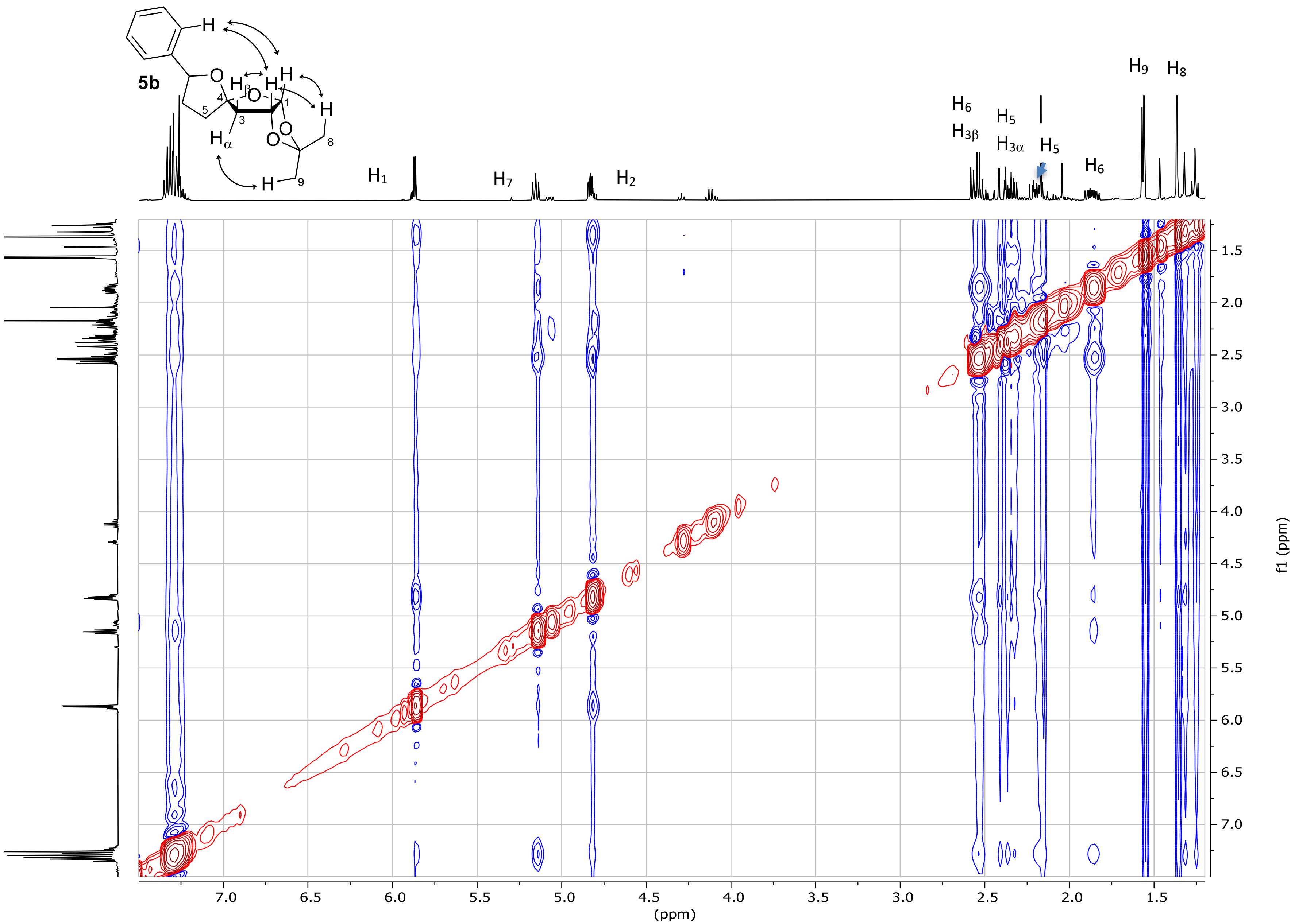

(2S,3a'S,6a'R)-2',2'-dimethyl-5-phenyltetrahydro-3H,6'H-spiro[furan-2,5'-furo[2,3-d][1,3]dioxole] (**5b**)*: NOESY* ($CDCl_3$)

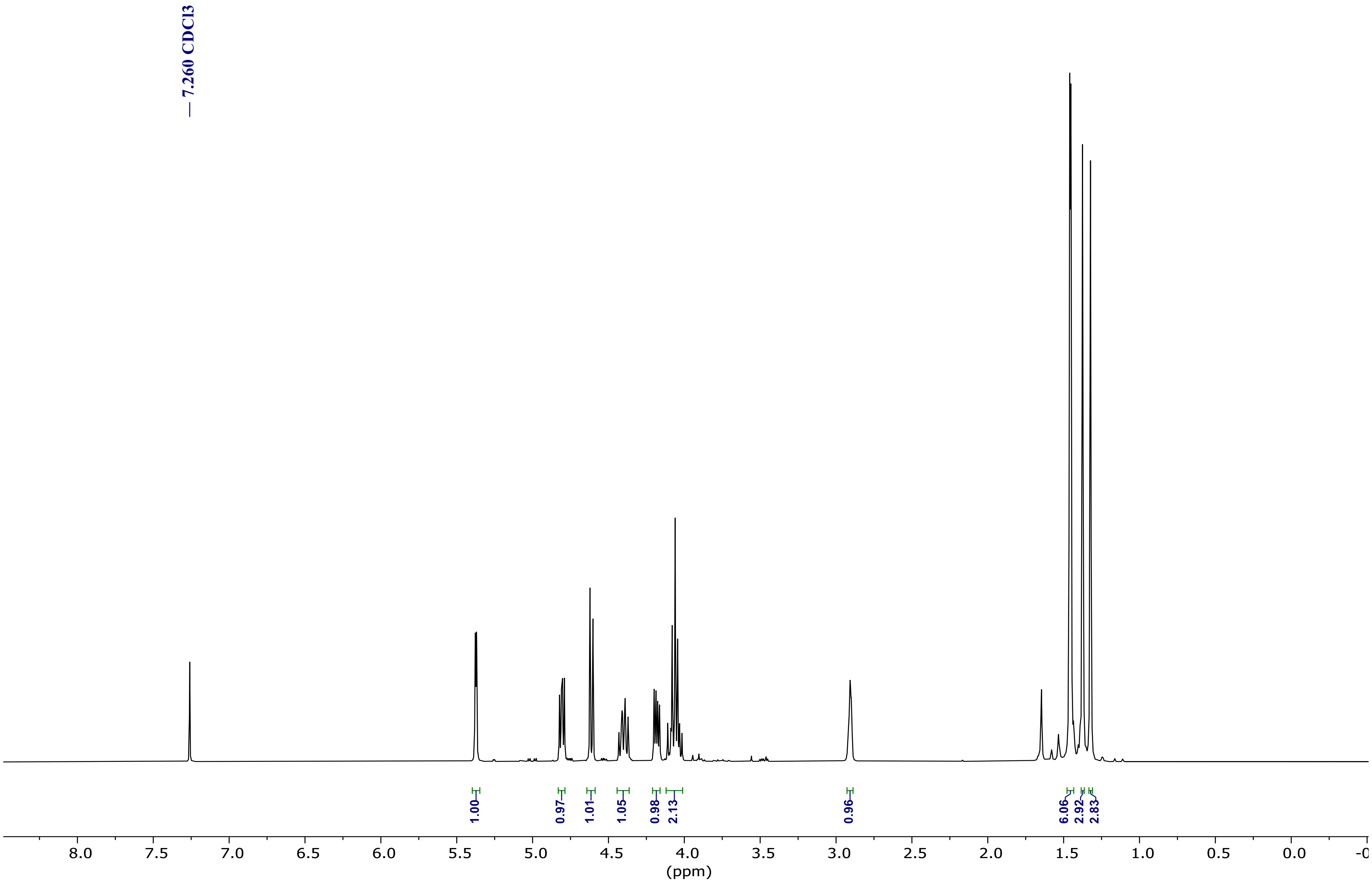

(3aS,6R,6aS)-6-((R)-2,2-dimethyl-1,3-dioxolan-4-yl)-2,2-dimethyltetrahydrofuro[3,4-d][1,3]dioxol-4-ol (**8**): ^{1}H-NMR (300 MHz, $CDCl_3$)

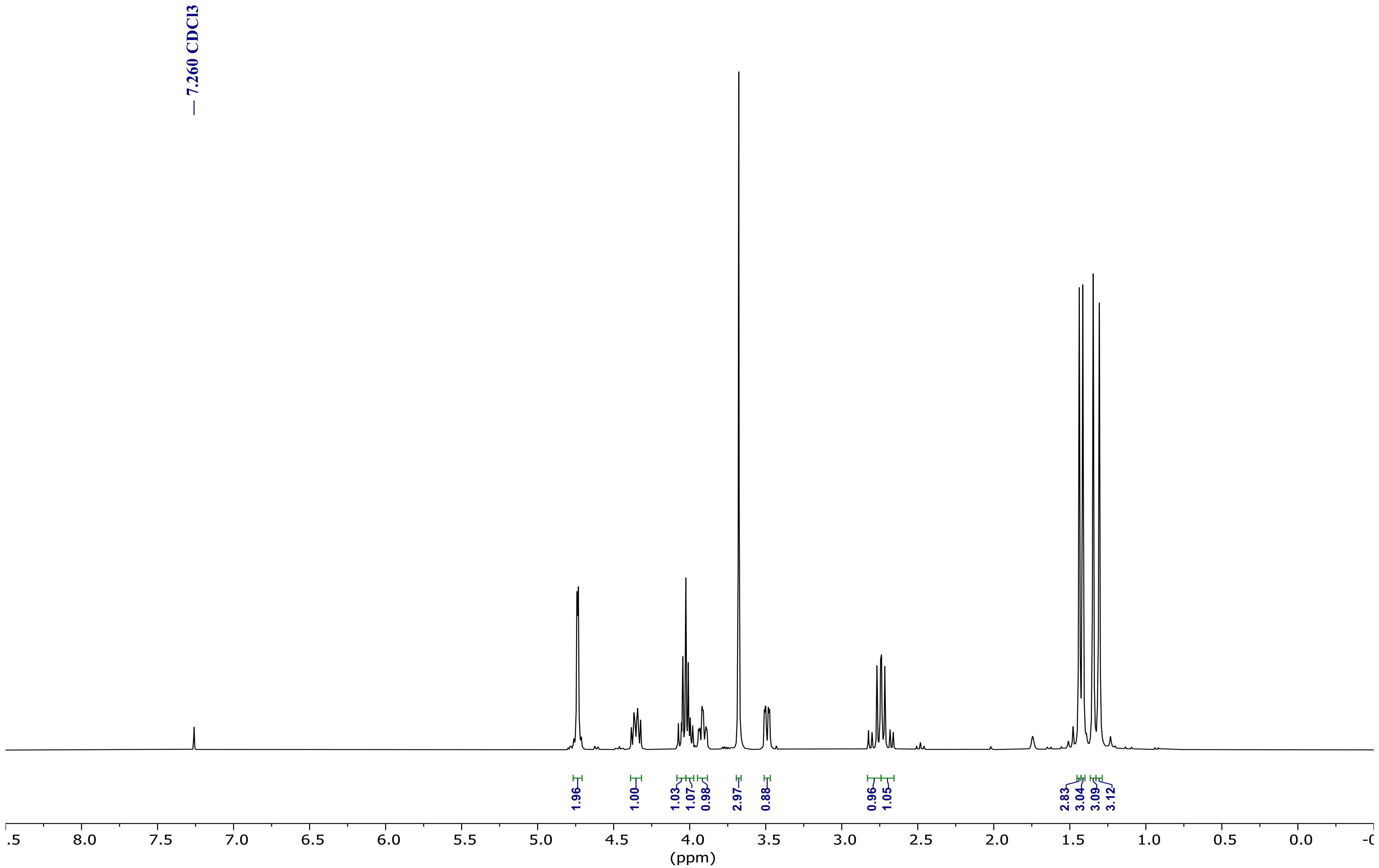

*Methyl 2-((3aR,4S,6R,6aS)-6-((R)-2,2-dimethyl-1,3-dioxolan-4-yl)-2,2-dimethyltetrahydrofuro[3,4-d][1,3]dioxol-4-yl)acetate (**12b**): ^{1}H-NMR (300 MHz, $CDCl_3$)*

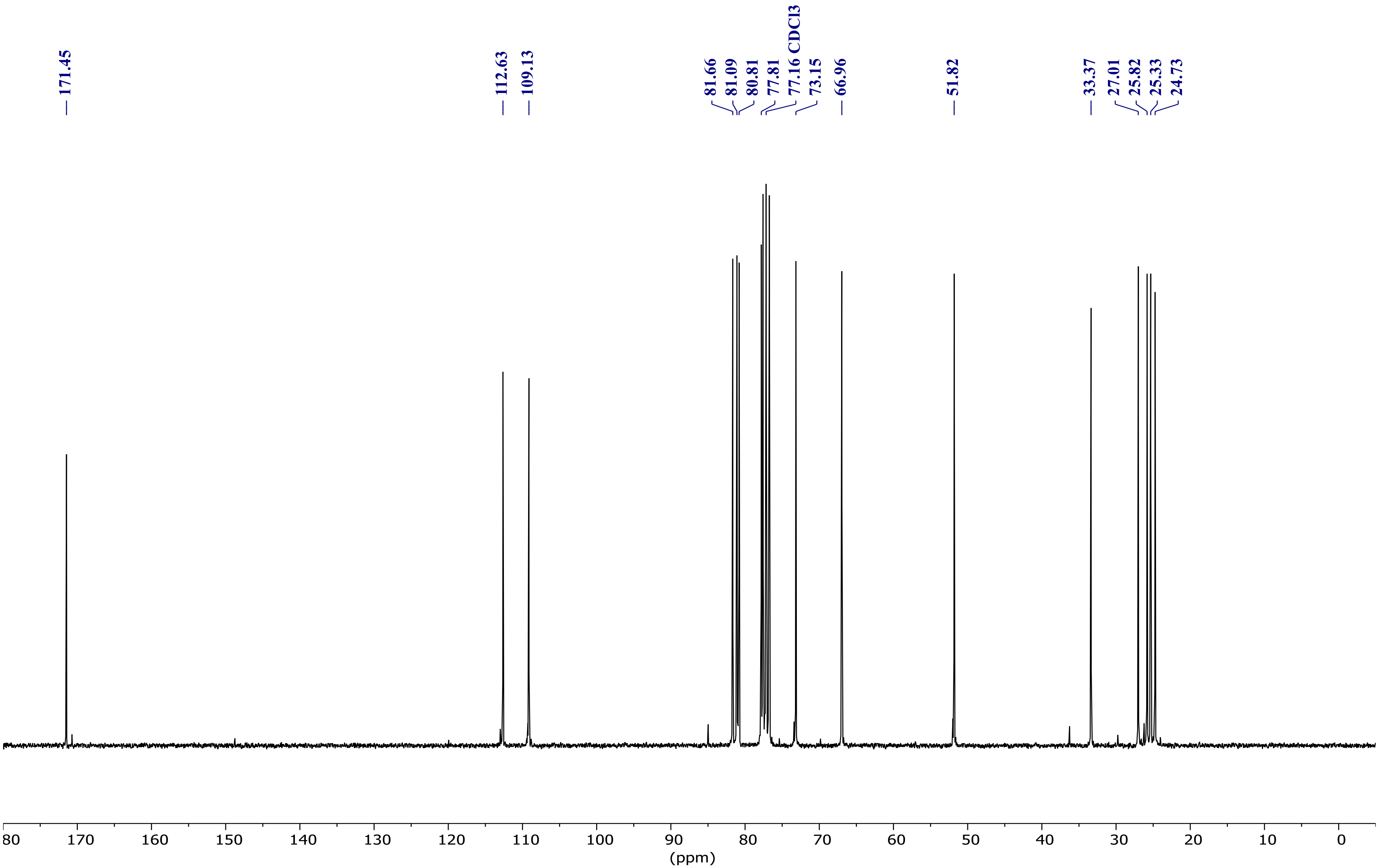

*Methyl 2-((3aR,4S,6R,6aS)-6-((R)-2,2-dimethyl-1,3-dioxolan-4-yl)-2,2-dimethyltetrahydrofuro[3,4-d][1,3]dioxol-4-yl)acetate (**12b**): ^{13}C-NMR (75 MHz, $CDCl_3$)*

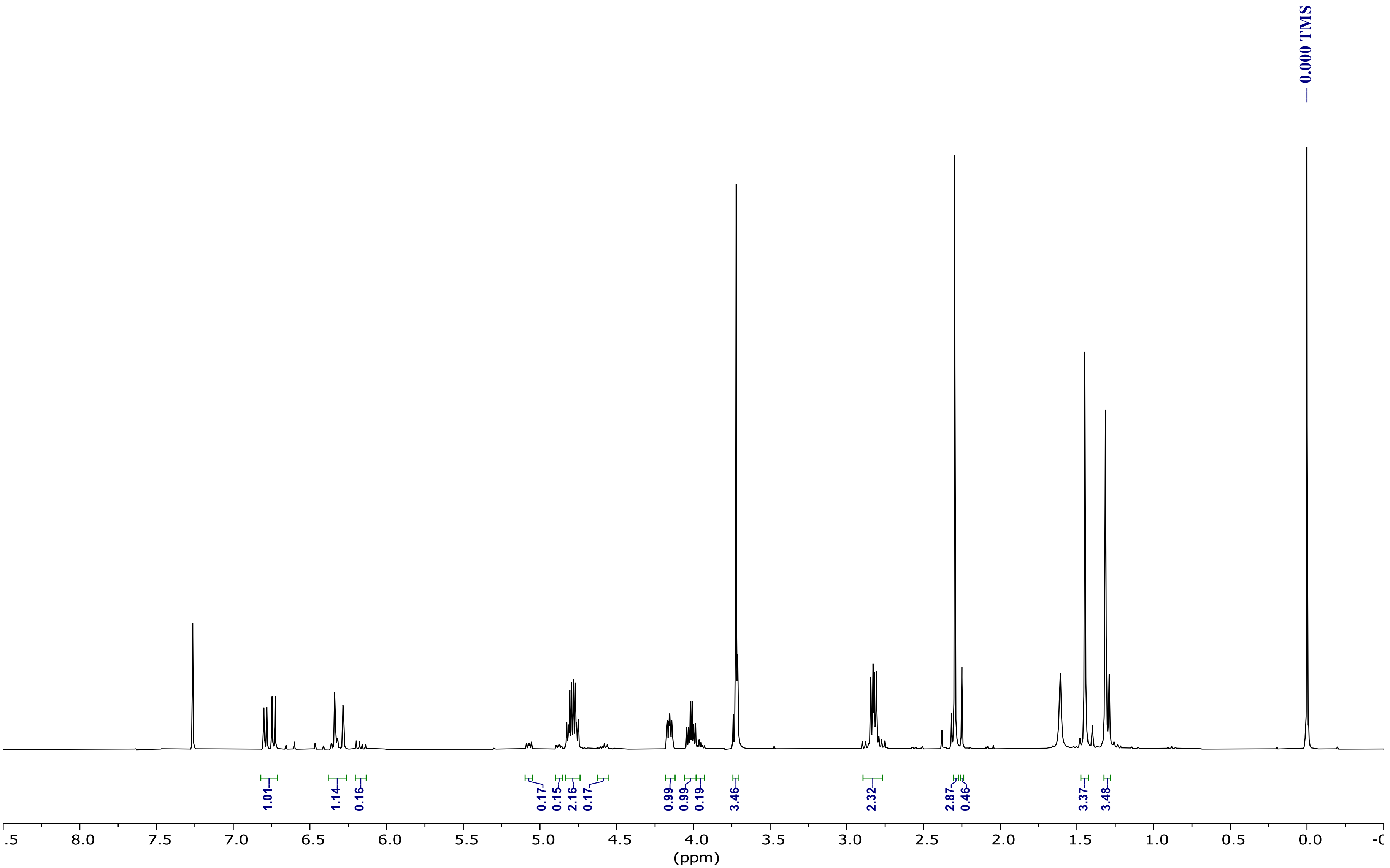

*Methyl 2-((3aR,4S,6R,6aS)-2,2-dimethyl-6-(3-oxobut-1-en-1-yl)tetrahydrofuro[3,4-d][1,3]dioxol-4-yl)acetate (**9**): ^{1}H-NMR (300 MHz, $CDCl_3$)*

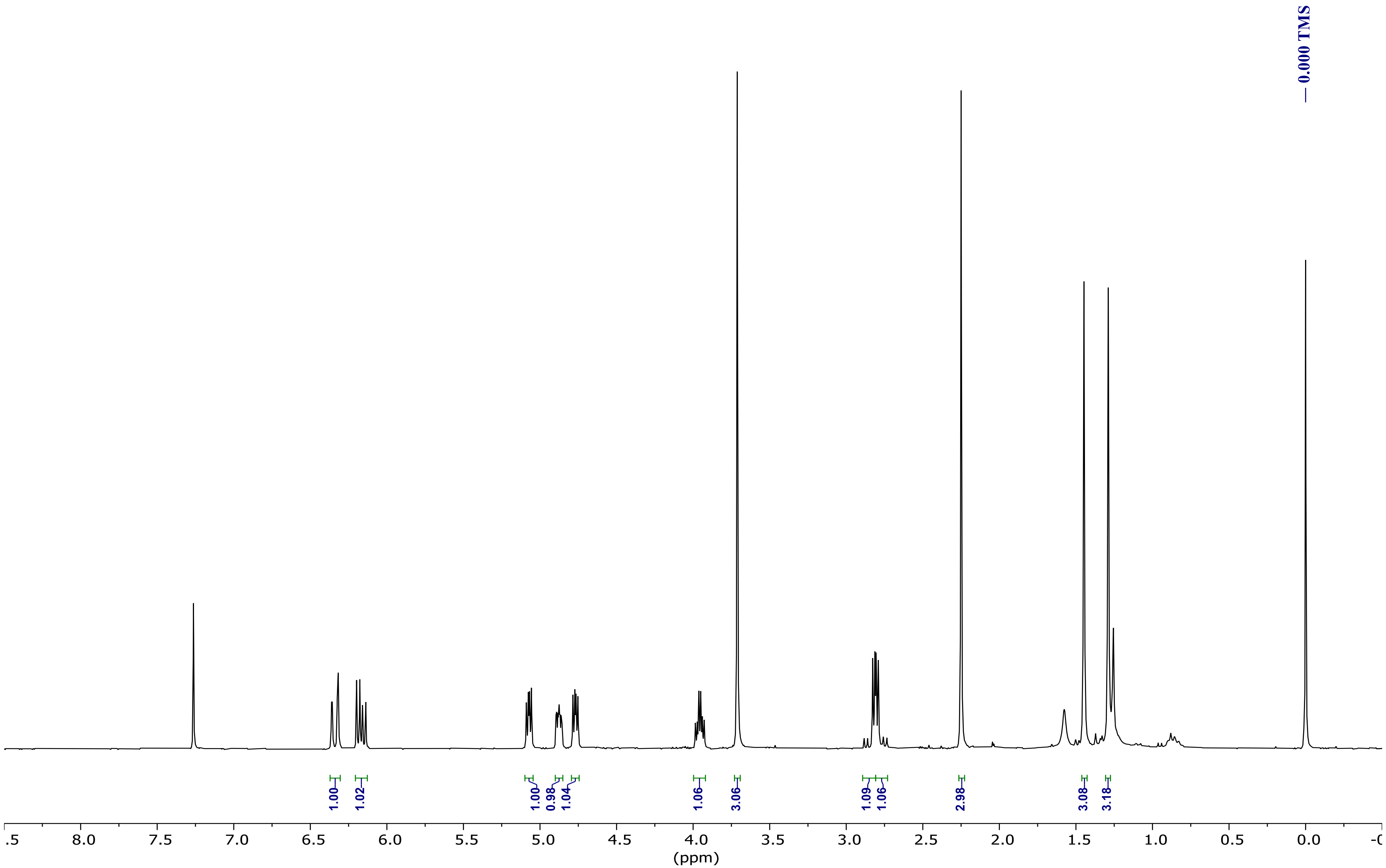

*Methyl 2-((3aR,4S,6R,6aS)-2,2-dimethyl-6-(3-oxobut-1-en-1-yl)tetrahydrofuro[3,4-d][1,3]dioxol-4-yl)acetate (minor-**9**): ^{1}H-NMR (300 MHz, $CDCl_3$)*

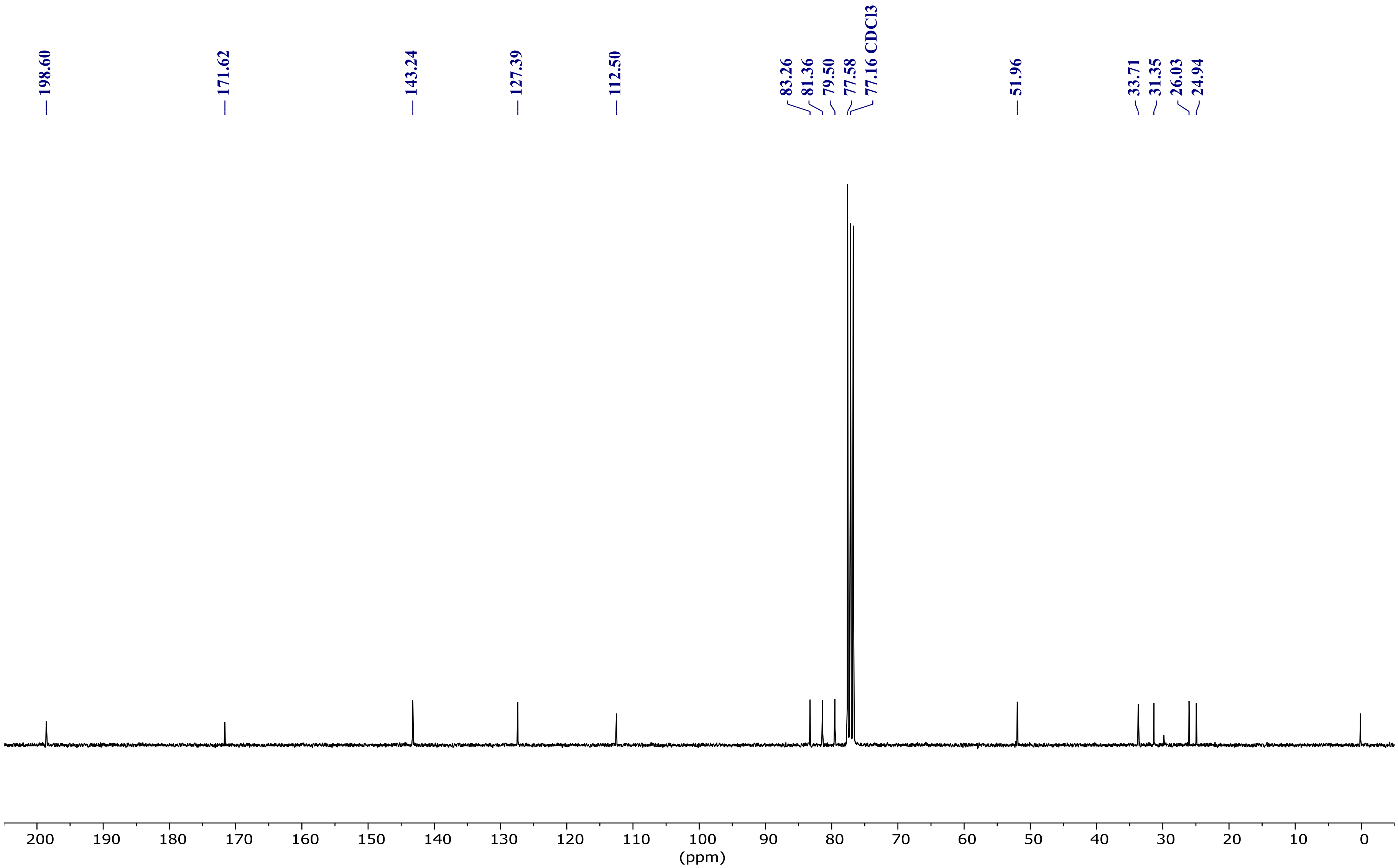

*Methyl 2-((3aR,4S,6R,6aS)-2,2-dimethyl-6-(3-oxobut-1-en-1-yl)tetrahydrofuro[3,4-d][1,3]dioxol-4-yl)acetate (minor-**9**): ^{13}C-NMR (75 MHz, $CDCl_3$)*

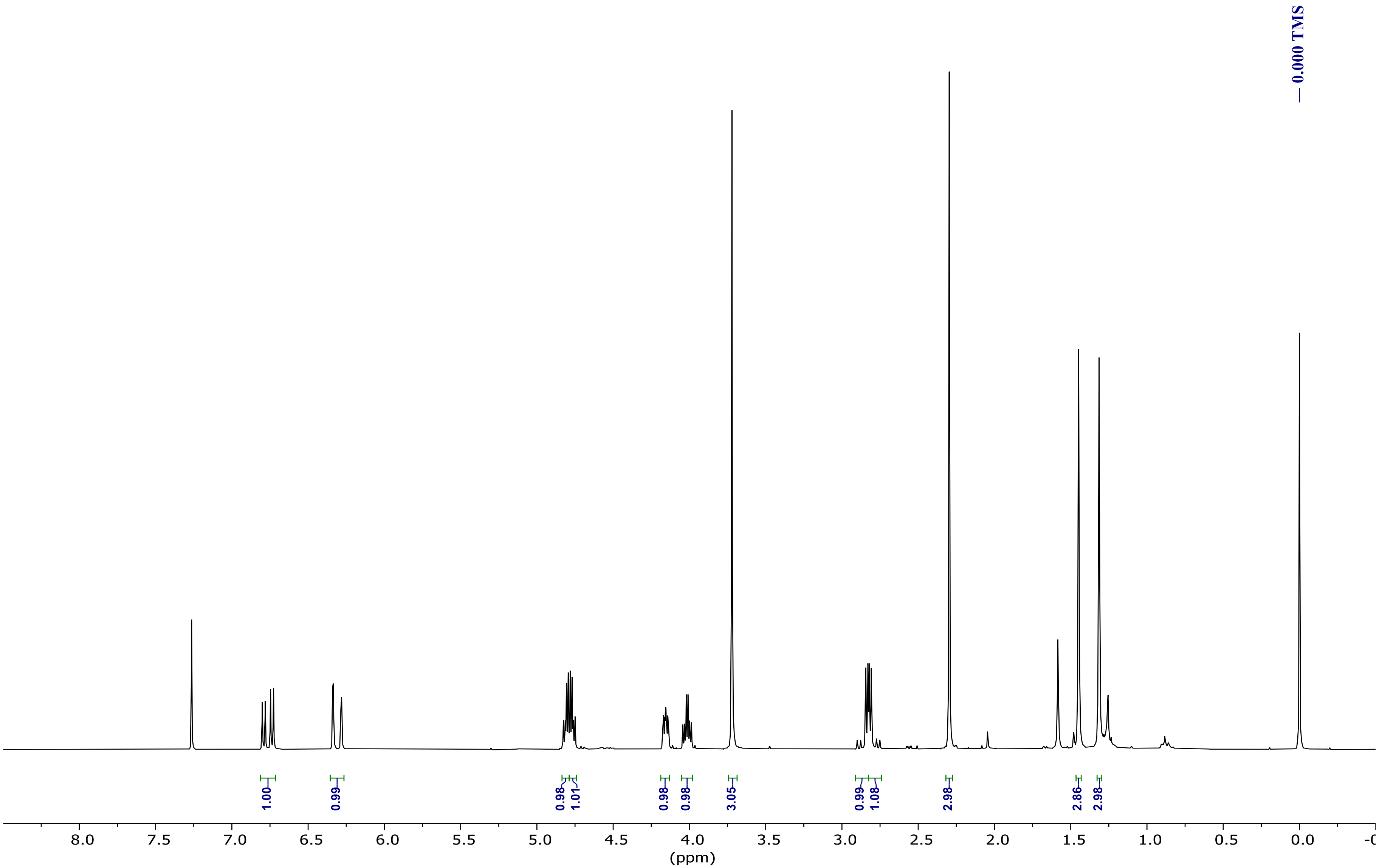

*Methyl 2-((3aR,4S,6R,6aS)-2,2-dimethyl-6-(3-oxobut-1-en-1-yl)tetrahydrofuro[3,4-d][1,3]dioxol-4-yl)acetate (major-**9**): ^{1}H-NMR (300 MHz, $CDCl_3$)*

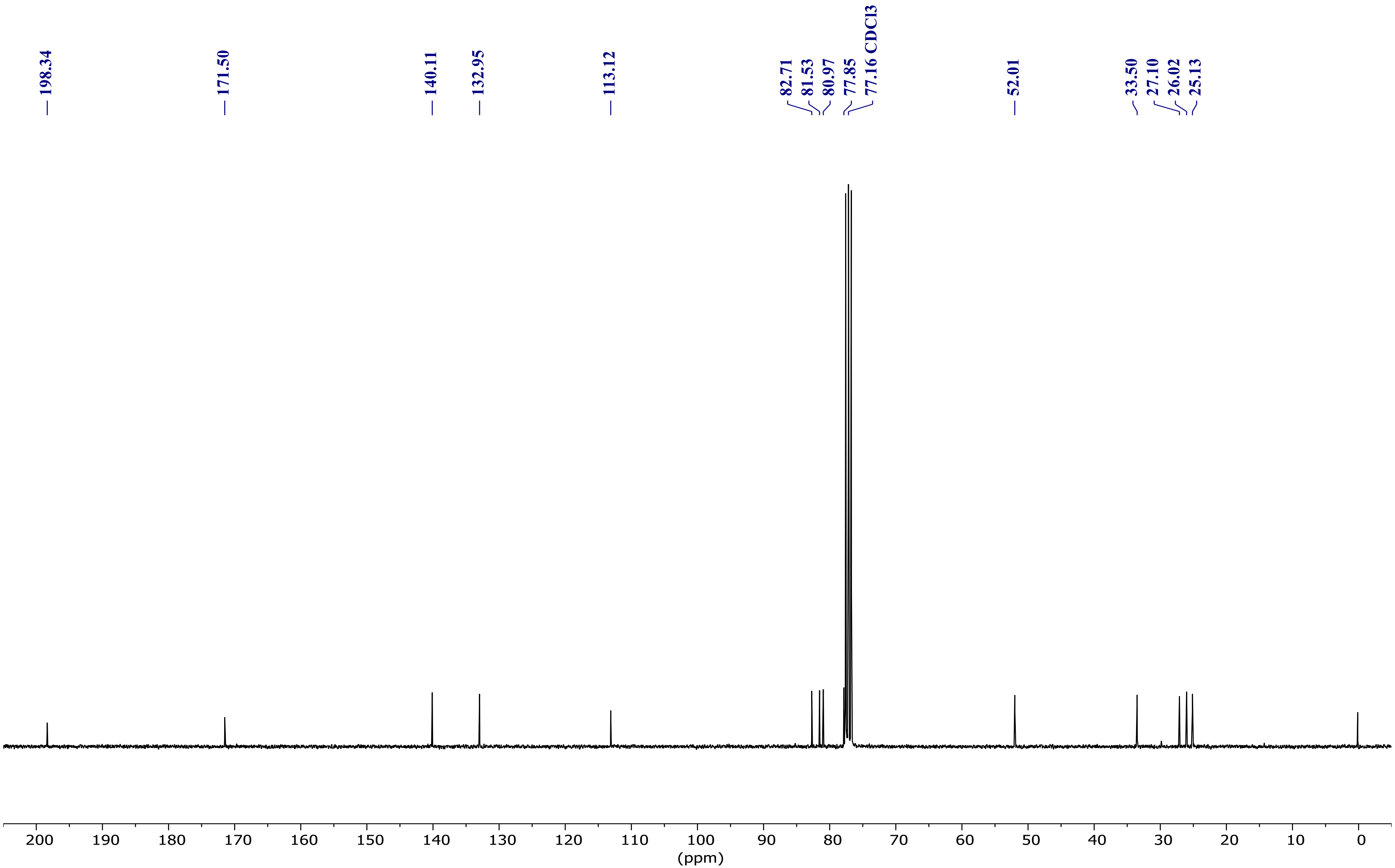

*Methyl 2-((3aR,4S,6R,6aS)-2,2-dimethyl-6-(3-oxobut-1-en-1-yl)tetrahydrofuro[3,4-d][1,3]dioxol-4-yl)acetate (major-**9**): ^{13}C-NMR (75 MHz, $CDCl_3$)*

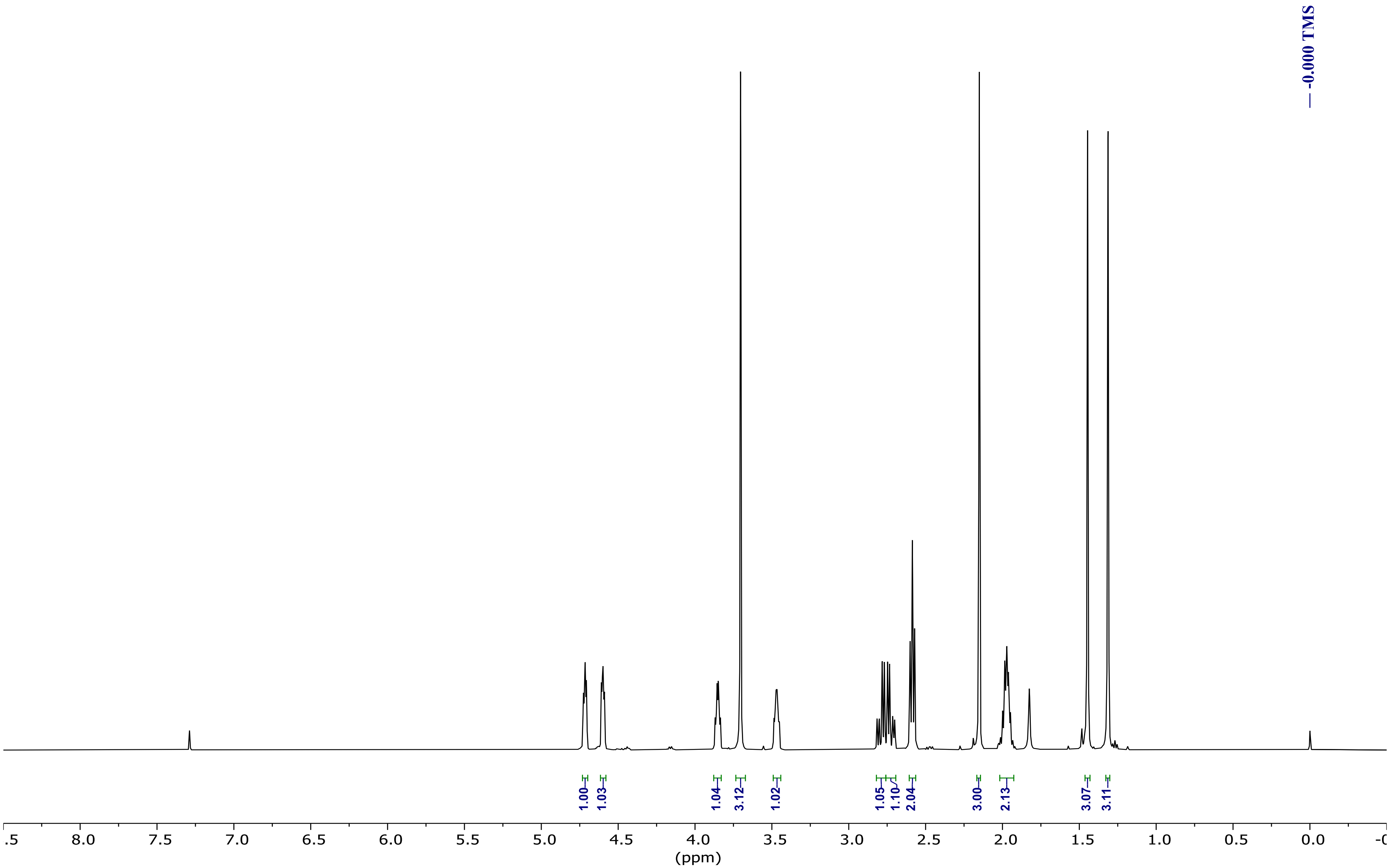

*Methyl 2-((3aR,4S,6R,6aS)-2,2-dimethyl-6-(3-oxobutyl)tetrahydrofuro[3,4-d][1,3]dioxol-4-yl)acetate (**S10**): ^{1}H-NMR (300 MHz, CDCl$_3$)*

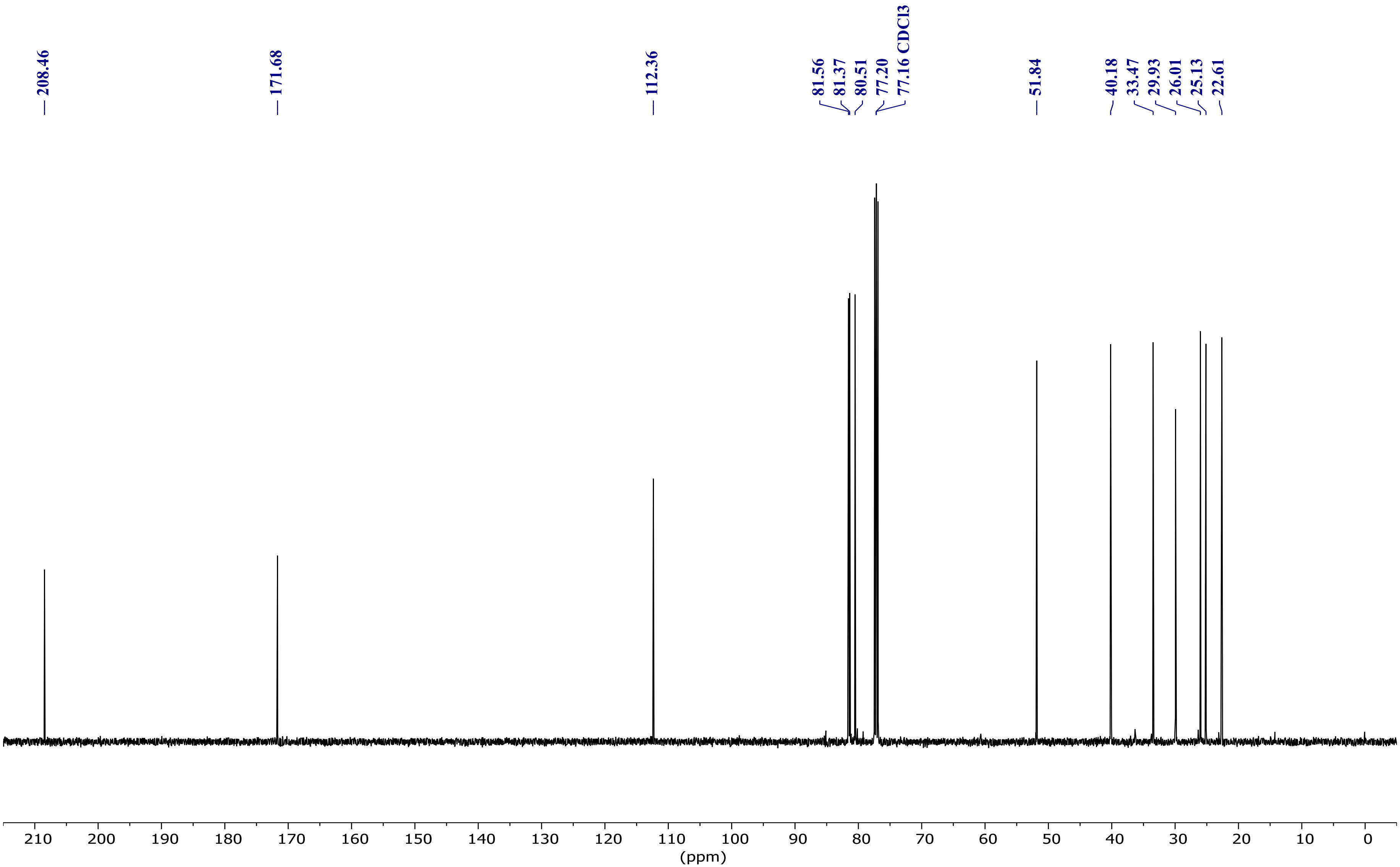

*Methyl 2-((3aR,4S,6R,6aS)-2,2-dimethyl-6-(3-oxobutyl)tetrahydrofuro[3,4-d][1,3]dioxol-4-yl)acetate (**S10**): ^{13}C-NMR (75 MHz, CDCl$_3$)*

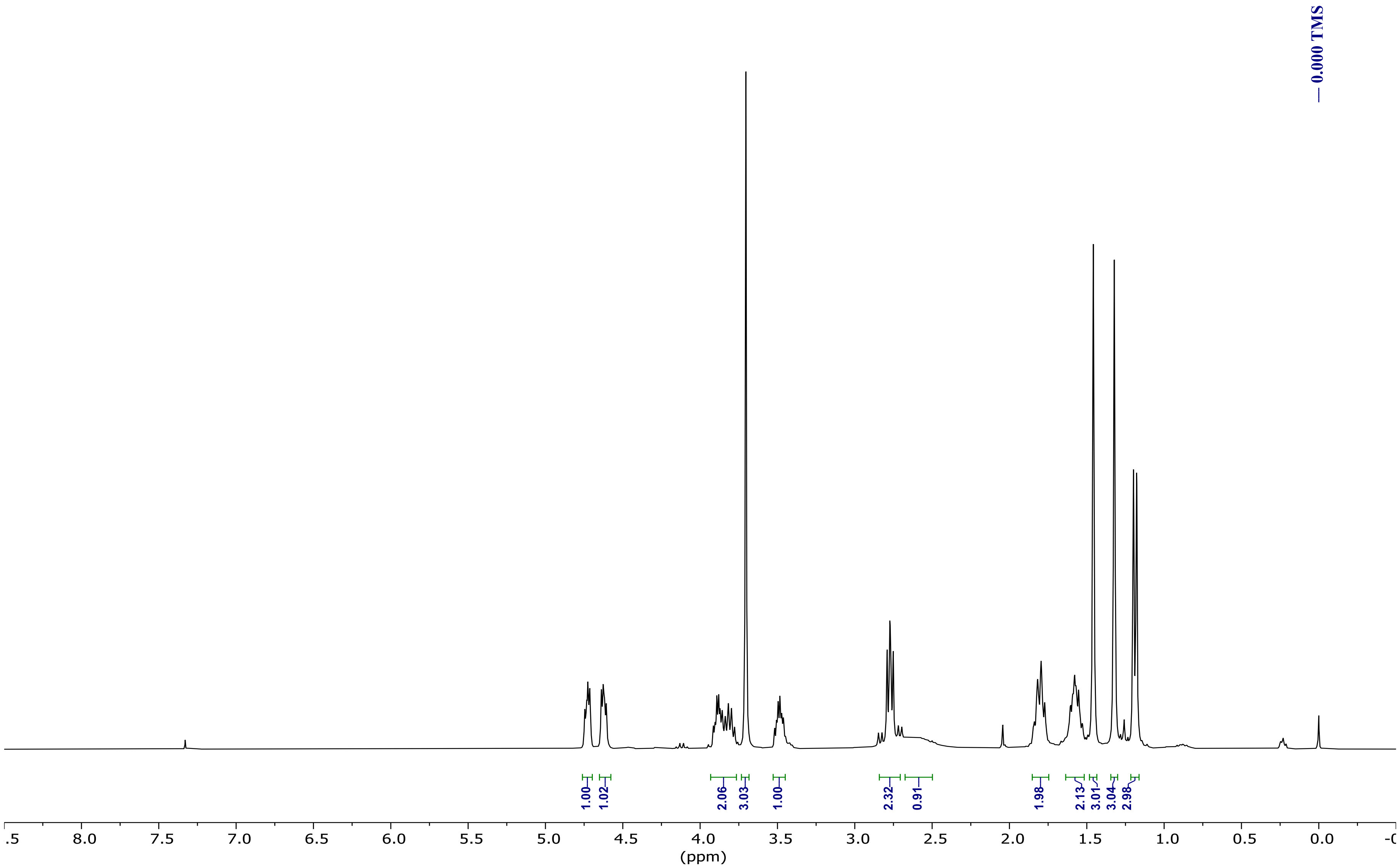

*Methyl 2-((3aR,4S,6R,6aS)-6-((S)-3-hydroxybutyl)-2,2-dimethyltetrahydrofuro[3,4-d][1,3]dioxol-4-yl)acetate (**14**): ^{1}H-NMR (300 MHz, CDCl$_3$)*

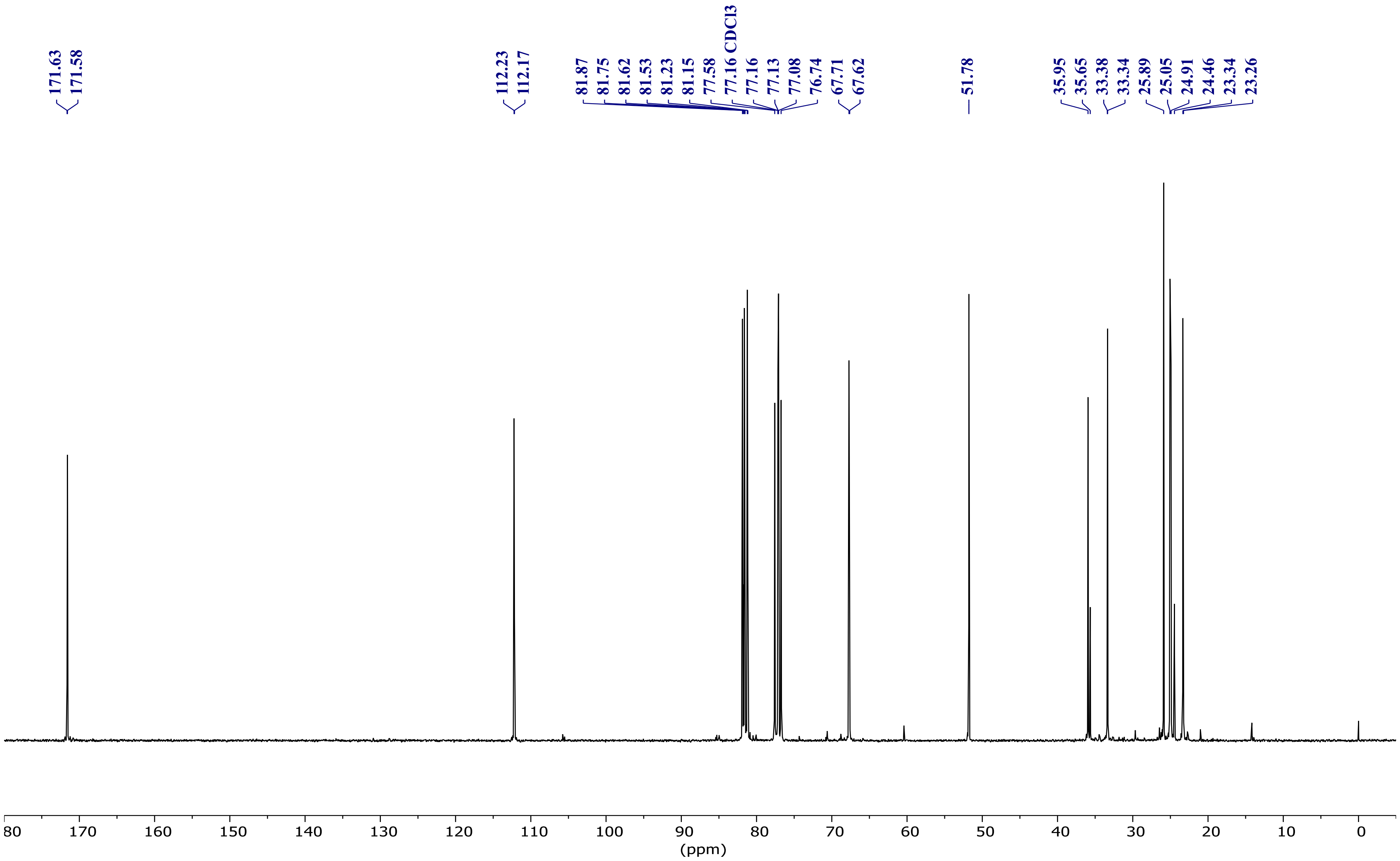

Methyl 2-((3aR,4S,6R,6aS)-6-((S)-3-hydroxybutyl)-2,2-dimethyltetrahydrofuro[3,4-d][1,3]dioxol-4-yl)acetate (**14**): ^{13}C-NMR (75 MHz, CDCl$_3$)

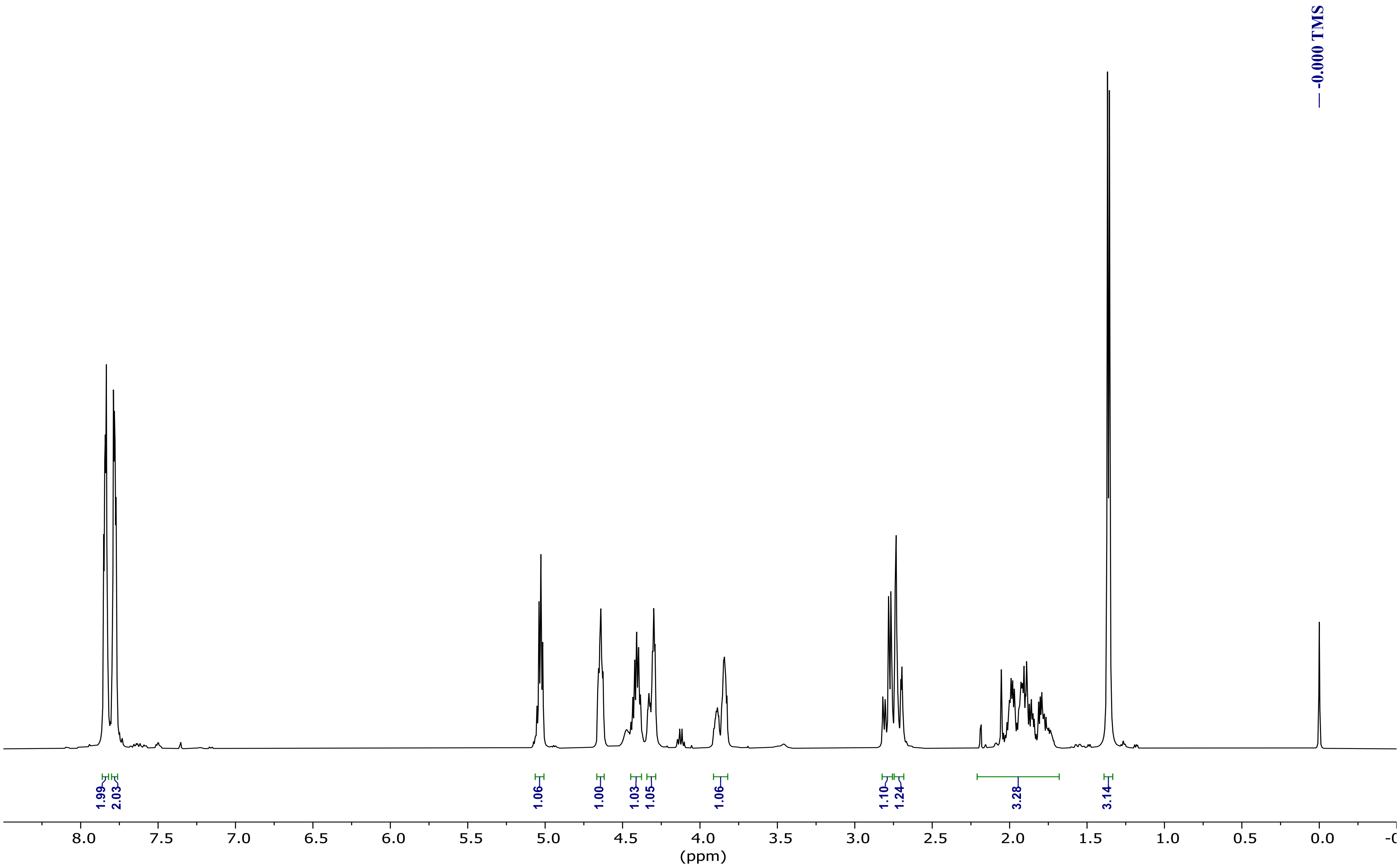

*N-alkoxyphthalimide (**15**): ^{1}H-NMR (500 MHz, $CDCl_3$+one drop CD_3OD)*

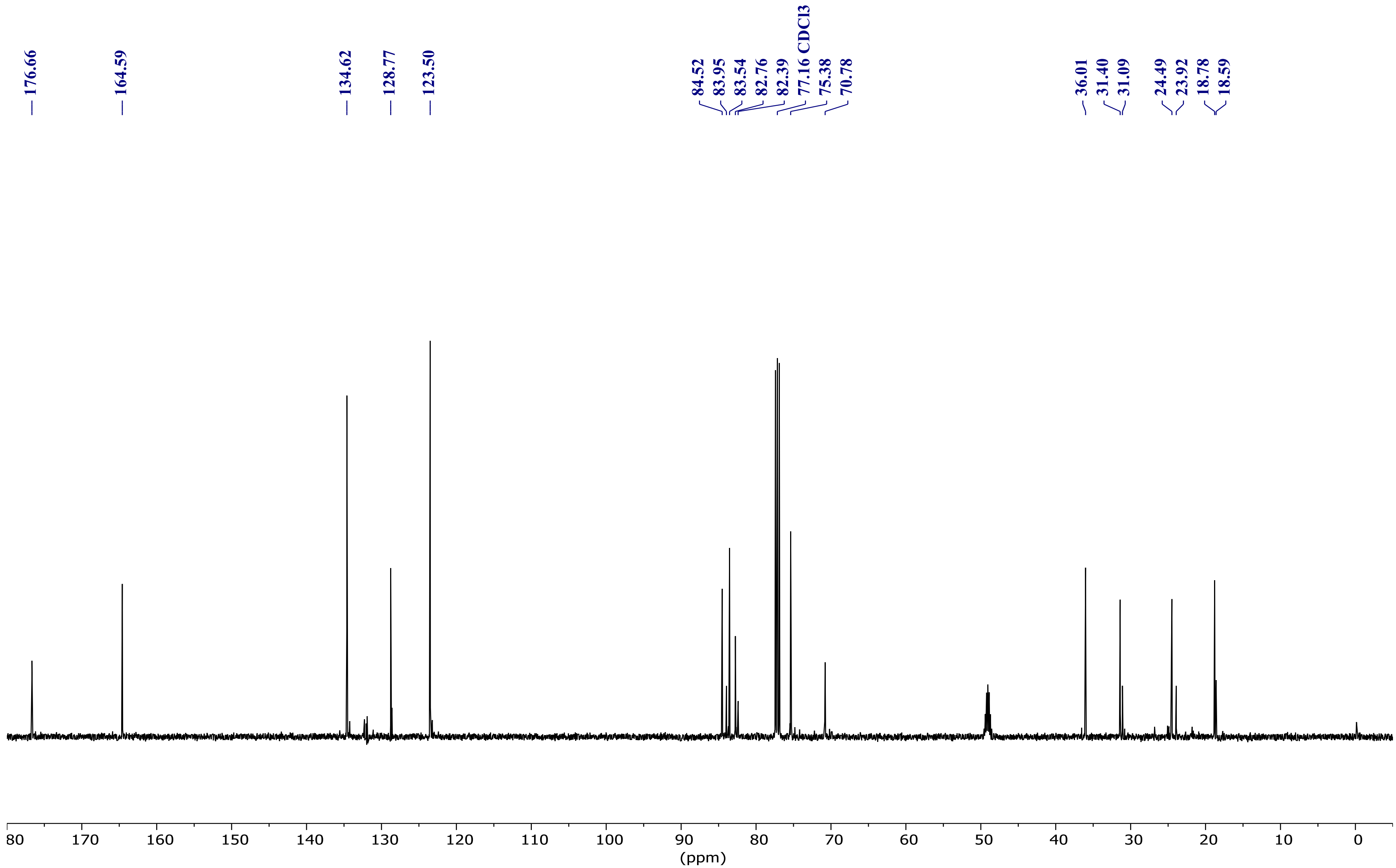

*N-alkoxyphthalimide (**15**): ^{13}C-NMR (125 MHz, $CDCl_3$+one drop CD_3OD)*

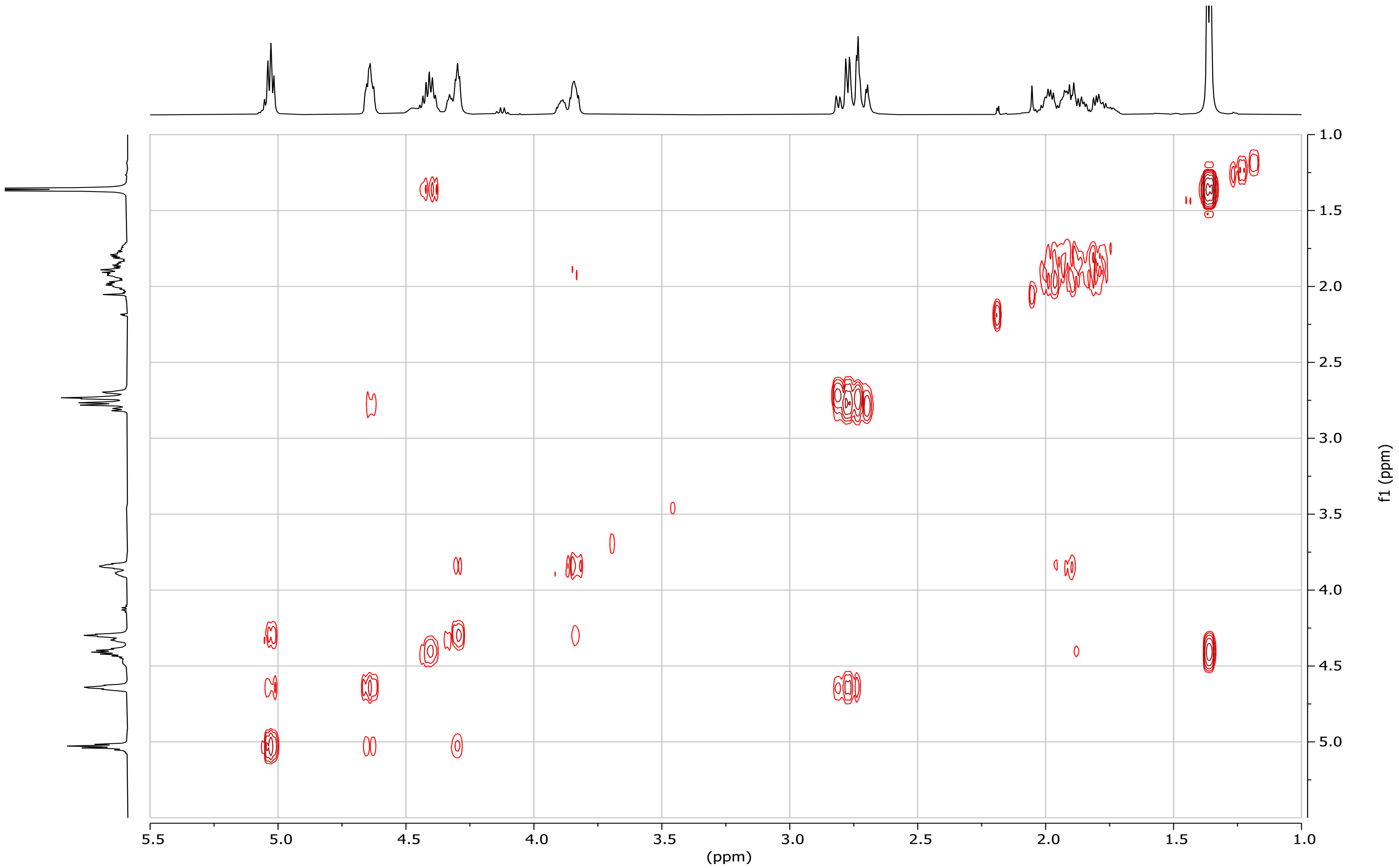

*N-alkoxyphthalimide (**15**): COSY (CDCl$_3$*

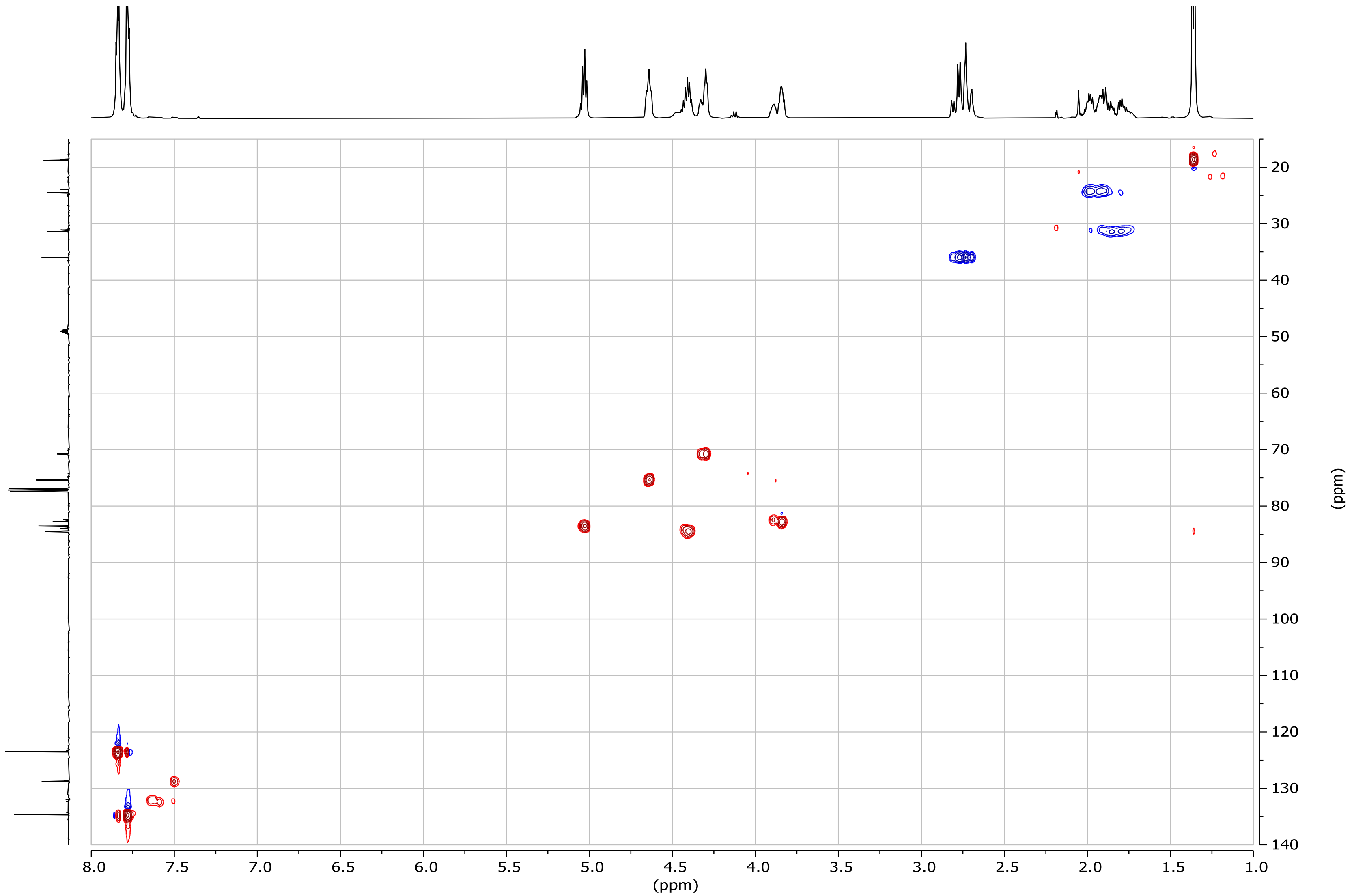

N-alkoxyphthalimide (**15**): HSQC ($CDCl_3$)

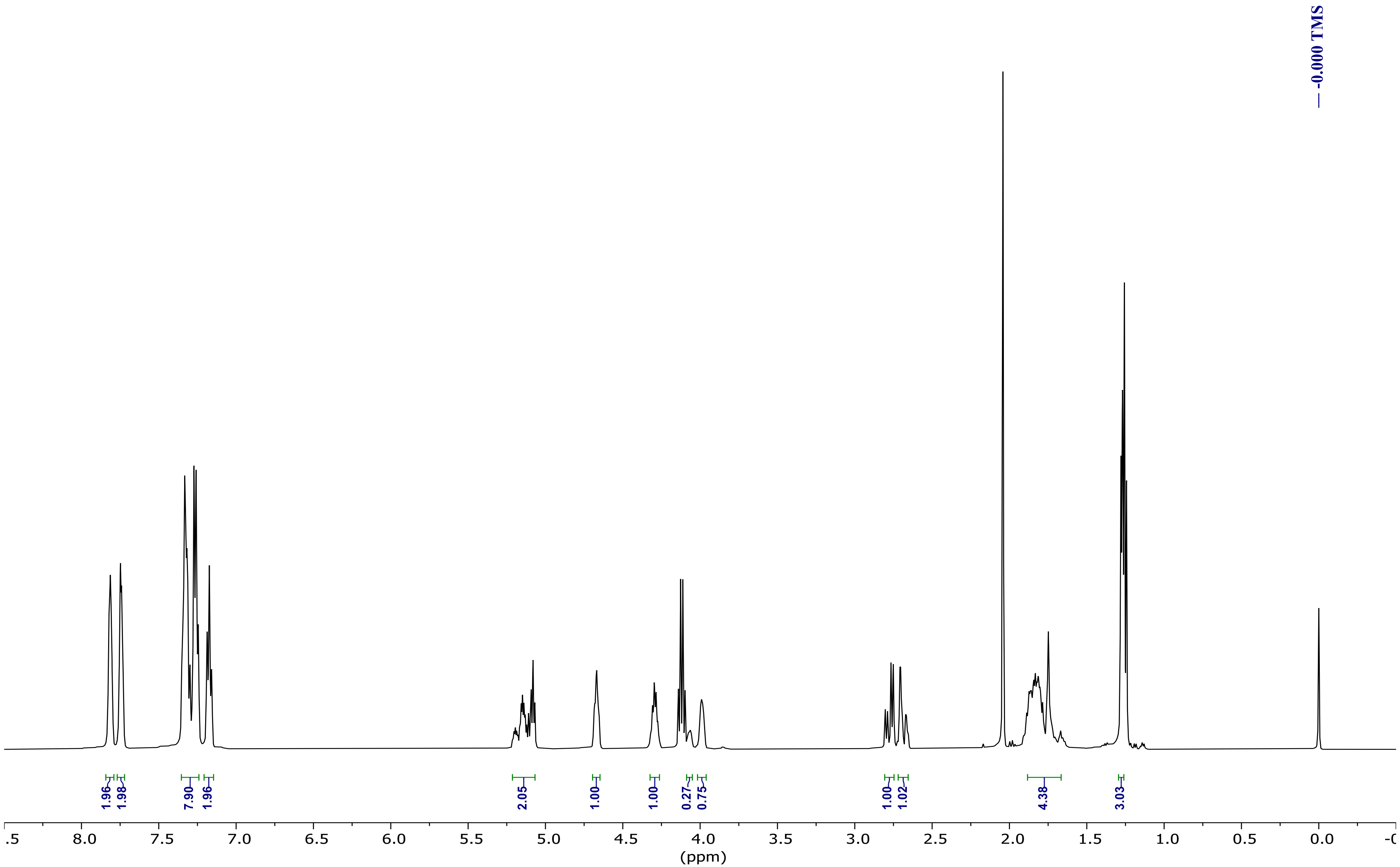

*N-alkoxyphthalimide (**7**): ^{1}H-NMR (500 MHz, $CDCl_3$)*

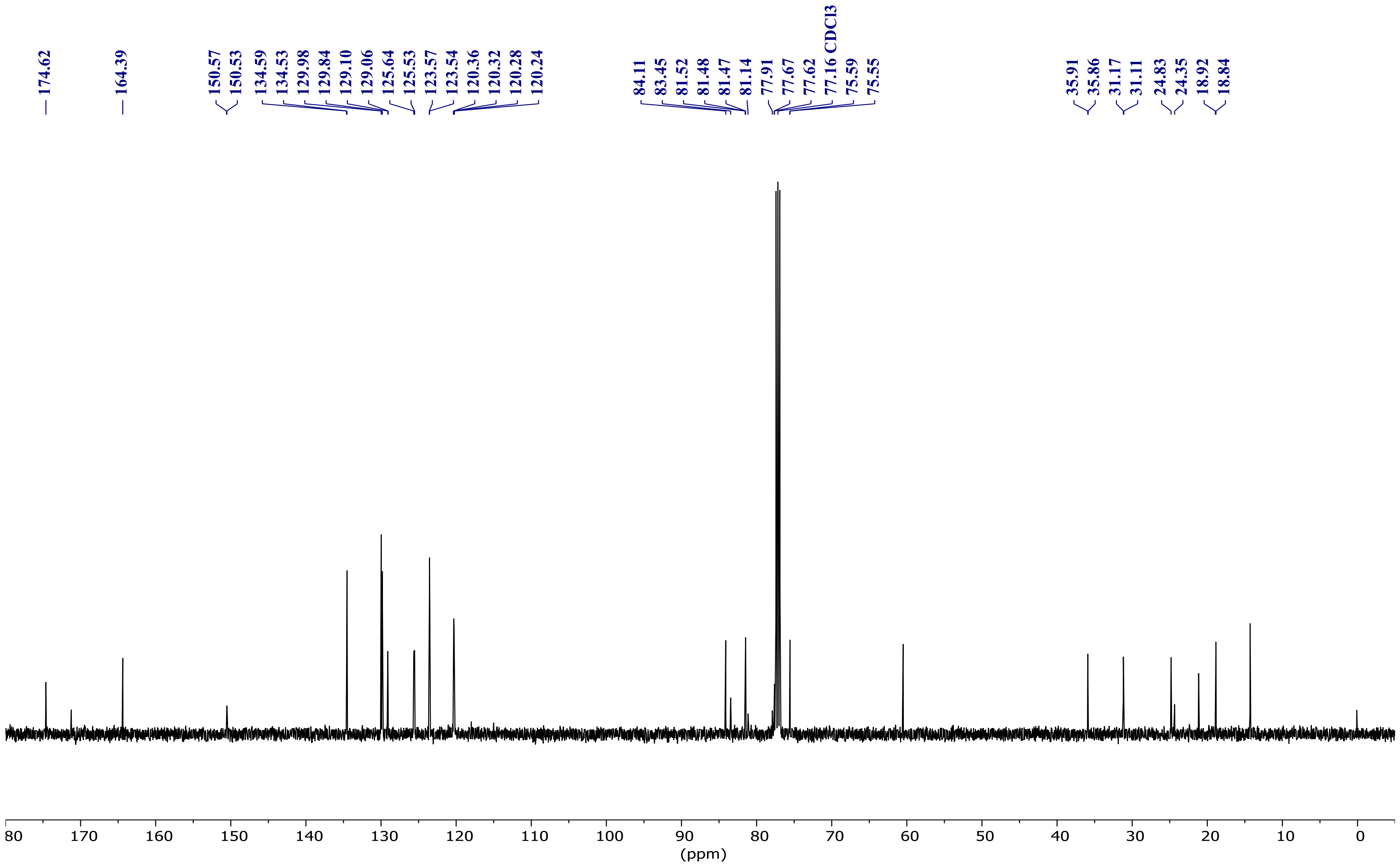

*N-alkoxyphthalimide (**7**): ^{13}C-NMR (125 MHz, $CDCl_3$)*

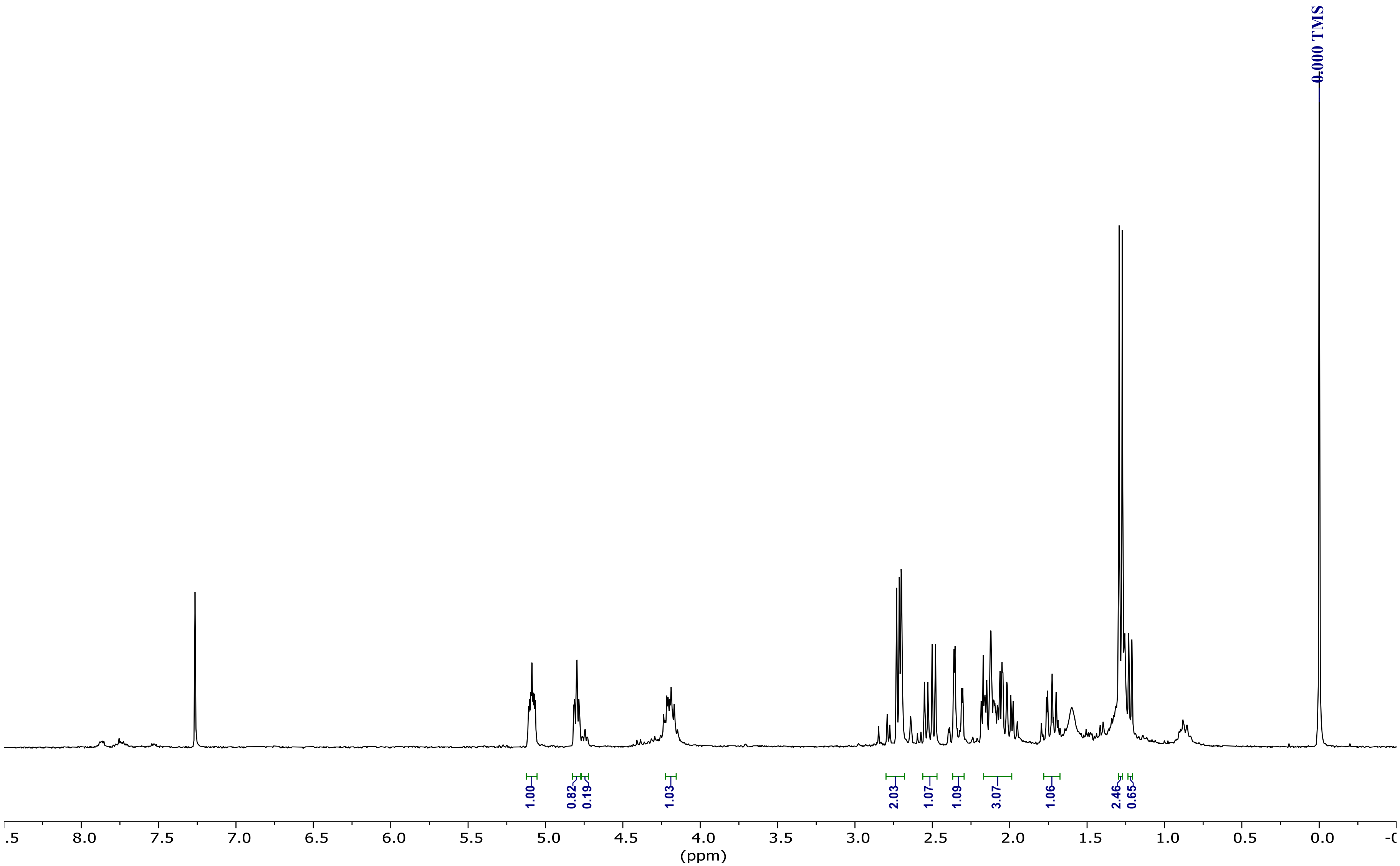

(-)-Cep F *+ C9-epimer (4:1):* ^{1}*H-NMR (300 MHz, CDCl*$_3$*)*

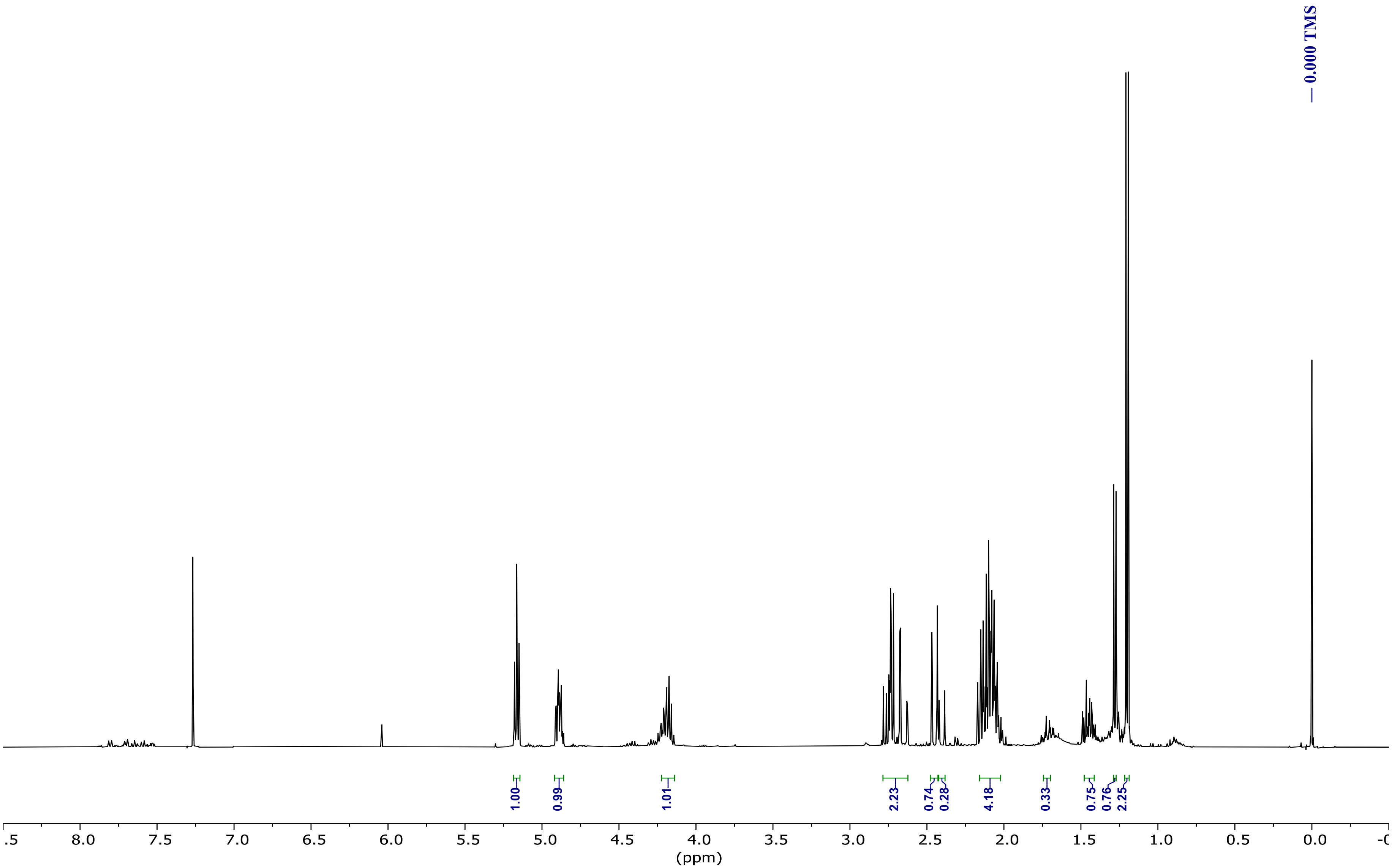

(+)-Cep E *+ C9-epimer (3:1):* ^{1}*H-NMR (400 MHz,* $CDCl_3$*)*

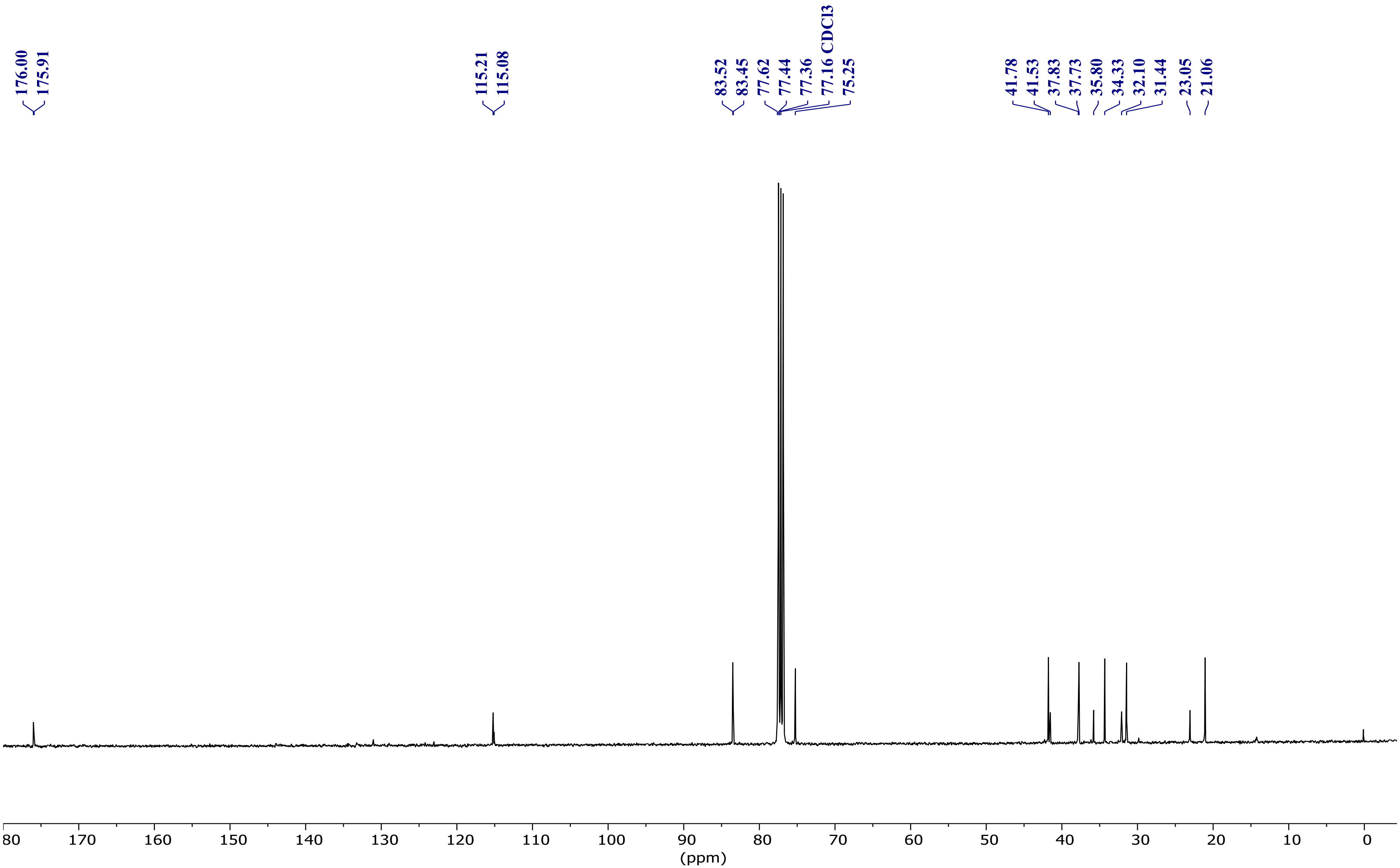

(+)-Cep E *+ C9-epimer (3:1):* ^{13}*C-NMR (100 MHz, CDCl*$_3$*)*

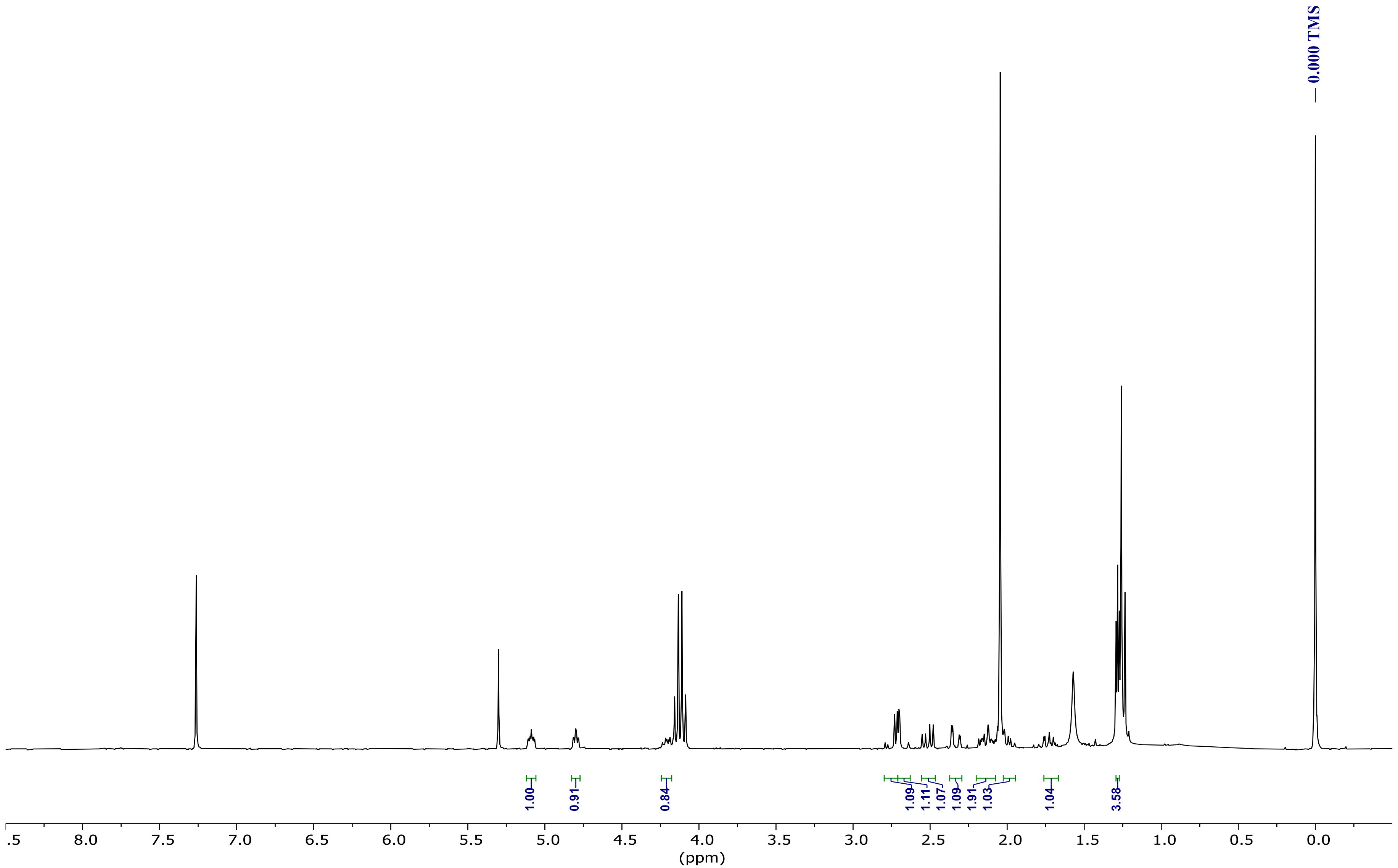

(+)-Cep F: *^{1}H-NMR (300 MHz, $CDCl_3$)*

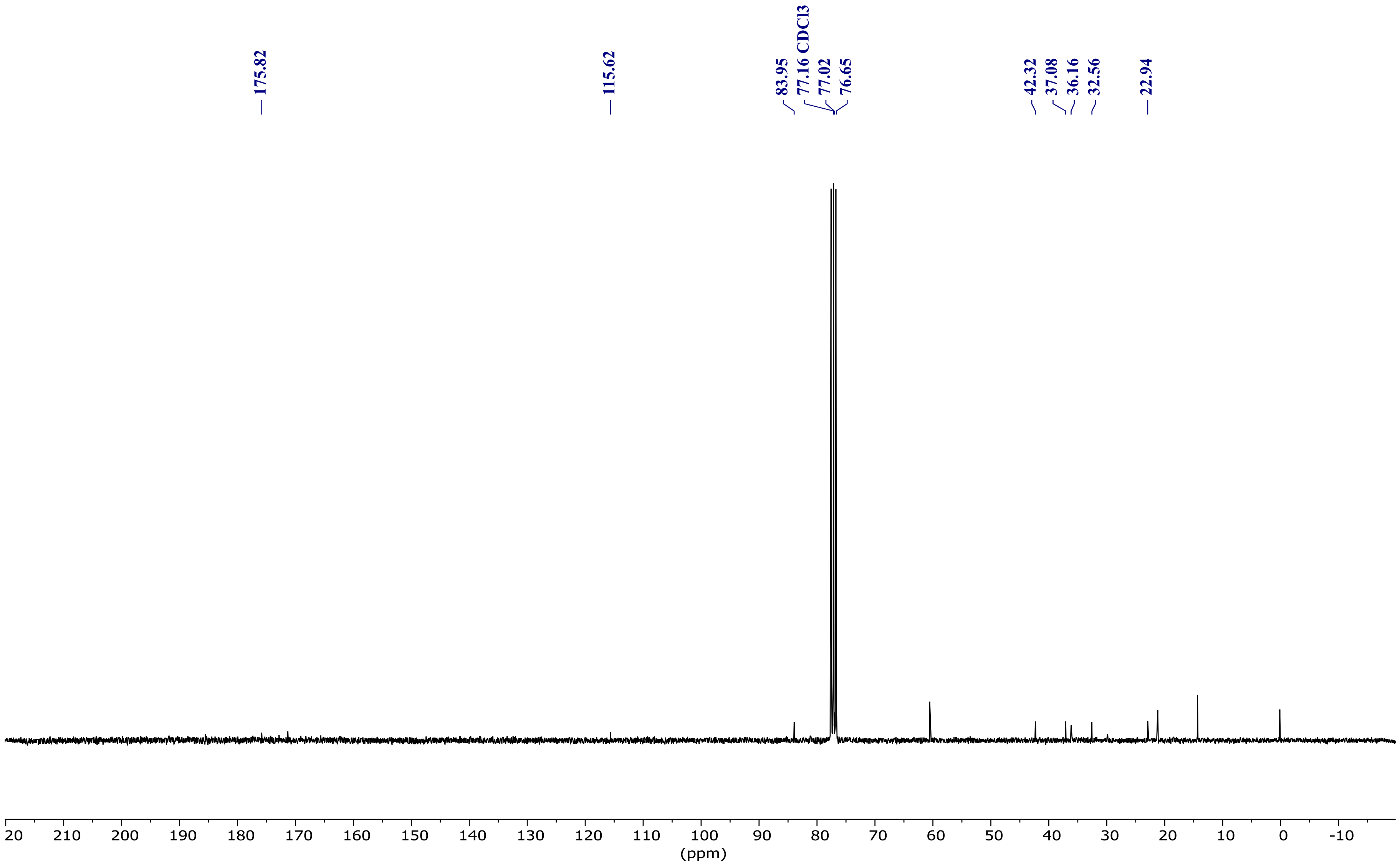

(+)-Cep F: ^{13}*C-NMR (75 MHz, CDCl$_3$)*

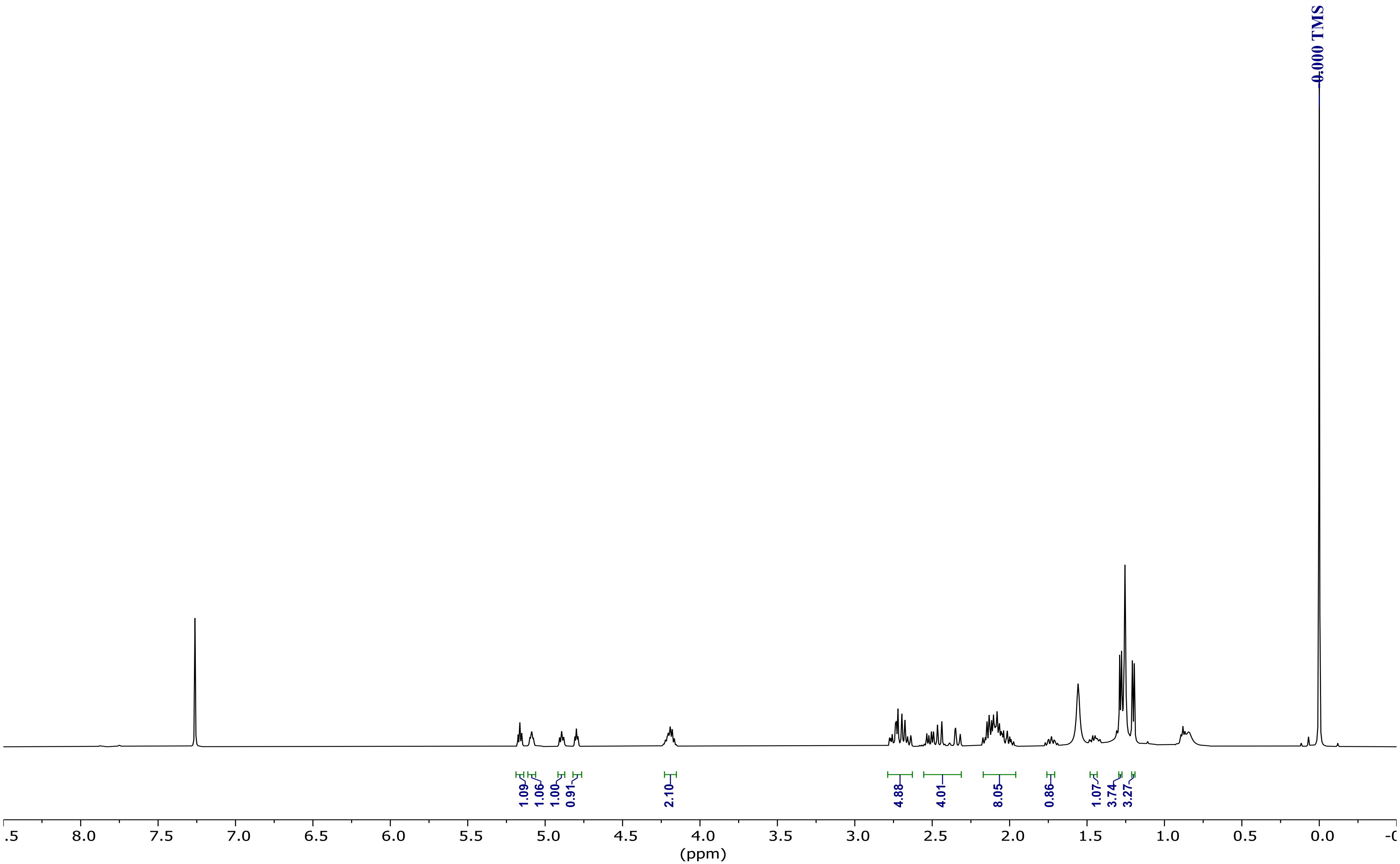

(+)-Cep F *+* ***(-)-Cep E*** *(1:1):* [1]*H-NMR (500 MHz,* $CDCl_3$*)*

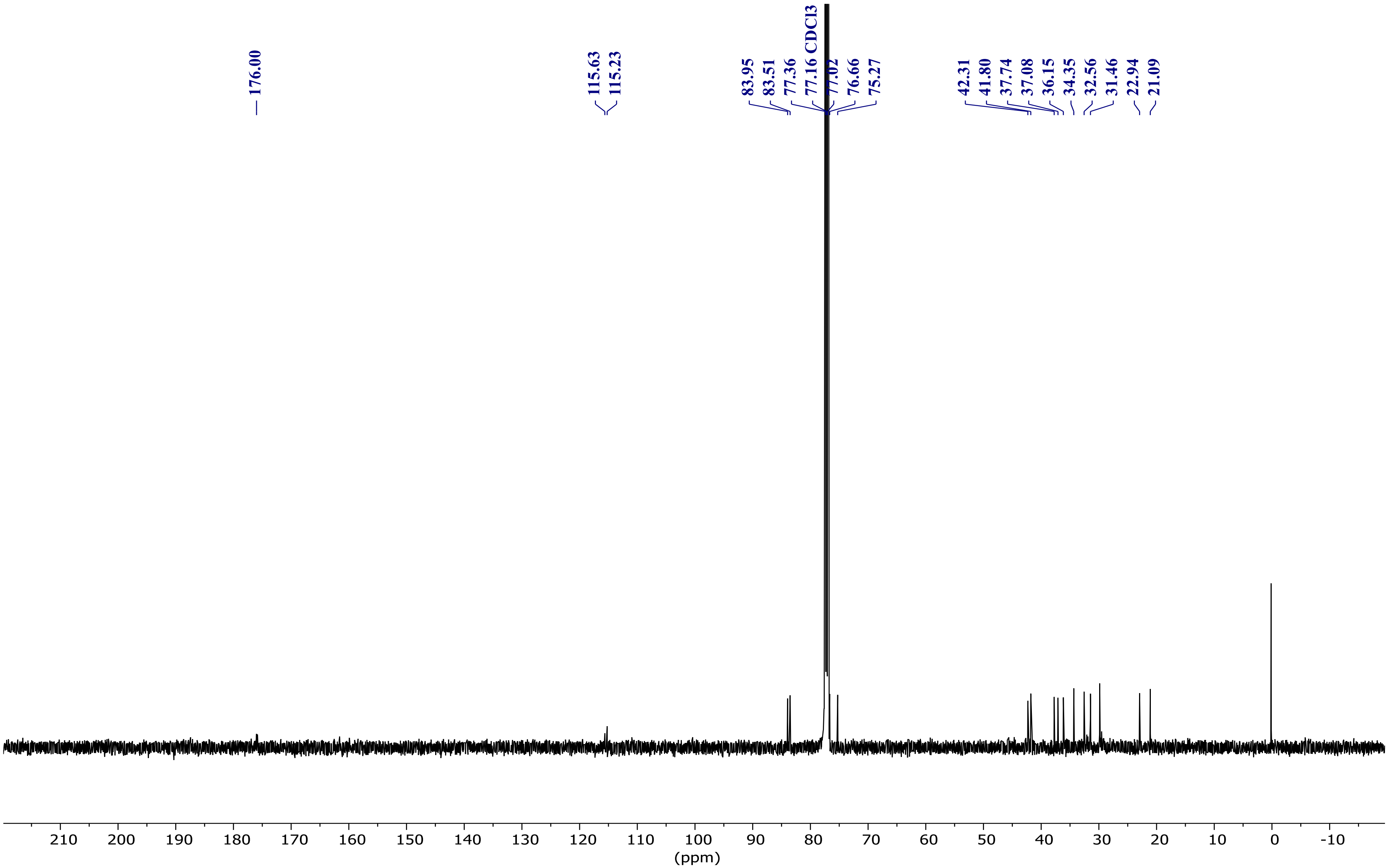

***(+)-Cep F** + **(-)-Cep E** (1:1): ^{1}H-NMR (125 MHz, CDCl$_3$)*

HRMS Spectra

Spectrum

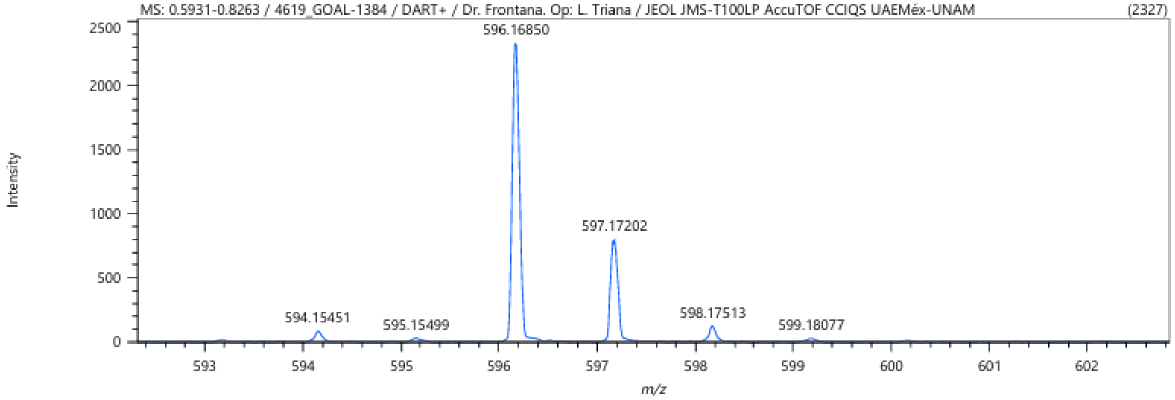

Elemental Composition

Parameters		Elements Set 4:					
Tolerance:	±3.00 ppm	Symbol	C	H	O	N	P
Electron:	Odd/Even	Min	0	0	0	0	0
Charge:	+1	Max	50	50	10	1	1
DBE:	-15.0 - 20.0						

Results

Mass	Intensity	Intensity [%]	Formula	Calculated Mass	Mass Difference [mDa]	Mass Difference [ppm]	DBE
596.16850	2326.96	2.69	C30 H31 N O10 P	596.16801	0.49	0.82	16.5

*N-alkoxyphthalimide (**2a**)*

Spectrum

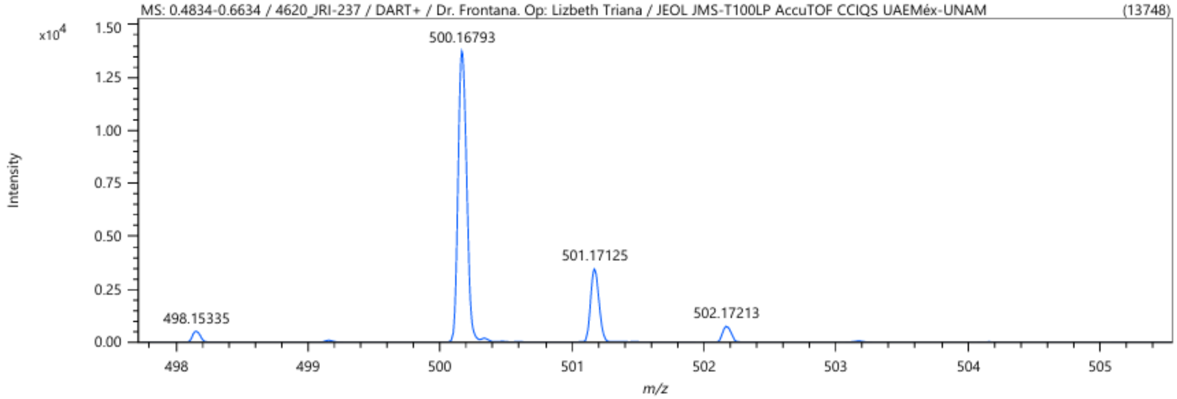

Elemental Composition

Parameters		Elements Set 4:					
Tolerance:	±3.00 ppm	Symbol	C	H	O	N	P
Electron:	Odd/Even	Min	0	0	0	0	0
Charge:	+1	Max	50	50	10	1	1
DBE:	-15.0 - 12.0						

Results

Mass	Intensity	Intensity [%]	Formula	Calculated Mass	Mass Difference [mDa]	Mass Difference [ppm]	DBE
500.16793	13747.63	9.72	C22 H31 N O10 P	500.16801	-0.08	-0.17	8.5

*N-alkoxyphthalimide (**2b**)*

Spectrum

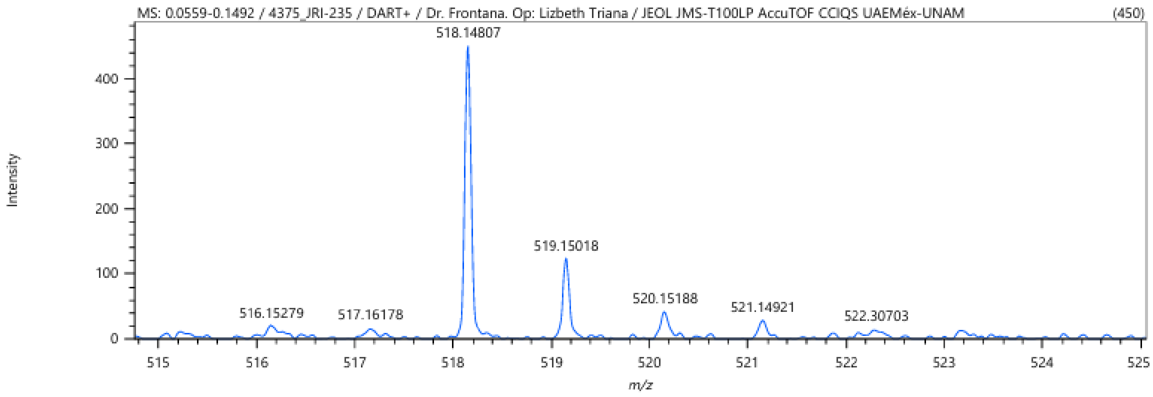

Elemental Composition

Parameters		Elements Set 6:					
Tolerance:	±5.00 ppm	Symbol	C	H	N	O	S
Electron:	Odd/Even	Min	0	0	0	0	0
Charge:	+1	Max	50	50	2	10	2
DBE:	-10.0 - 15.0						

Results

Mass	Intensity	Intensity [%]	Formula	Calculated Mass	Mass Difference [mDa]	Mass Difference [ppm]	DBE
518.14807	449.88	0.84	C25 H28 N O9 S	518.14793	0.14	0.28	12.5

*N-alkoxyphthalimide (**2c**)*

INSTITUTO DE QUIMICA, UNAM
LABORATORIO DE ESPECTROMETRIA DE MASAS

Data:96_STA-3804 Goal-1278
Sample Name:DR. Frontana Bernardo / Operador: Carmen Garcia
Description:
Ionization Mode:ESI+
History:Determine m/z[Peak Detect[Centroid,30,Area];Correct Base[];Smooth[5]];Correct Base[5.0%];Average(MS[...

Acquired:6/13/2023 1:29:03 PM
Operator:AccuTOF
Mass Calibration data:Cal_PEG_600
Created:6/14/2023 12:41:33 PM
Created by:AccuTOF

Charge number:1
Tolerance:3.00(mmu)
Unsaturation Number:-1.0 .. 60.0 (Fraction:Both)
Element:^{12}C:15 .. 20, ^{1}H:0 .. 27, ^{14}N:0 .. 2, ^{16}O:0 .. 8

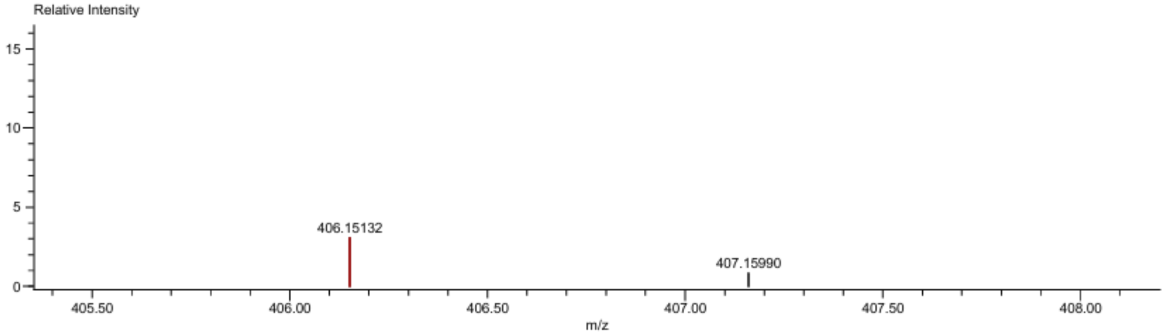

Mass	Intensity	Calc. Mass	Mass Difference (mmu)	Mass Difference (ppm)	Possible Formula	Unsaturation Number
406.15132	37852.80	406.15019	1.13	2.78	$^{12}C_{20}{}^{1}H_{24}{}^{14}N_{1}{}^{16}O_{8}$	9.5

*N-alkoxyphthalimide (**2d**)*

Spectrum

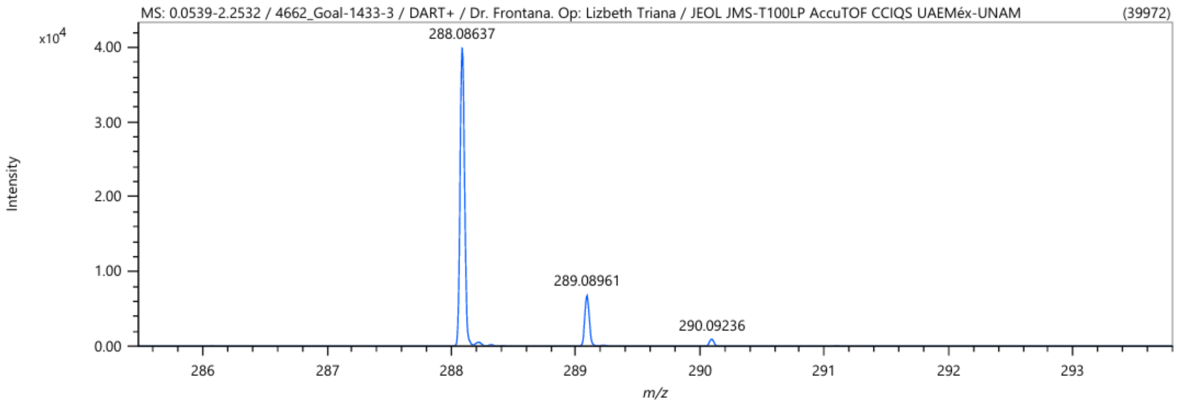

Elemental Composition

Parameters		Elements Set 3:				
Tolerance:	±3.00 ppm	Symbol	C	H	O	N
Electron:	Odd/Even	Min	0	0	0	0
Charge:	+1	Max	50	50	5	1
DBE:	-15.0 - 25.0					

Results

Mass	Intensity	Intensity [%]	Formula	Calculated Mass	Mass Difference [mDa]	Mass Difference [ppm]	DBE
288.08637	39972.12	100.00	C15 H14 N O5	288.08665	-0.28	-0.97	9.5

*N-alkoxyphthalimide (**2e**)*

Spectrum

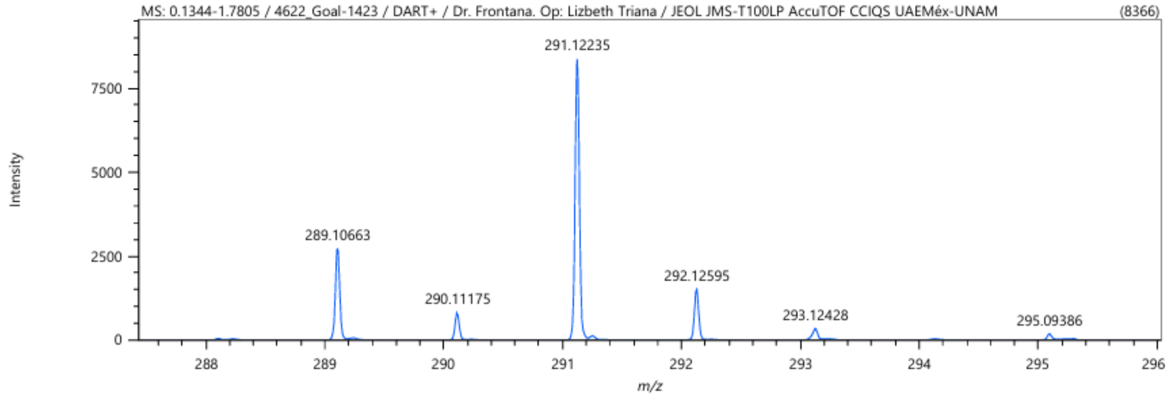

Elemental Composition

Parameters		Elements Set 4:				
Tolerance:	±3.00 ppm	Symbol	C	H	O	N
Electron:	Odd/Even	Min	0	0	0	0
Charge:	+1	Max	50	50	5	0
DBE:	-15.0 - 25.0					

Results

Mass	Intensity	Intensity [%]	Formula	Calculated Mass	Mass Difference [mDa]	Mass Difference [ppm]	DBE
291.12235	8365.90	32.47	C16 H19 O5	291.12270	-0.35	-1.19	7.5

*α,β-unsaturated ketone (**S5**)*

Spectrum

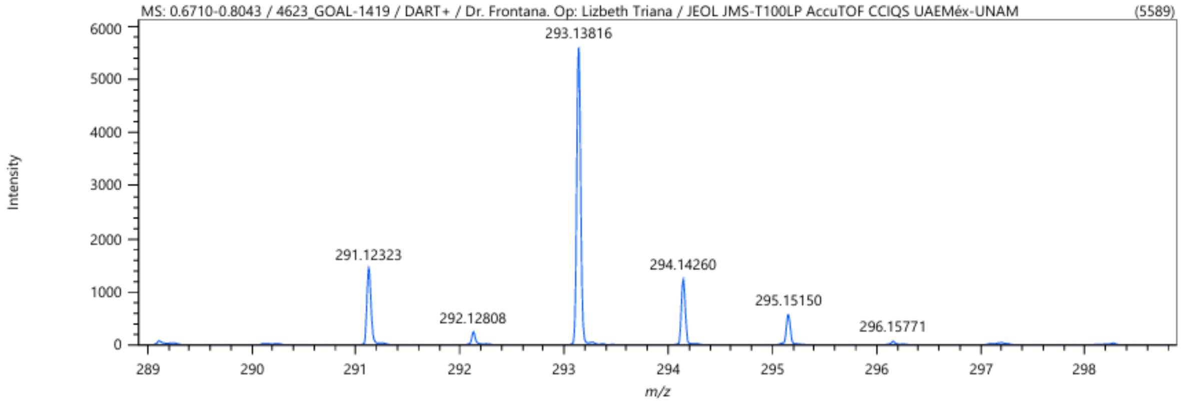

Elemental Composition

Parameters		Elements Set 4:				
Tolerance:	±3.00 ppm	Symbol	C	H	O	N
Electron:	Odd/Even	Min	0	0	0	0
Charge:	+1	Max	50	50	5	0
DBE:	-15.0 - 25.0					

Results

Mass	Intensity	Intensity [%]	Formula	Calculated Mass	Mass Difference [mDa]	Mass Difference [ppm]	DBE
293.13816	5589.06	4.24	C16 H21 O5	293.13835	-0.19	-0.67	6.5

Spectrum

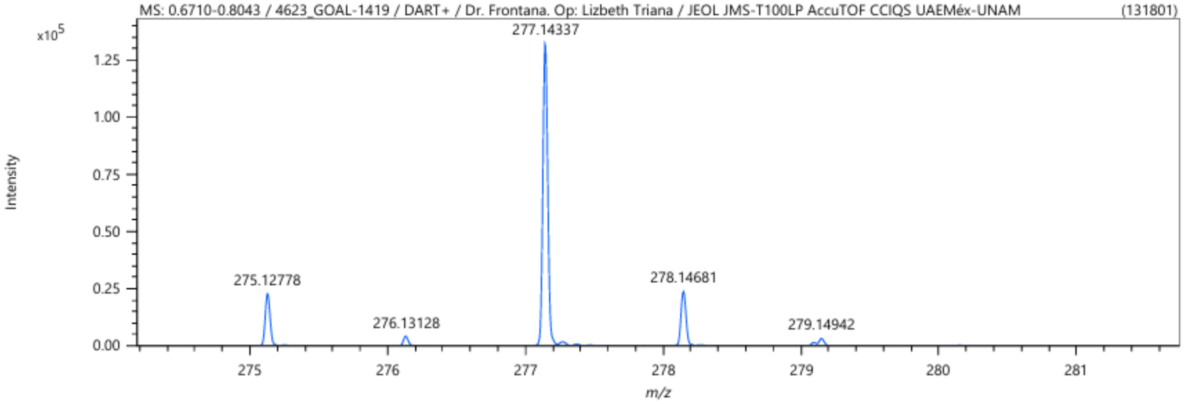

Elemental Composition

Parameters		Elements Set 4:				
Tolerance:	±3.00 ppm	Symbol	C	H	O	N
Electron:	Odd/Even	Min	0	0	0	0
Charge:	+1	Max	50	50	5	0
DBE:	-15.0 - 25.0					

Results

Mass	Intensity	Intensity [%]	Formula	Calculated Mass	Mass Difference [mDa]	Mass Difference [ppm]	DBE
277.14337	131800.72	100.00	C16 H21 O4	277.14344	-0.06	-0.23	6.5

*(3aR,5R,6S,6aR)-5-(3-hydroxy-3-phenylpropyl)-2,2-dimethyltetrahydrofuro[2,3-d][1,3]dioxol-6-ol (**S6**)*

Spectrum

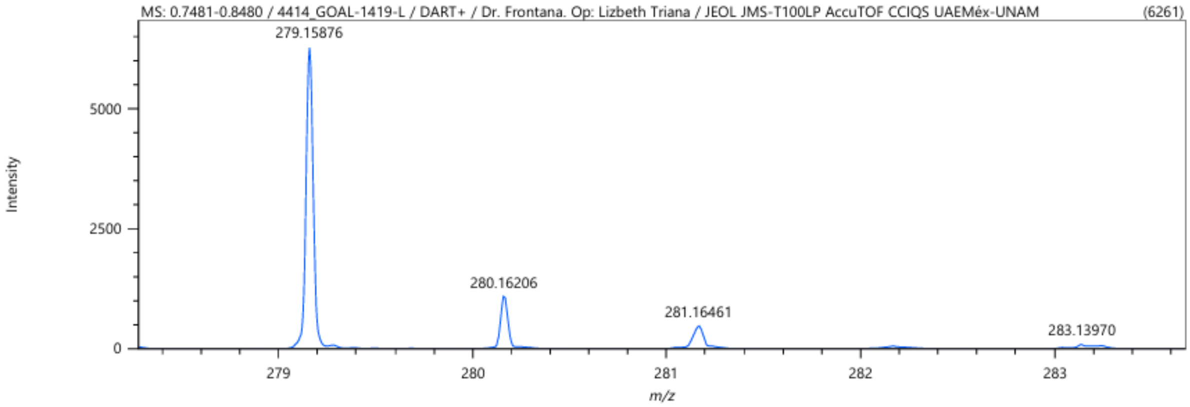

Elemental Composition

Parameters		Elements Set 2:				
Tolerance:	±10.00 ppm	Symbol	C	H	O	N
Electron:	Odd/Even	Min	0	0	0	0
Charge:	+1	Max	100	100	4	0
DBE:	-15.0 - 11.0					

Results

Mass	Intensity	Intensity [%]	Formula	Calculated Mass	Mass Difference [mDa]	Mass Difference [ppm]	DBE
279.15876	6260.92	7.49	C16 H23 O4	279.15909	-0.33	-1.18	5.5

*(3aR,5R,6S,6aR)-2,2-dimethyl-5-(3-phenylpropyl)tetrahydrofuro[2,3-d][1,3]dioxol-6-ol (**S7**)*

Spectrum

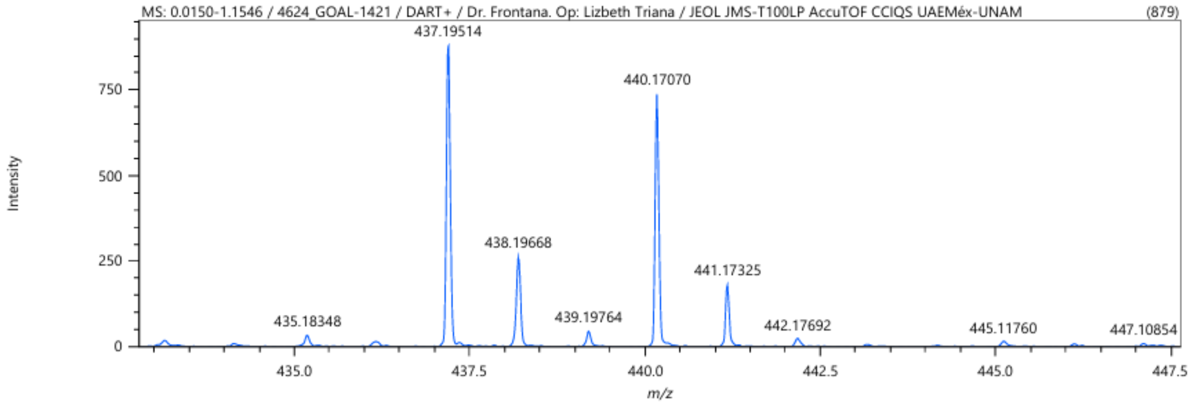

Elemental Composition

Parameters		Elements Set 4:				
Tolerance:	±3.00 ppm	Symbol	C	H	O	N
Electron:	Odd/Even	Min	0	0	0	0
Charge:	+1	Max	50	50	7	1
DBE:	-15.0 - 25.0					

Results

Mass	Intensity	Intensity [%]	Formula	Calculated Mass	Mass Difference [mDa]	Mass Difference [ppm]	DBE
440.17070	738.10	2.26	C24 H26 N O7	440.17038	0.32	0.72	12.5

*N-alkoxyphthalimide (**S8**)*

Spectrum

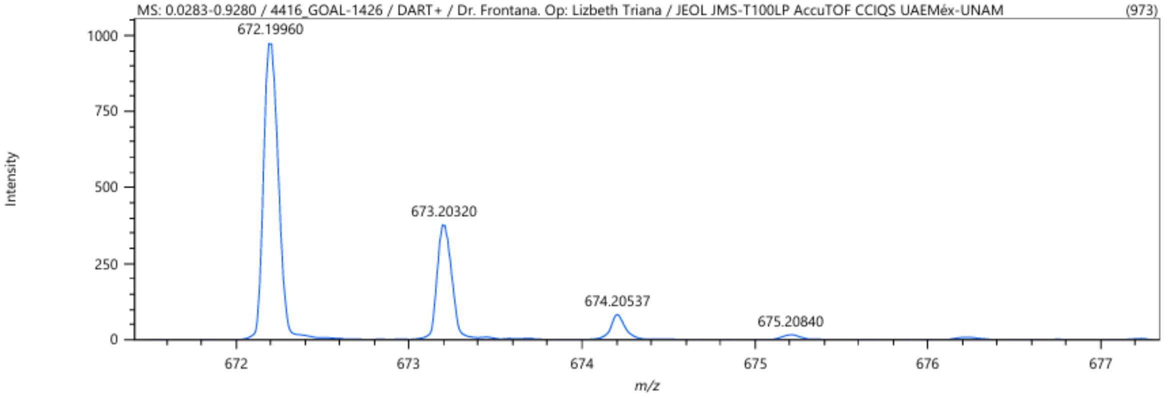

Elemental Composition

Parameters	
Tolerance:	±10.00 ppm
Electron:	Odd/Even
Charge:	+1
DBE:	-15.0 - 21.0

Elements Set 2:

Symbol	C	H	O	N	P
Min	0	0	0	0	0
Max	100	100	10	1	1

Results

Mass	Intensity	Intensity [%]	Formula	Calculated Mass	Mass Difference [mDa]	Mass Difference [ppm]	DBE
672.19960	973.16	3.03	C36 H35 N O10 P	672.19931	0.29	0.44	20.5

*N-alkoxyphthalimide (**4**)*

Spectrum

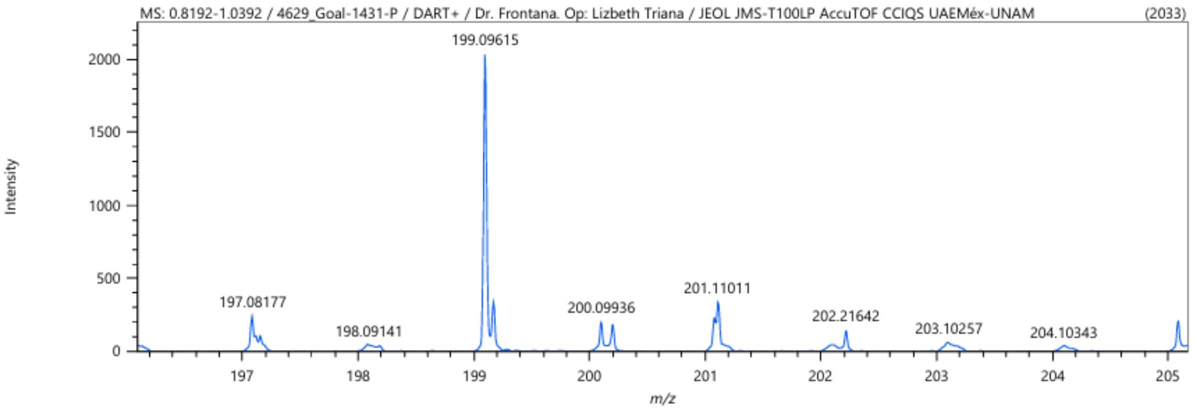

Elemental Composition

Parameters		Elements Set 4:				
Tolerance:	±3.00 ppm	Symbol	C	H	O	N
Electron:	Odd/Even	Min	0	0	0	0
Charge:	+1	Max	50	50	4	0
DBE:	-15.0 - 25.0					

Results

Mass	Intensity	Intensity [%]	Formula	Calculated Mass	Mass Difference [mDa]	Mass Difference [ppm]	DBE
199.09615	2033.13	1.80	C10 H15 O4	199.09649	-0.33	-1.67	3.5

Spectrum

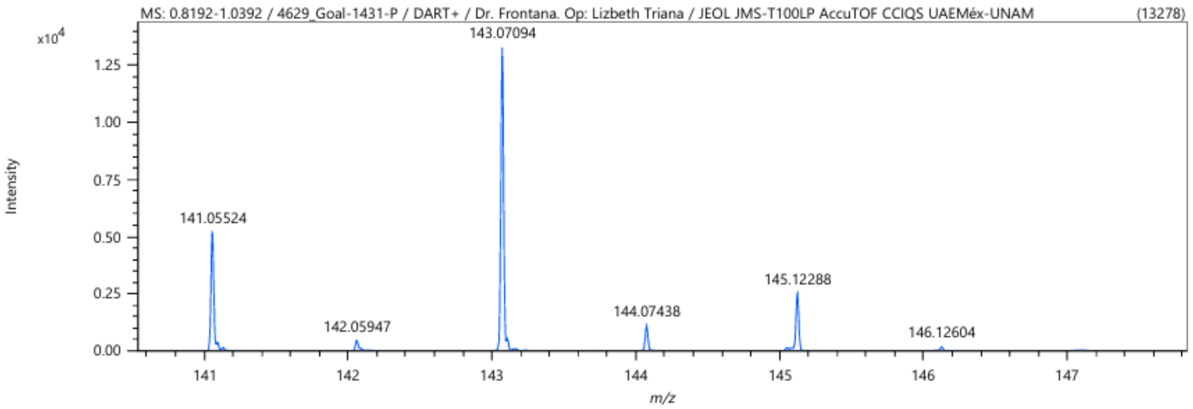

Elemental Composition

Parameters		Elements Set 4:				
Tolerance:	±5.00 ppm	Symbol	C	H	O	N
Electron:	Odd/Even	Min	0	0	0	0
Charge:	+1	Max	50	50	4	0
DBE:	-15.0 - 25.0					

Results

Mass	Intensity	Intensity [%]	Formula	Calculated Mass	Mass Difference [mDa]	Mass Difference [ppm]	DBE
143.07094	13277.56	11.78	C7 H11 O3	143.07027	0.67	4.66	2.5

(2S,3a'S,6a'R)-2',2'-dimethyltetrahydro-3H,6'H-spiro[furan-2,5'-furo[2,3-d][1,3]dioxole]
(3)

Spectrum

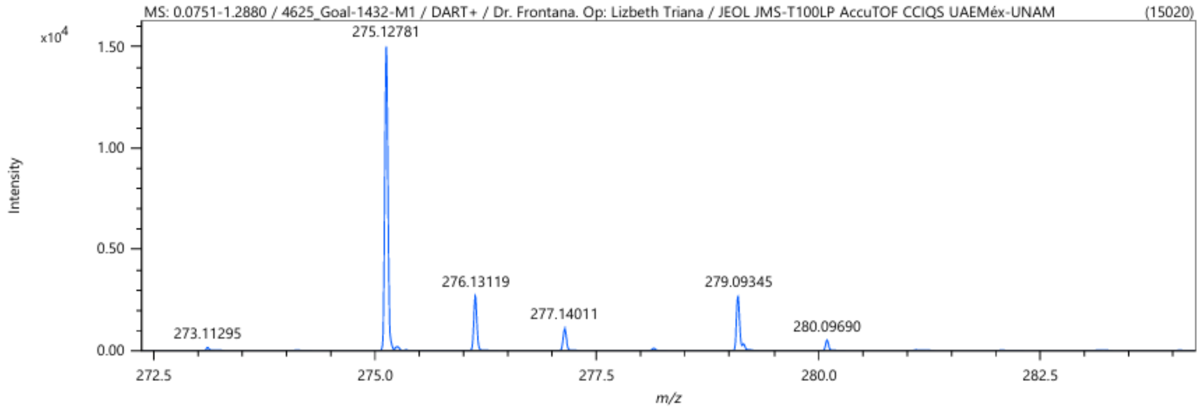

Elemental Composition

Parameters		Elements Set 4:				
Tolerance:	±3.00 ppm	Symbol	C	H	O	N
Electron:	Odd/Even	Min	0	0	0	0
Charge:	+1	Max	50	50	4	0
DBE:	-15.0 - 25.0					

Results

Mass	Intensity	Intensity [%]	Formula	Calculated Mass	Mass Difference [mDa]	Mass Difference [ppm]	DBE
275.12781	15020.43	15.75	C16 H19 O4	275.12779	0.02	0.08	7.5

Spectrum

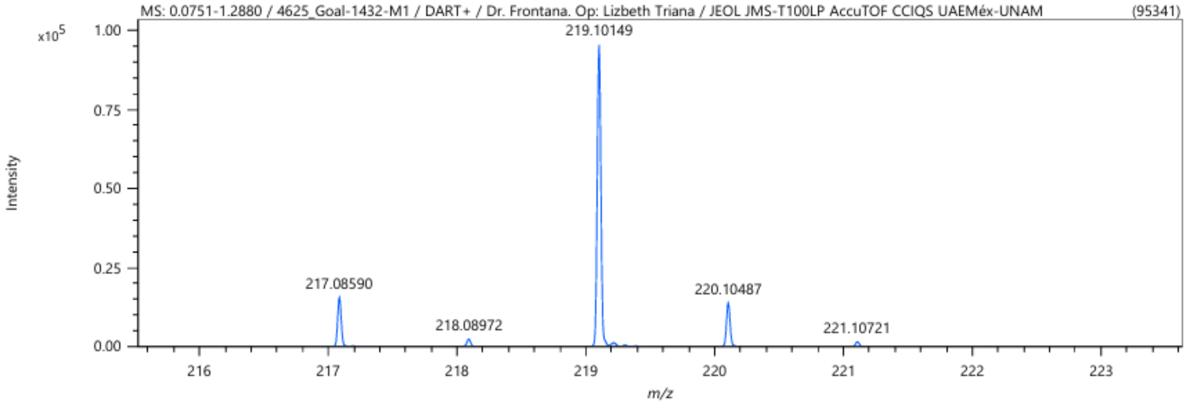

Elemental Composition

Parameters		Elements Set 4:				
Tolerance:	±3.00 ppm	Symbol	C	H	O	N
Electron:	Odd/Even	Min	0	0	0	0
Charge:	+1	Max	50	50	4	0
DBE:	-15.0 - 25.0					

Results

Mass	Intensity	Intensity [%]	Formula	Calculated Mass	Mass Difference [mDa]	Mass Difference [ppm]	DBE
219.10149	95341.03	100.00	C13 H15 O3	219.10157	-0.08	-0.36	6.5

(2S,3a'S,6a'R)-2',2'-dimethyl-5-phenyltetrahydro-3H,6'H-spiro[furan-2,5'-furo[2,3-d][1,3]dioxole] ***(5a)***

Spectrum

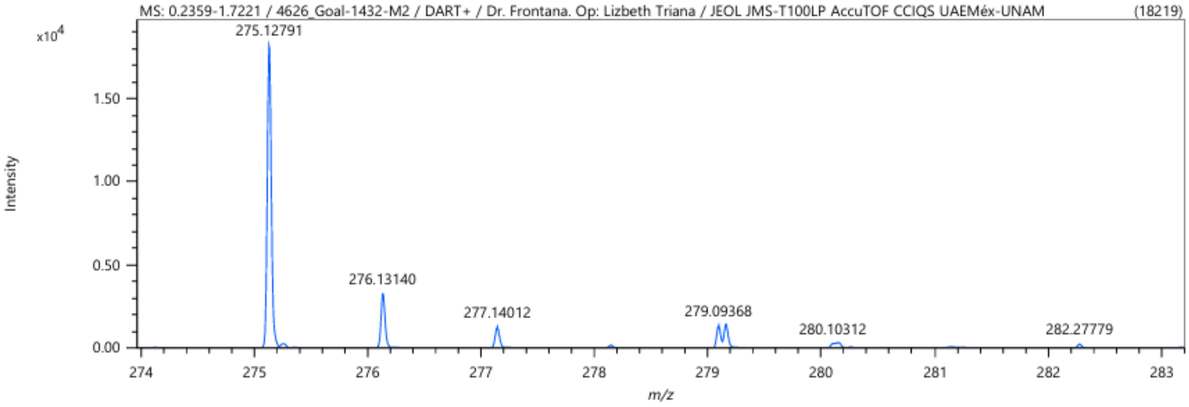

Elemental Composition

Parameters		Elements Set 4:				
Tolerance:	±3.00 ppm	Symbol	C	H	O	N
Electron:	Odd/Even	Min	0	0	0	0
Charge:	+1	Max	50	50	4	0
DBE:	-15.0 - 25.0					

Results

Mass	Intensity	Intensity [%]	Formula	Calculated Mass	Mass Difference [mDa]	Mass Difference [ppm]	DBE
275.12791	18218.98	19.22	C16 H19 O4	275.12779	0.13	0.46	7.5

Spectrum

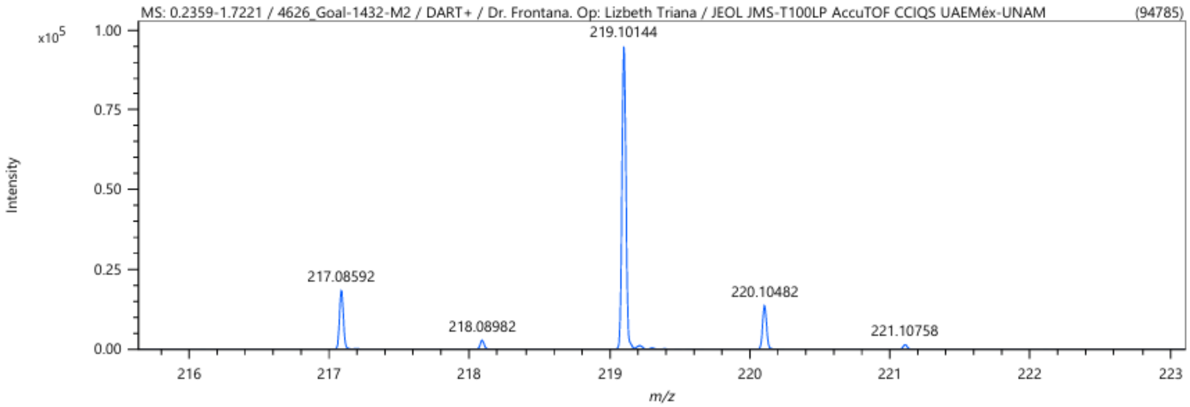

Elemental Composition

Parameters		Elements Set 4:				
Tolerance:	±3.00 ppm	Symbol	C	H	O	N
Electron:	Odd/Even	Min	0	0	0	0
Charge:	+1	Max	50	50	4	0
DBE:	-15.0 - 25.0					

Results

Mass	Intensity	Intensity [%]	Formula	Calculated Mass	Mass Difference [mDa]	Mass Difference [ppm]	DBE
219.10144	94784.66	100.00	C13 H15 O3	219.10157	-0.13	-0.61	6.5

*(2S,3a'S,6a'R)-2',2'-dimethyl-5-phenyltetrahydro-3H,6'H-spiro[furan-2,5'-furo[2,3-d][1,3]dioxole] (**5b**)*

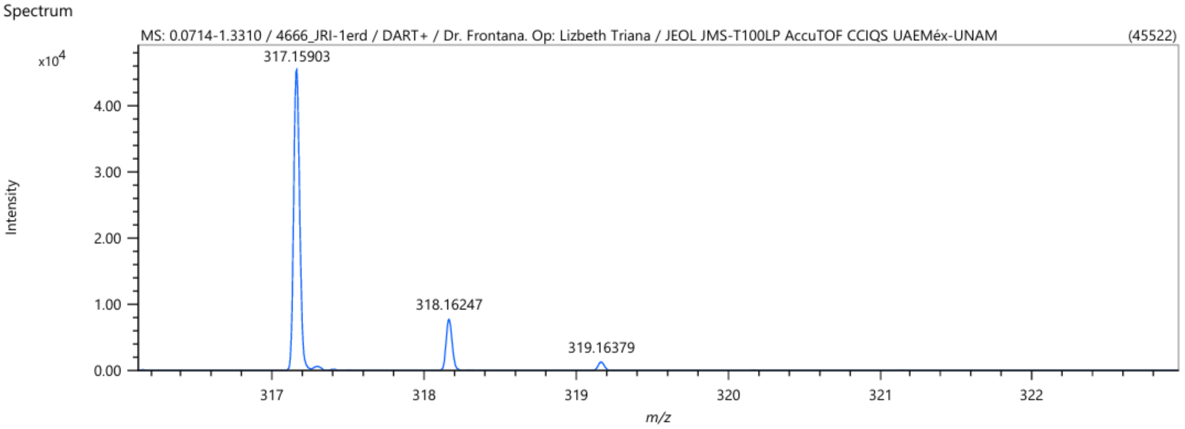

Elemental Composition

Parameters	
Tolerance:	±3.00 ppm
Electron:	Odd/Even
Charge:	+1
DBE:	-15.0 - 21.0

Elements Set 3:

Symbol	C	H	O	N
Min	0	0	0	0
Max	50	50	7	0

Results

Mass	Intensity	Intensity [%]	Formula	Calculated Mass	Mass Difference [mDa]	Mass Difference [ppm]	DBE
317.15903	45521.66	100.00	C15 H25 O7	317.15948	-0.45	-1.43	3.5

*Methyl 2-((3aR,4S,6R,6aS)-6-((R)-2,2-dimethyl-1,3-dioxolan-4-yl)-2,2-dimethyltetrahydrofuro[3,4-d][1,3]dioxol-4-yl)acetate (**12b**)*

INSTITUTO DE QUIMICA, UNAM
LABORATORIO DE ESPECTROMETRIA DE MASAS

Data:61_STA-4000_JRI-143-D1
Sample Name:DR Bernardo Frontana / Operador : Carmen Garcia
Description:
Ionization Mode:ESI+
History:Determine m/z[Peak Detect[Centroid,30,Area];Correct Base[5.0%]];Correct Base[5.0%];Average(MS[1] 1..1)

Acquired:6/10/2024 11:35:35 AM
Operator:AccuTOF
Mass Calibration data:CAL_PEG_600_JEOL_2024060...
Created:6/10/2024 1:07:26 PM
Created by:AccuTOF

Charge number:1
Element:^{12}C:0 .. 15, ^{1}H:0 .. 30, ^{16}O:0 .. 6

Tolerance:3.00(ppm), 5.00 .. 15.00(mmu)

Unsaturation Number:-0.5 .. 100.0 (Fraction:Both)

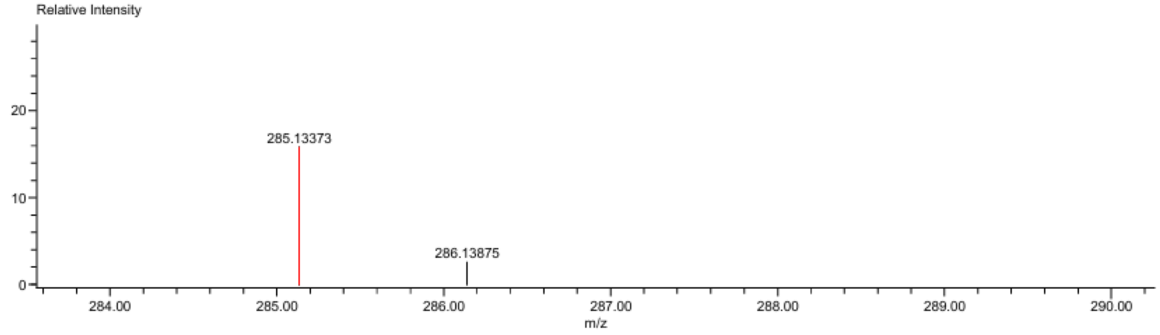

Mass	Intensity	Calc. Mass	Mass Difference (mmu)	Mass Difference (ppm)	Possible Formula	Unsaturation Number
285.13373	34500.92	285.13381	-0.09	-0.31	$^{12}C_{14}{}^{1}H_{21}{}^{16}O_6$	4.5

*Methyl 2-((3aR,4S,6R,6aS)-2,2-dimethyl-6-(3-oxobut-1-en-1-yl)tetrahydrofuro[3,4-d][1,3]dioxol-4-yl)acetate (minor-**9**)*

Spectrum

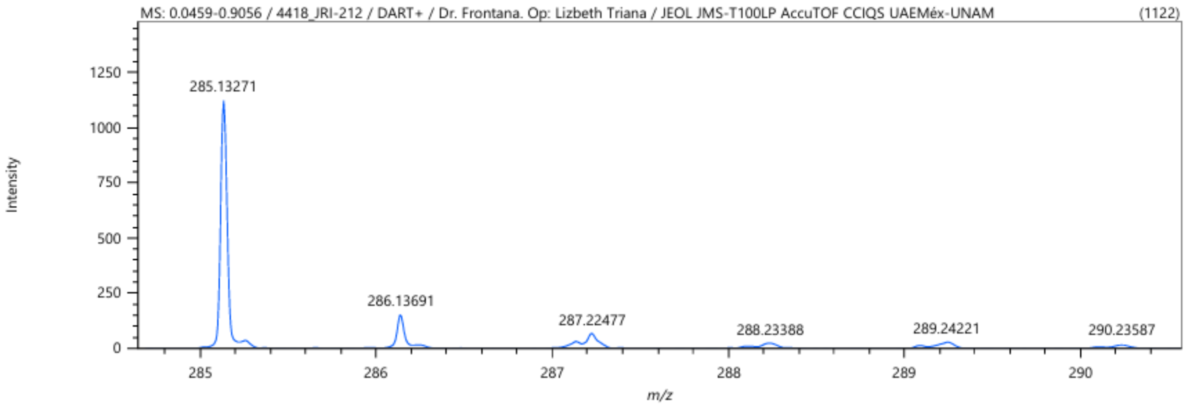

Elemental Composition

Parameters	
Tolerance:	±10.00 ppm
Electron:	Odd/Even
Charge:	+1
DBE:	-15.0 - 21.0

Elements Set 2:

Symbol	C	H	O	N
Min	0	0	0	0
Max	100	100	6	0

Results

Mass	Intensity	Intensity [%]	Formula	Calculated Mass	Mass Difference [mDa]	Mass Difference [ppm]	DBE
285.13271	1121.64	0.61	C14 H21 O6	285.13326	-0.55	-1.94	4.5

*Methyl 2-((3aR,4S,6R,6aS)-2,2-dimethyl-6-(3-oxobut-1-en-1-yl)tetrahydrofuro[3,4-d][1,3]dioxol-4-yl)acetate (major-**9**)*

INSTITUTO DE QUIMICA, UNAM
LABORATORIO DE ESPECTROMETRIA DE MASAS

Data:63_STA-4002_JRI-146-F3
Sample Name:DR Bernardo Frontana / Operador : Carmen Garcia
Description:
Ionization Mode:ESI+
History:Determine m/z[Peak Detect[Centroid,30,Area];Correct Base[5.0%]];Correct Base[5.0%];Average(MS[1] 1..1)

Acquired:6/10/2024 11:47:01 AM
Operator:AccuTOF
Mass Calibration data:CAL_PEG_600_JEOL_2024060...
Created:6/10/2024 1:14:21 PM
Created by:AccuTOF

Charge number:1
Element:^{12}C:0 .. 15, ^{1}H:0 .. 30, ^{16}O:0 .. 6

Tolerance:3.00(ppm), 5.00 .. 15.00(mmu)

Unsaturation Number:-0.5 .. 100.0 (Fraction:Both)

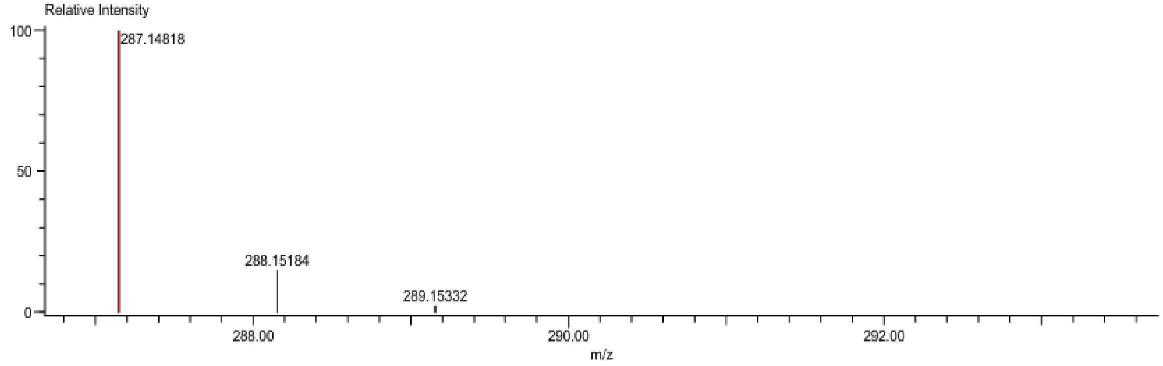

Mass	Intensity	Calc. Mass	Mass Difference (mmu)	Mass Difference (ppm)	Possible Formula	Unsaturation Number
287.14818	65988.63	287.14946	-1.28	-4.45	$^{12}C_{14}{}^{1}H_{23}{}^{16}O_{6}$	3.5

*Methyl 2-((3aR,4S,6R,6aS)-2,2-dimethyl-6-(3-oxobutyl)tetrahydrofuro[3,4-d][1,3]dioxol-4-yl)acetate (**S10**)*

Spectrum

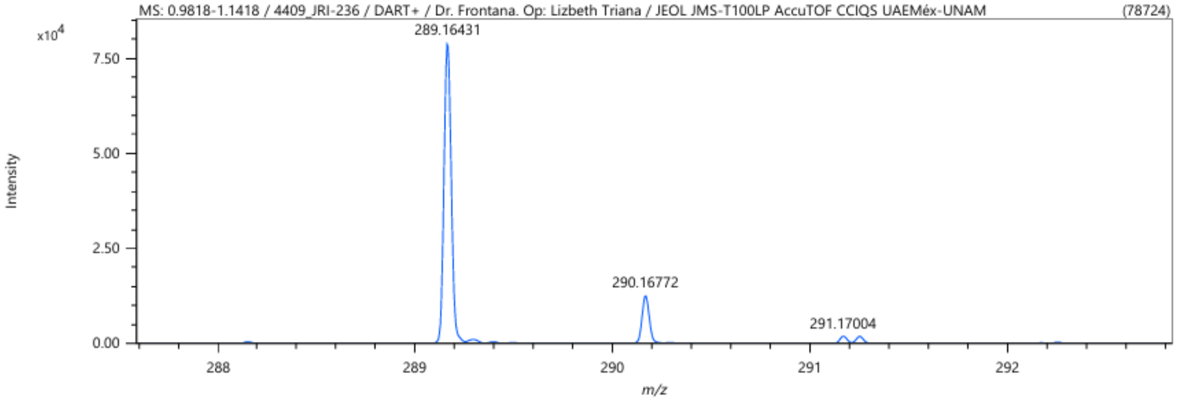

Elemental Composition

Parameters		Elements Set 6:				
Tolerance:	±10.00 ppm	Symbol	C	H	N	O
Electron:	Odd/Even	Min	0	0	0	0
Charge:	+1	Max	50	50	0	6
DBE:	-15.0 - 20.0					

Results

Mass	Intensity	Intensity [%]	Formula	Calculated Mass	Mass Difference [mDa]	Mass Difference [ppm]	DBE
289.16431	78724.29	100.00	C14 H25 O6	289.16456	-0.25	-0.88	2.5

*Methyl 2-((3aR,4S,6R,6aS)-6-((S)-3-hydroxybutyl)-2,2-dimethyltetrahydrofuro[3,4-d][1,3]dioxol-4-yl)acetate (**14**)*

Spectrum

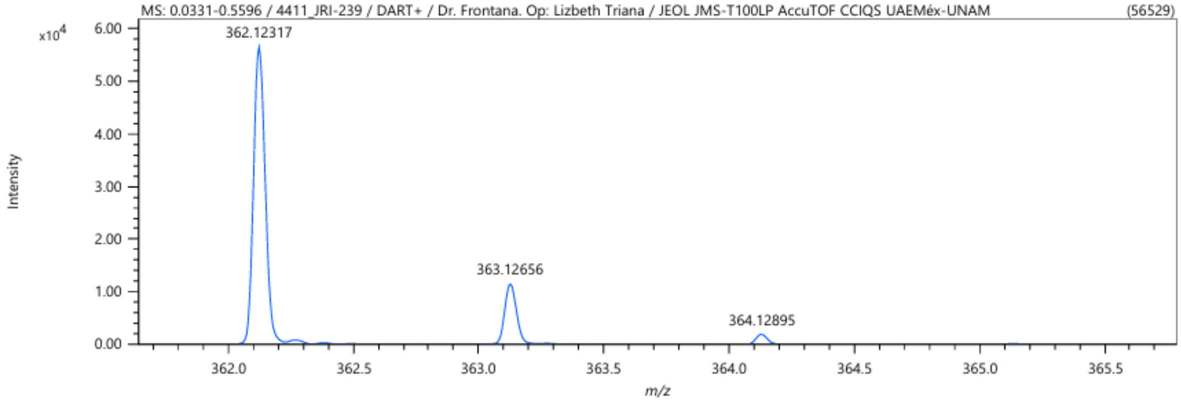

Elemental Composition

Parameters		Elements Set 6:				
Tolerance:	±10.00 ppm	Symbol	C	H	N	O
Electron:	Odd/Even	Min	0	0	0	0
Charge:	+1	Max	50	50	1	7
DBE:	-15.0 - 20.0					

Results

Mass	Intensity	Intensity [%]	Formula	Calculated Mass	Mass Difference [mDa]	Mass Difference [ppm]	DBE
362.12317	56528.73	100.00	C18 H20 N O7	362.12343	-0.26	-0.73	9.5

*N-alkoxyphthalimide (**15**)*

Spectrum

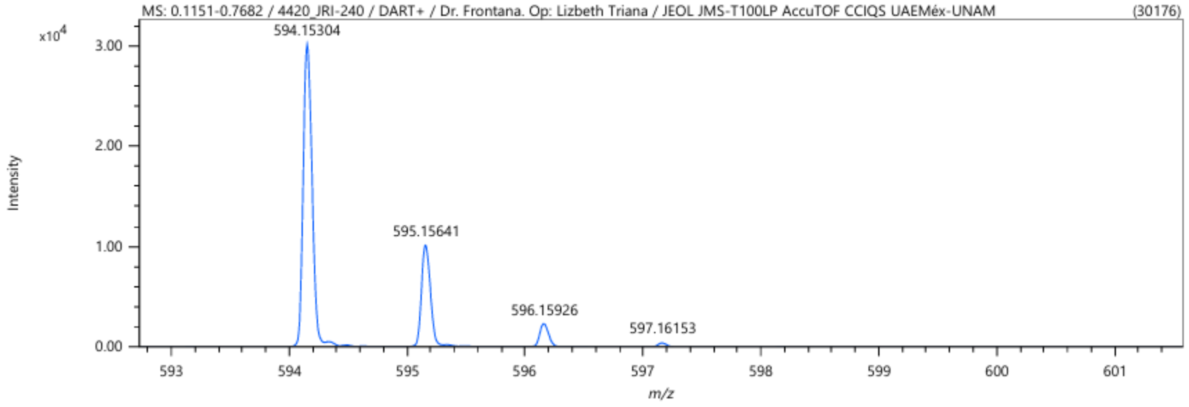

Elemental Composition

Parameters		Elements Set 2:					
Tolerance:	±10.00 ppm	Symbol	C	H	O	N	P
Electron:	Odd/Even	Min	0	0	0	0	0
Charge:	+1	Max	100	100	10	1	1
DBE:	-15.0 - 21.0						

Results

Mass	Intensity	Intensity [%]	Formula	Calculated Mass	Mass Difference [mDa]	Mass Difference [ppm]	DBE
594.15304	30175.85	100.00	C30 H29 N O10 P	594.15236	0.68	1.15	17.5

*N-alkoxyphthalimide (**7**)*

Spectrum

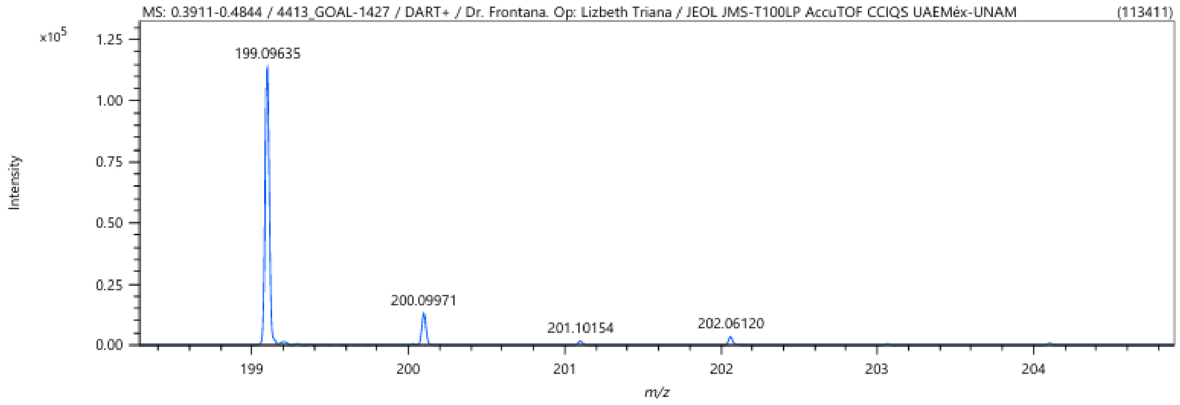

Elemental Composition

Parameters		Elements Set 2:				
Tolerance:	±10.00 ppm	Symbol	C	H	O	N
Electron:	Odd/Even	Min	0	0	0	0
Charge:	+1	Max	100	100	4	0
DBE:	-15.0 - 11.0					

Results

Mass	Intensity	Intensity [%]	Formula	Calculated Mass	Mass Difference [mDa]	Mass Difference [ppm]	DBE
199.09635	113411.20	100.00	C10 H15 O4	199.09649	-0.14	-0.69	3.5

(-)-Cephalosporolide F + C9-epimer

Spectrum

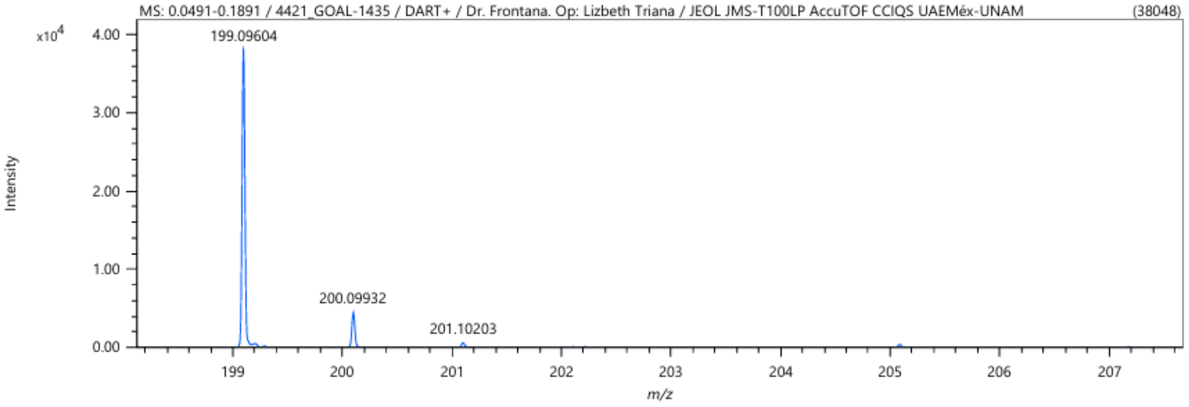

Elemental Composition

Parameters	
Tolerance:	±10.00 ppm
Electron:	Odd/Even
Charge:	+1
DBE:	-15.0 - 21.0

Elements Set 2:

Symbol	C	H	O	N
Min	0	0	0	0
Max	100	100	4	0

Results

Mass	Intensity	Intensity [%]	Formula	Calculated Mass	Mass Difference [mDa]	Mass Difference [ppm]	DBE
199.09604	38047.75	100.00	C10 H15 O4	199.09649	-0.45	-2.24	3.5

(+)-Cephalosporolide F
